# Battery smart sensing via a virtual reference electrode

**DOI:** 10.1038/s41467-026-76535-y

**Published:** 2026-08-11

**Authors:** Jiayi Yu, Aki Takahashi, Min-Ho Kim, Rishi Upadhyay, Howard Zhang, Tian-Yu Wang, Huayang Zhu, Robert Kee, Tyrone Vincent, Bo Liu, Xintong Yuan, Thomas Hymel, Kaiyan Liang, Keyue Liang, Haoyang Wu, Dingyi Zhao, Jung Tae Kim, Achuta Kadambi, Yuzhang Li

**Affiliations:** 1 Department of Chemical and Biomolecular Engineering, University of California, Los Angeles, Los Angeles, CA USA; 2 Department of Mechanical and Aerospace Engineering, University of California, Los Angeles, Los Angeles, CA USA; 3 Department of Energy Engineering, Korea Institute of Energy Technology, Naju, Republic of Korea; 4 Department of Electrical Engineering and Computer Science, University of California, Los Angeles, Los Angeles, CA USA; 5 Department of Mechanical Engineering, Colorado School of Mines, Golden, CO USA; 6 Department of Electrical Engineering, Colorado School of Mines, Golden, CO USA; 7 Department of Materials Science and Engineering, Stanford University, Stanford, CA USA

**Keywords:** Batteries, Energy, Batteries, Energy modelling

## Abstract

Battery safety is regulated by management systems that use current and cell voltage to define operating limits, but these signals can miss lithium metal plating. Negative electrode potential can reveal this failure mode, yet direct measurement requires an additional reference electrode that is difficult to implement in practical cells. Here, we introduce a virtual reference electrode that estimates negative electrode potential during battery operation without a physical reference electrode. Trained on measured negative electrode potentials from three-electrode cells, the model predicts this internal state using only signals available from two-electrode cells, with root-mean-squared and mean absolute errors of 0.023 and 0.018 volts, respectively. Electron microscopy validates the predicted transition between intercalation and lithium metal plating near the thermodynamic threshold. Integrated into adaptive charging, the virtual reference electrode adjusts current in response to predicted failure risk and extends cycle life by 8.25 times relative to a constant-current constant-voltage baseline under the tested low negative-to-positive capacity ratio. More broadly, this framework may enable virtual sensing of internal battery states without changing battery chemistry or architecture.

## Abstract (web summary)

Battery safety is limited by hidden lithium plating. Here, authors use machine learning to build a virtual reference electrode that estimates negative electrode potential from standard cell signals, detects plating risk and enables an 8.25-fold extension in cycle life.

## Introduction

**Fig. 1:**
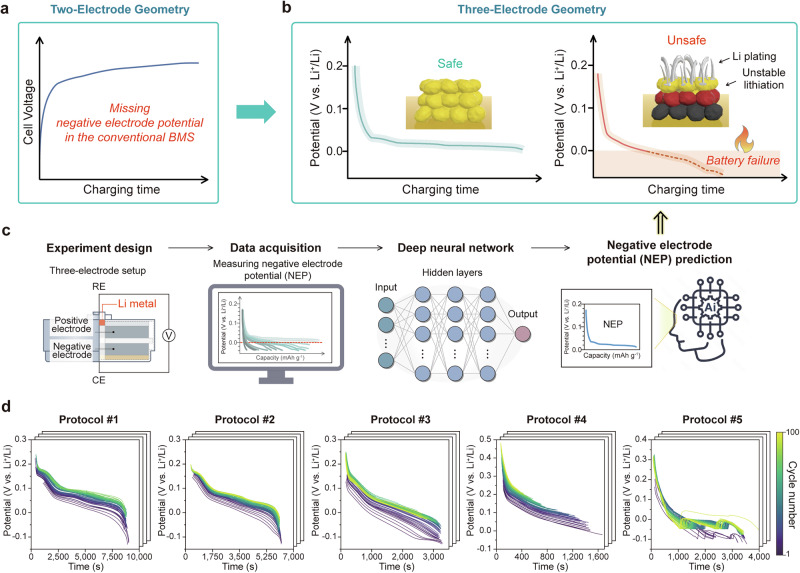
Building an ML-powered virtual reference electrode. **a** Absence of negative electrode potential information in conventional 2E cells. **b** Two lithiation modes on the graphite negative electrode can be detected in 3E geometry. Left: Reversible Li intercalation with positive negative electrode potential; Right: Catastrophic Li plating with negative negative electrode potential. **c** Workflow of building a virtual reference electrode, from 3E cell geometry and NEP data collection, to model training, and real-time prediction. WE working electrode, CE counter electrode, RE reference electrode. **d** Overview of our 3E dataset with 9.8 M data points, which tracks negative electrode potential throughout battery lifetime.

Establishing an optimal and safe battery charging protocol represents a multi-billion-dollar decision for battery manufacturers, as the risk of recall due to unsafe operation can have substantial safety and economic consequences^1,2^. Charging protocols rely on battery management systems (BMS) that translate measurable parameters (e.g., cell voltage, current) into estimates of safe or unsafe operation, adjusting the control parameters as necessary. However, conventional battery management systems can miss failure modes such as metallic lithium plating since standard measurables give limited physical insight into battery degradation mechanisms, thus making conventional charging protocols highly empirical and rigid^3,4^. For example, while cell voltage is widely used to monitor battery health and state of charge, it does not provide physical insight into whether or when metallic Li plating occurs on the negative electrode. This makes it difficult for a BMS to define battery safety limits based on cell voltage alone (Fig. 1a). Due to these limitations, current BMS systems are often constrained to either conservative battery operation modes that sacrifice performance^5,6^ or higher rate protocols that may fail to detect hazardous failure modes in time^7–9^.

Intelligent battery operation through a smart BMS hinges on the ability to reliably sense parameters that reflect the fundamental physics governing battery degradation and failure. For example, undesired metallic Li plating at the graphite negative electrode (the dominant material in today’s Li-ion batteries) is thermodynamically favored when the negative electrode potential (NEP) falls below 0 V vs. Li^0^/Li^+^^9,10^. This is analogous to the spontaneous freezing of water below 0 °C. To avoid this failure mode that may lead to battery fire or even explosion, the NEP can serve as a physically interpretable parameter that precisely defines safety limits of normal operation (i.e., Li intercalation above 0 V vs. Li^0^/Li^+^) and catastrophic failure^11,12^ (i.e., Li metal plating below 0 V vs. Li^0^/Li^+^). Unfortunately, the direct measurement of NEP relies on the use of a third reference electrode^13–18^ (Fig. 1b), which poses challenges to scalable battery manufacturing and integration into commercial battery systems^19^. Other physically interpretable parameters that detect Li plating (e.g., pressure^20–22^, volume and thickness^23,24^) face similar challenges, or lack the capability for real-time prediction (e.g., voltage at end-of-charge^25^, which may indicate Li plating only after it has occurred). Although physics-based models (PBMs) that simulate NEP based on simplified transport and interfacial kinetics can reproduce early-cycle behavior, they are computationally intensive and rely on static or empirically assumed parameter sets, making it difficult to capture dynamic degradation phenomena such as evolving impedance, lithium inventory loss, and interfacial damage that span the chemical, thermal, and mechanical heterogeneities over the full lifetime of a battery due to its static or assumed parameter sets. A real physical reference electrode would be expected to be accurate throughout the lifetime of a cell and respond to dynamically changing parameters (e.g., current). Thus, a virtual reference electrode should therefore offer comparable fidelity, maintaining prediction accuracy while enabling real-time, on-the-fly estimation of negative electrode potential throughout operation. Indeed, the accuracy of such physics-based approaches in predicting NEP throughout long-term cycling is rarely validated with experimental evidence or visualized graphically beyond the 5^th^ cycle, further highlighting the difficulty in predicting physically meaningful parameters over extended battery operation^26–29^. Therefore, additional approaches are needed to accurately predict the NEP continuously throughout battery operation without direct measurement. Such an approach could improve battery management, as operating modes can then be dynamically adjusted and individually tailored in response to safety-relevant failures before they occur, which is in stark contrast to the empirical operating protocols that dominate conventional BMS.

Here, we introduce the concept of a virtual reference electrode that can dynamically and continuously monitor the NEP of a 2-electrode (2E) cell throughout its lifetime without the need for a physical 3^rd^ reference. Our virtual reference electrode is powered by deep learning models that are trained and validated on a large dataset (9.8 M data points) of 3-electrode (3E) coin cells cycled under a variety of conditions. This machine-learning (ML) powered virtual reference electrode takes only features available in 2E cells and outputs the predicted NEP. While physics-based approaches can be limited in modeling the complexity of battery aging and cannot provide reliable predictions of NEP beyond early cycles, our deep learning models are trained on measured data that is closer to the ground truth. We report an overall root mean squared error (RMSE) of 0.023 V with a mean absolute error (MAE) of 0.018 V when averaged across our test set, with no degradation in accuracy between early and late cycles. The accuracy of our virtual reference electrode is further validated experimentally with scanning electron microscopy (SEM), which indicates the absence of Li metal plating at a predicted NEP near the thermodynamic threshold (i.e., 0.01 V vs. Li^0^/Li^+^) and the presence of Li metal plating at a predicted NEP just below the thermodynamic threshold (i.e., −0.02 V vs. Li^0^/Li^+^). With our ML-powered virtual reference, we design a smart BMS that can respond dynamically to predicted failure modes without the need for additional sensing equipment, allowing us to improve battery operation under the tested conditions without compromising safety or stability. In particular, while low N/P ratios (i.e., the ratio of the capacity of the negative electrode to the positive electrode) can enable increased energy density and utilization efficiency of materials, increased likelihood of Li metal plating typically limits minimum N/P ratios to 1.1 or higher^30,31^. When cycling 2E coin cells with a low N/P ratio (1.0), we observe an 8.25x enhancement in cycle life using our smart charging protocol (e.g., BMS equipped with ML virtual reference electrode) compared to a conventional charging baseline (i.e., constant-current-constant-voltage, CC-CV). We show that this cycle-life increase is enabled by our virtual reference electrode’s ability to detect and avoid Li metal plating throughout battery cycling. Our concept of a virtual reference electrode can be broadened to include other internal battery parameters that have physical meaning but are difficult to measure at scale (e.g., pressure volume). Together, such virtual sensors powered by deep learning may help identify safety-relevant internal states and may support battery health sensing and management, transforming how battery safety and optimization are managed.

## Results

### Data generation and machine-learning model performance

A deep learning model that can accurately predict the NEP throughout a battery’s lifetime requires a large dataset of measured NEP values, which represent the ground truth. To address this, we leverage a hand-made 3E coin cell architecture (Fig. 1c and Supplementary Fig. 1) that enables high-throughput and consistent battery cycling while also allowing us to measure the NEP (Supplementary Fig. 3 and Method). In this study, we select lithium iron phosphate (LFP) and graphite (Gr) as a model battery chemistry to build our 3E dataset. Predicting how close a battery is to failure risk (e.g., negative electrode potential approaching 0 V) is particularly difficult in Gr | |LFP systems due to their characteristically flat cell voltage profiles throughout battery operation^32–34^. Thus, the ability to accurately predict NEP in this challenging model system suggests the utility of the virtual reference electrode in sensing battery failure and state-of-health. Using our 3E coin cell architecture, we cycle 55 Gr | |LFP cells under a diverse set of charging conditions (see Methods, Fig. S2 and Table S1 in Supplementary Information), generating approximately 10 M data points of NEP (Fig. 1d). Specifically, we modulate the constant-current (CC) regime of a typical constant-current constant-voltage (CC-CV) charging protocol while maintaining a consistent CV regime and discharging steps. The CC regime in our dataset spans current densities from 0.79 mA cm^−2^ to 2.37 mA cm^−2^ and also contains cells with stepped CC, in which the charging current is incrementally decreased throughout the CC regime to mimic a more time-varying charging profile^35^. Together, this initial dataset captures a broad range of typical battery operating conditions, allowing us to train an ML model capable of predicting the NEP throughout the charging process. Beyond the scope of this work, the dataset also provides a valuable foundation for long-term quantitative calibration and validation of underlying PBMs.

**Fig. 2:**
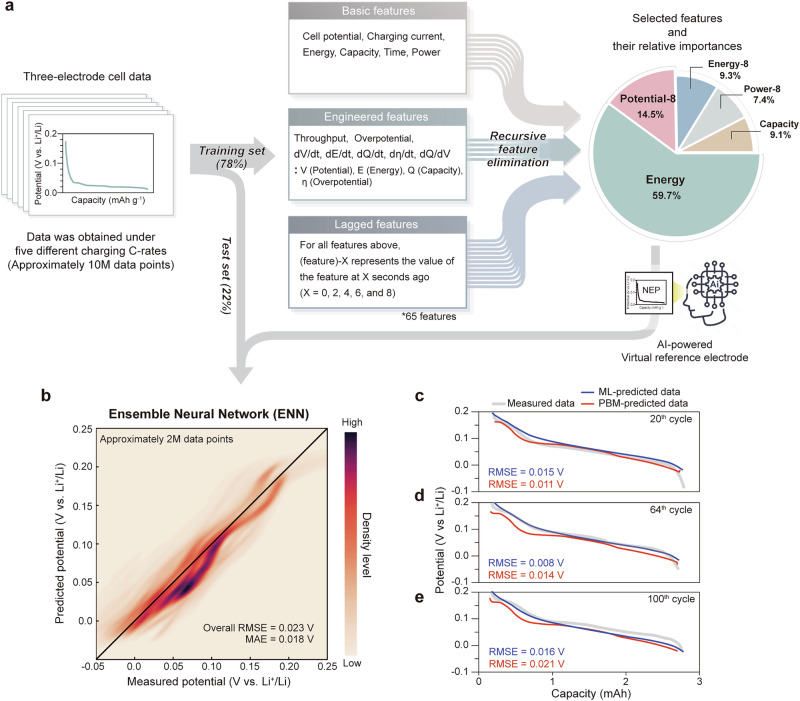
Feature selection workflow and overall performance of virtual reference. **a** Workflow of feature engineering and selection. 3E cell data was first split into training and test sets (78%/22%), then 65 features were created according to battery-domain knowledge. Finally, 5 features were selected via RF-RFE. **b** 2D density plot of predicted NEP versus measured NEP across all cells in test set; Predicted and measured NEP comparison at **c** cycle 20, **d** cycle 64 (best individual-cycle performance), and **e** cycle 100 of cell #2 in test set. Measured NEP, ML-predicted NEP, and PBM-predicted NEP are shown in gray, blue, and red, respectively. ML machine learning, PBM physics-based model, RMSE root-mean-square error, MAE mean absolute error. All electrochemical tests were performed at 25 °C. Source data are provided as a Source Data file.

To maximize the utility of our ML-powered virtual reference electrode, we seek to develop an ML model with high accuracy and robustness while retaining interpretability and compatibility with large datasets (Fig. 1c). Indeed, 3E battery cycling at a commercial scale would easily contain billions of data points, which would further enhance the accuracy of our ML model if it can scale with the dataset. To build our ML model, we first leverage battery domain knowledge to generate a comprehensive set of 65 features (all of which are measurable or calculable in conventional 2E cells; Details are included in Table S3 and Supplementary Note 2) that are related to NEP (Fig. 2a). This large pool of features encompasses (1) directly measurable parameters (e.g., cell voltage, current, capacity), (2) engineered features (e.g., throughput, overpotential), and (3) time-lagged features (e.g., features that are measurable and engineered features from past timesteps up to 8 s prior, with lagged features considered at 2 s intervals consistent with the smart-charging sampling rate discussed later in the main text). These diverse features are intended to capture the physical and dynamic signatures that may be salient in predicting NEP. After partitioning the dataset into training and test sets (78%/22% split), we run random forest recursive feature elimination^36–38^ (RF-RFE) on the training set. This dimensionality reduction strategy helps us mitigate overfitting and select the most informative predictors. After running RFE, we select the five features identified as the most important. And use them to train and test our model (Fig. 2a). We found that an ensemble of five feedforward neural networks (see Machine learning model development in Supplementary Information) showed lower prediction error than the other tested model architectures (see Supplementary Fig. 4). Model hyperparameters, including hidden-layer dimension, number of hidden layers, and ensemble size, were systematically tuned based on validation-set performance to identify a final architecture that balanced prediction accuracy and model compactness (see Hyperparameter Tuning in Supplementary Information and Supplementary Fig. 13 and Tables S10, 11). This model yielded an overall root mean square error (RMSE) of 0.023 V on the test dataset, a mean absolute error (MAE) of 0.018 V (Fig. 2b) (2.2 M data points), and a minimum single-cycle RMSE of 0.008 V (Fig. 2d). Not only are these error metrics competitive with even the reported physics-based simulations^26,39,40^, but also remain robust throughout battery lifetime without a decay in accuracy across multiple C-rates (see Supplementary Fig. 6). This is largely due to the variety of cycling conditions and aging data included in our training set. Furthermore, the consistency of model performance indicates the robustness of our model against overfitting. To enable a fair comparison between the ML and physics-based approaches, we implemented NEP prediction using a P2D model calibrated specifically to our 3E coin-cell system (Fig. 2c–e and Methods in Physics-based simulation on negative electrode potential part). Notably, for the ML approach, the single-cycle RMSE at cycle 20 (0.015 V, the blue curve in Fig. 2c) is nearly identical to that of cycle 100 (0.016 V, the blue curve in Fig. 2e), underscoring the consistency of our model over long-term cycling. These predictions are directly compared with measured three-electrode data. For PBMs, the prediction error at cycle 20 is comparable to, and even slightly lower than, that of the ML model (0.011 V; red curve in Fig. 2c). However, as the battery ages, the error progressively increases and eventually exceeds that of our ML model (0.014 V at cycle 64 and 0.021 V at cycle 100; red curves in Fig. 2d, e). This trend highlights the advantage of the ML approach in forecasting long-term degradation phenomena using real-world battery datasets that inherently capture diverse aging behaviors. Furthermore, to validate the robustness of our ML model when encountering a charging rate that does not exist in the training set, we trained 2 additional models where we purposefully hold out all cells with specific C-rates during training and then evaluate our model on those cells (Table S4). These additional models exhibit performance comparable to the base model, demonstrating our robustness in predicting under different C-rate conditions. To further assess whether cell-to-cell batch variability affects model robustness, we evaluated prediction performance consistency across 8 cells in the test set deliberately selected from different experimental batches using the same trained ML model without per-cell calibration, retraining, or fine-tuning (Table S9). The results indicate that the model maintains comparable prediction accuracy across unseen cells, supporting its applicability to new cells across different batches. Despite our extensive data collection, our dataset is relatively modest in size compared to those used to train time-series and foundation models in other domains. Therefore, we anticipate that the accuracy of our virtual reference electrode will continue to increase with larger and more diverse datasets. While this study primarily introduces the conceptual framework of a virtual reference electrode rather than optimizing its accuracy, we emphasize that data-driven ML approaches will continue to benefit from expanded datasets, computational resources, and in-depth physical interpretation from PBMs as well, as they provide complementary strengths for battery modeling.

Beyond simply building a high-performing but opaque (black box) ML model, understanding how and why our resulting model functions effectively will further bolster confidence in its predictive capabilities. From battery domain knowledge, we expect certain features to strongly correlate with NEP. In particular, cell voltage is inherently embedded in the very definition of NEP (i.e., the difference between positive electrode voltage and cell voltage) and thus should be an important input feature for our model. Additionally, the state of lithiation of the graphite, which governs the NEP, can be thermodynamically inferred from the instantaneous single-cycle cell capacity, which provides an additional feature we expect to be predictive for negative electrode potential. Interestingly, all five features selected through the RF-RFE process (see pie chart in Fig. 2a) directly contain either cell voltage and/or capacity, suggesting that our ML model might be able to learn the underlying physics of predicting NEP that aligns with our domain knowledge. Among them, the most important feature selected with a relative importance of 59.7% was Energy, a feature that captures the synergistic influence of both cell voltage and capacity on NEP through its very definition (energy is the product of voltage and capacity). Time-lagged features such as Voltage-8 (i.e., voltage value 8 s prior to the present measurement) were also selected as important features by RFE. Collectively, Voltage-8, Energy-8, and Power-8 have a relative feature importance of 31.2%, indicating that the present NEP prediction depends on these parameters measured (or calculated) 8 s in the past. This suggests that NEP prediction relies not only on instantaneous values, but also on historical temporal information, and that our ML model is capable of capturing the voltage relaxation behavior derived from temporal effects of reaction kinetics in our battery system, which would manifest as a delay in response time between the measured NEP and the degree of lithiation in the graphite negative electrode (i.e., its measured capacity), especially for cells with stepped CC protocol in our dataset^41^. It is interesting to note that RF-RFE did not select engineered features such as overpotential or dQ/dV that were considered in previous physics-based simulations of NEP or Li plating detection^39,42^, and the ML model trained using both of them showed poor performance on NEP prediction (see Supplementary Fig. 9). This may be due to the mismatch between the monotonically decreasing NEP and non-monotonically varying engineered features during the charging process^26,43–45^ (see Supplementary Fig. 5). This interplay warrants future study.

Overall, our analysis of the selected features for NEP prediction provides an additional lens to evaluate whether our ML model is performing well due to random chance or learning patterns that make physical sense. Together, the 5 selected features align with our fundamental understanding of battery operation, and future work might combine physics-informed ML^46^ to further improve its prediction accuracy.

### Real-time validation of the ML model

**Fig. 3:**
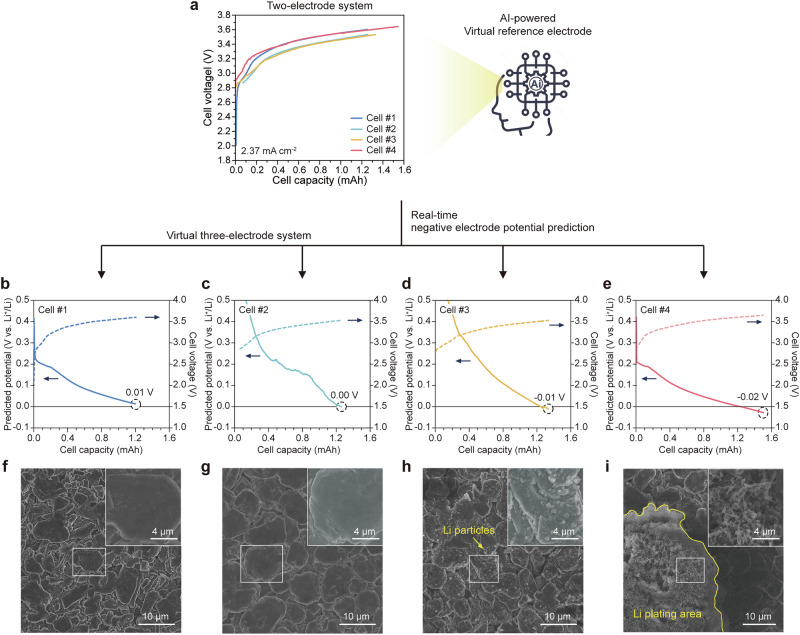
Experimental validation of the ML-powered virtual reference electrode. **a** Absence of negative electrode potential in 2E system without virtual reference electrode; Cell voltage & predicted NEP plots with virtual reference electrode under predicted NEP of **b** 0.01 V, **c**, 0 V, **d** −0.01 V, **e** −0.02 V; SEM images of the top surface of graphite negative electrode at predicted NEP of **f** 0.01 V, **g** 0 V, **h** −0.01 V, **i** −0.02 V. For ex situ SEM characterization in **f**–**i**, two-electrode Gr | |LFP cells were charged at 1 C and stopped when the predicted negative electrode potential reached 0.01 V, 0 V, −0.01 V, and −0.02 V, respectively, before cell disassembly and sample collection. All electrochemical tests were performed at 25 °C. Source data are provided as a Source Data file.

While the metrics described above highlight the accuracy of our virtual reference electrode in mathematical terms, a direct validation would be to examine its function during real-time, continuous battery operation. In principle, our ML-powered virtual reference electrode should function similarly to a physical reference electrode. For example, Li metal plating is thermodynamically favorable at an NEP below 0 V vs. Li metal, which can be directly measured by a physical reference electrode in 3E systems. In contrast, conventional 2E systems can only provide the overall cell voltage, which is unable to directly detect metallic Li plating processes (Fig. 3a). Using our virtual reference electrode in our Gr||LFP 2E cell, we can continuously monitor the NEP during charging (Fig. 3b–e) without additional sensing equipment, and perform post-mortem analysis of the graphite surface at various charging states (3 duplicate cells for each charging state). At a predicted NEP just above (i.e., 0.01 V, shown in Fig. 3b) and at the Li plating threshold (i.e., 0 V, shown in Fig. 3c), our scanning electron microscopy (SEM) experiments & optical images show that the graphite surfaces are smooth and similar to pristine graphite, indicating that Li metal plating has not occurred (Fig. 3f, g and Supplementary Fig. 15a, e, i and b, f, j). However, when predicted NEP is just below the Li plating threshold (i.e., −0.01 V, shown in Fig. 3d), shiny Li particles are captured on graphite surfaces via SEM (Fig. 3h and Supplementary Fig. 15c, g, k), indicating the ongoing Li nucleation stage. At later stages of charging where our virtual reference predicted a more negative NEP value (−0.02 V, shown in Fig. 3e), we observed filamentary and mossy structures on the graphite surfaces via SEM, which are direct markers of Li metal plating (Fig. 3i and Supplementary Fig. 15d, h, l), even if the plating is not visible in the optical image (Supplementary Fig. 10, which demonstrates the acuity of our smart sensing approach). Furthermore, we quantitatively analyze the Li plating coverage across four predicted NEP states. Li coverage is nearly zero when the predicted NEP is 0.01 or 0 V, whereas it increases to >10% at a predicted NEP of −0.01 V and further to >50% at −0.02 V (see methods Supplementary Figs. 15, 16 and Table S6). Moreover, both pixel-area and greyscale analyses show a strong and statistically significant negative correlation between predicted NEP and Li coverage (Table S7), confirming that lower predicted NEP is associated with increased Li plating. Consistent with this trend, Li deposit size analysis further shows that the size of Li deposits increases as the predicted NEP decreases (Supplementary Fig. 19). Together, these results further support that the model-predicted NEP captures both the onset and the morphological progression of Li plating. Without additional sensing equipment in conventional 2E cells, such a lithium-plating failure mode would go undetected during operation and likely compromise the safety and stability of the battery. Instead, 2E cells equipped with our virtual reference electrode can detect the onset of Li metal plating similar to 3E cells with a physical reference. This capability to continuously monitor and avoid failure without physical sensing equipment is significant, thus making it possible to intelligently optimize battery performance without the need for any changes in battery manufacturing or chemistry.

### Implementation of ML model on cycle life improvement

**Fig. 4:**
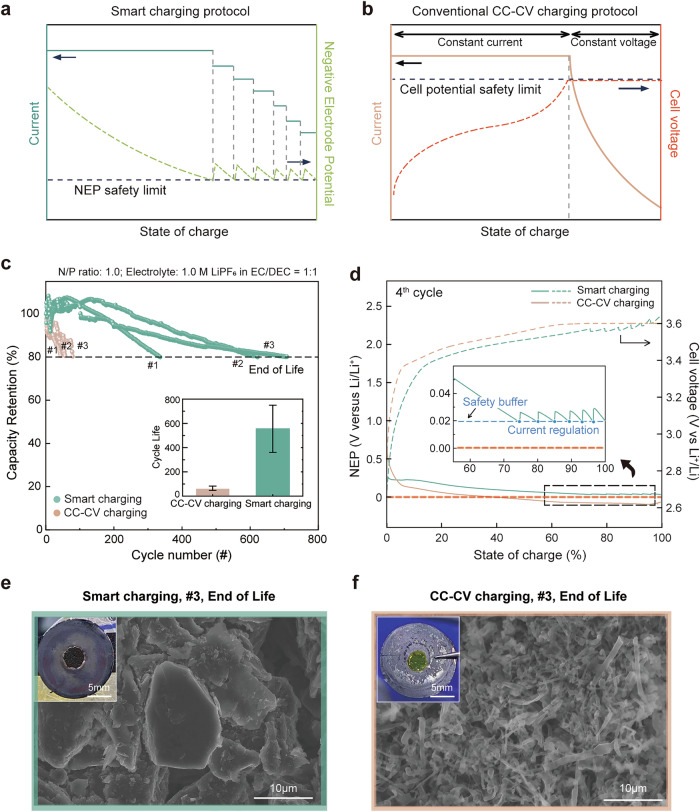
A smart charging protocol enabled by our virtual reference electrode. Conceptual profiles of voltage and current for **a** smart charging protocol and **b** CC-CV protocol. **c** Capacity retention of smart charging versus conventional constant-current constant-voltage charging. Capacity-retention curves represent individual cells. The inset shows the average cycle life for both protocols. Data in the inset are presented as mean values ± standard deviation from *n* = 3 independently cycled cells for each protocol. **d** Plots of measured NEP in CC-CV cell #3 (shown in solid brown line), predicted NEP in smart charging cell #3 (shown in solid green line), and cell voltage of both protocols (dashed brown line for CC-CV, and dashed green line for smart charging). SEM images & optical images of the top surface of graphite negative electrode at end-of-life for **e** smart charging cell #3, and **f** CC-CV charging cell #3. CC-CV, constant-current constant-voltage. All electrochemical tests were performed at 25 °C. Source data are provided as a Source Data file.

We design a smart charging protocol for 2E cells equipped with our virtual reference electrode (Fig. 4a), aiming to prevent Li metal plating by maintaining the predicted NEP above 0 V vs. Li metal. This would mitigate capacity degradation associated with Li metal reactivity and safety risks associated with short circuits, ultimately prolonging the cycling stability without requiring changes in battery chemistry or architecture. Our smart BMS starts charging at a constant current density of 2.37 mA cm^−2^ that dynamically decreases by increments of 0.237 mA cm^−2^ each time the real-time, predicted NEP reaches 0.02 V vs. Li metal. Charging continues until the last constant current step falls to 0.948 mA cm^−2^. We set the safety threshold at 0.02 V as a conservative engineering margin, informed by model uncertainty and near-threshold false-negative analysis, to reduce missed plating-risk events and more conservatively target plating-free charging (details are provided in Supplementary Note #3). For comparison, we cycle a 3E cell equipped with a physical reference electrode, using a conventional 2E CC-CV charging protocol (detailed parameters are given in SI) with the same discharging conditions and battery chemistry as our smart charging protocol (Fig. 4b). This physical reference electrode allows us to measure the NEP profiles during conventional CC-CV charging and compare them with the predicted NEP profiles of our 2E smart BMS cells. Since low N/P ratios often compromise battery safety and stability to enable higher energy densities, we use a low N/P ratio (~1.0) Gr||LFP battery chemistry (2.37 mAh cm^−2^ areal capacity) as a challenging stress test for our smart BMS to effectively prevent Li metal plating and extend cycle life. Remarkably, our smart BMS enabled an 8.25x enhancement in the cycle life of Gr||LFP compared to a conventional CC-CV baseline (Fig. 4c) with comparable or even higher average charging speed (Supplementary Fig. S7). Post-mortem analysis reveals that this performance enhancement is a direct result of mitigating Li metal plating (Fig. 4e, f). Whereas metallic Li is clearly observed both optically and with SEM in fully discharged CC-CV cells at end-of-life (80% capacity retention) (Fig. 4f), the surface of the graphite negative electrode remains smooth and pristine in smart BMS cells after smart charging (Fig. 4e and Supplementary Fig. 11). At the same time, a 3E cell is used throughout the cycle life of the smart charging protocol to verify the accuracy of this method in predicting the negative electrode potential over the entire life cycle. The high and consistent accuracy between measured and predicted NEP verified the robustness of our model while dealing with long-cycled batteries and unseen charging C-rates (Supplementary Fig. 12). Interestingly, Li metal plating in our CC-CV cell occurs at an early 4^th^ cycle, during which the measured NEP drops below 0 V vs. Li metal (Fig. 4d). This result highlights the challenge with cycling low N/P ratio cells that are prone to Li metal plating, as both the current and cutoff voltage for our CC-CV protocol are relatively conservative and would not be expected to trigger metallic Li plating. This failure mode would have remained silent and undetected with conventional measurables like overall cell voltage and capacity decay, which largely appear to be nominal within the first 30 cycles of CC-CV. Without a reliable reference (physical or virtual), even advanced BMS remain blind to such safety hazards and must rely on empirical methods to optimize charging protocols. With our preliminary smart BMS charging protocol, we have shown an increase in cycle retention of low-N/P-ratio cells without any changes in cell chemistry or architecture. Although the present study demonstrates generalization to unseen stepped, dynamically controlled charging profiles, and different cutoff voltage domains (Table S8), a comprehensive mapping of model performance across broader BMS-relevant operating conditions, including temperature, and rest duration, remains an important direction for future investigation. Future work may also integrate process-control optimization with our virtual reference electrode to further improve battery performance.

## Discussion

In summary, we have developed an ML-powered virtual reference electrode that can accurately and continuously monitor NEP without a physical reference. Our deep learning models for NEP prediction were trained on a large 3E cycling dataset, which is a large experimentally measured three-electrode cycling dataset and supports this data-driven virtual sensing approach. NEP provides a directly interpretable control parameter for smart BMS to optimize charging protocols while preventing failure modes, addressing a limitation of conventional BMS. With our smart BMS enabled by our virtual reference, low NP ratio 2E cells consistently avoid Li metal plating to prolong their cycle life by 8.25-fold compared to the baseline without any change to battery chemistry or architecture. More broadly, beyond NEP, other parameters that are physically interpretable but difficult to measure (e.g., pressure) can be predicted using the same approach described in this work. Such ML-powered “virtual sensors” may establish a framework of “personalized battery health”, where individualized BMS can be tailored based on virtual sensing of dynamic, physically meaningful parameters (e.g., NEP, pressure) without the need to directly measure them.

## Methods

### Cell assembly

Fifty-five graphite/LFP 3-electrode cells with a nominal areal capacity of 2.37 mAh cm^−2^ were assembled to establish the dataset in this work. Each cell included commercial LFP (Hunan Yuneng Co., Ltd., consisting of 95% active material. Mass loading: 16 mg cm^−2^. Areal capacity: 2.37 mAh cm^−2^), graphite (MTI Corp. It consists of 93.2% active material. Mass loading: 5.8 mg cm^−2^. Areal capacity: 1.78 mAh cm^−2^). CR2032 3E coin cell casings (Arbin Instrument Inc.) are used. During assembly of 3E cells, 2 mm Li (Canrd) disc is installed at the center of 3E casings as the reference electrode. As shown in Fig. S1, LFP “donut” with an outer and inner diameter of 14 mm and 5 mm, respectively, is punched to avoid short circuit caused by direct contact between the reference electrode and the positive electrode. A similar “donut” structure on graphite negative electrode (outer diameter: 16 mm; inner diameter: 3 mm) is applied to achieve a NP ratio of 1.0, which is lower than the safety recommendation^47^ (1.1-1.2) and allows extensive data generation of negative electrode potential near the Li plating threshold. Calculation of *N*/*P* ratio is performed here:

1 $$\frac{N}{P}\,=\,\frac{1.78\frac{{mAh}}{{{cm}}^{2}}*{pi}*[{\left(1.6{cm}\right)}^{2}-{\left(0.4{cm}\right)}^{2}]}{2.37\frac{{mAh}}{{{cm}}^{2}}*{pi}*[{\left(1.4{cm}\right)}^{2}-{\left(0.5{cm}\right)}^{2}]}=1.0$$

A 4-mm-diameter Kapton tape disc was placed on the central opening of the graphite electrode to avoid Li plating on the spacer due to non-uniform Li⁺ flux^48^. Then, 70 µl of standard electrolyte (1 M LiPF₆ in 1:1 v/v EC/DEC, Sigma-Aldrich) was added to each cell. Each cell used one 25 µm thick trilayer polypropylene–polyethylene–polypropylene separator (Celgard 2325), which was punched into a 16 mm diameter circular disc and placed between the two electrodes. The separator has a calculated porosity of 39% and a reported average pore size of 0.028 µm. For assembly of 2E cells, the setup was identical to that of 3E cells, except that the reference electrode was not included.

### Cycling and data collection

All cells were tested in an Arbin MSTAT21044 battery cycler at a constant temperature of 25 °C. Various candidate constant current-constant voltage (CC-CV) are applied to generate a diverse 3E-dataset after 3 formation cycles with 0.24 mA cm^−2^ (C/10). In constant-CC-charging cells, a single current density was applied to charge the cell to 3.6 V with a current cutoff of 0.12 mA cm^−2^ (C/20), while in stepped-CC-charging cells, multiple current densities were applied in succession and C-rate is switched whenever the measured negative electrode potential hit the threshold we set. Details of protocols for each cell for model training are given in Table S1. For baseline CC-CV protocol applied in Fig. 4 in the main text, 2.23 mA cm^−2^ was applied to charge the cell to 3.6 V with a current cutoff of 0.12 mA cm^−2^ (C/20) to make the average charging rate comparable to smart charging at early cycles (see Supplementary Fig. 7). All cells were subsequently discharged with a CC-CV discharge at 1.18 mA cm^−2^ (C/2) to 2.0 V with the same cutoff current of 0.12 mA cm^−2^.

### Machine learning model development

#### Data processing and feature engineering

Data from the first 100 cycles was used for training and testing the models. Cycles that were less than the end of life (defined as 80% capacity retention^49^) were also removed from the dataset. It was expected that the CC portion of the cycling protocol above 3.3 V would likely promote lithium plating more than the CV portion (and CV would also be avoided during smart BMS charging), so CC data below 3.3 V and the CV data were subsequently removed. Afterward, the negative electrode potential measurement was smoothed using a rolling average of a fixed window size, in order to reduce the impact of measurement noise and enable precise diagnosis of the battery^50^.

A 78-22 train-test split is achieved by splitting the data by the number of measurements made, which are recorded every second. The protocols are denoted in Table S1 and the exact cells used in the split are denoted in Supplementary Table S2.

Engineered features are all calculated by basic measurements that can be obtained by the BMS, such as current, capacity, etc. Overpotential is calculated by taking the difference between the presently measured voltage and the corresponding voltage, denoted as *V*_ref_, from the last formation cycle as a function of state of charge (SOC):

2 $$\,\eta={V}_{{{\mathrm{cell}}}}({{\mathrm{SOC}}})-{V}_{{{\mathrm{ref}}}}({{\mathrm{SOC}}})$$

The last formation charge cycle is sufficiently slow such that it approximates the thermodynamic OCV curve. To find SOC, the measured capacity is first divided by the nominal capacity to obtain a state of charge (SOC) value between 0 to 100. Then, a cubic interpolation is applied on the SOC vs. *V*_ref_ curve to obtain a reference voltage measurement for all SOC. During cycling, the measured capacity is converted to an SOC value using the nominal capacity, which can then be used to directly find the difference between corresponding values of *V*_cell_ and *V*_ref_. Derivative features such as d(Cell Voltage)/dt and dEnergy/dt were found by taking the gradient of the corresponding feature. The lagged features are obtained by recording basic features and calculating engineered features on 2, 4, 6, and 8 s prior to the present measurement. A relatively short timeframe was used since dynamic charging would repeatedly change the current and other features. It was expected that long historical information, which may be relevant in our protocols used for model development, may not translate to the dynamic protocol.

### Feature selection

In our feature selection process, Recursive Feature Elimination (RFE) is employed using a Random Forest Regressor as the base estimator. The RFE method recursively removes the least important features, based on the importance weights assigned by the regressor. The Random Forest Regressor makes the most sense as the base estimator since importance weights are an implicit calculation made in the model. In the model, for each decision tree in the forest, at each node, a split based on a particular feature is decided based on how much that split reduces the “impurity.” For regression, this is typically the MSE or mean-squared error. Once a decision tree has been trained, the total reduction in impurity (MSE) contributed by each particular feature is aggregated. This aggregated “impurity reduction” is averaged over all decision trees in the random forest and given as the “importance” for each particular feature. In summary, features that are given high “importance” led to a higher reduction in “impurity” or MSE in the decision trees, logically meaning that they contribute more to the final prediction.

The process begins with all possible electrochemical domain features (Table S3) and iteratively eliminates 15 features per step until only the top 5 most significant features remain. Throughout this process, the Random Forest Regressor, configured with 30 decision trees and utilizing parallel processing for efficiency, ranks the features according to their predictive power. Once the RFE has identified the most important features, a new Random Forest Regressor is trained exclusively on these selected features to further assess and quantify their importance (see Fig. 2 in the main text). This method can reduce the dimensionality and overfitting of the dataset and enhance the interpretability and performance of predictive models by focusing on the most influential features.

### Model training

#### Ensemble neural network models

An ensemble of five feedforward neural networks is designed using Tensorflow. Ensemble models were chosen to reduce overfitting and boost the performance of weak individual learners. Each neural network is designed with an input layer that matches the number of input features, followed by three hidden layers: the first with five neurons using the ReLU activation function, the second and the third with 256 neurons each, using ReLU and leaky ReLU activation functions, respectively. The output layer has a single neuron with a linear activation function. Linear layers are chosen for this regression task to allow the dense connections to learn the complex relationships between the different input features. The models are trained with a learning rate of 0.001, batch size of 128, and for 70 epochs. To reduce variance and overfitting, as well as enhance robustness, 70% of the training data is randomly sampled with replacement for each model as bootstrapping. A dropout of 20% was also applied to hidden layers to help prevent overfitting. Five models are trained independently, and the predictions of the five models are then averaged to improve overall accuracy and reduce uncertainty. Early stopping is not employed, but L1 regularization on the model parameters with a weight of 1e−4 is used. Relative learning curves and variance across random seeds are provided in Supplementary Figs. 17 and 18, respectively. To fairly compare ML results with physics-based model (PBM), once the ML model is trained, it is deployed on new cells without any per-cell calibration, retraining, or fine-tuning.

After model training, root-mean-square-error (RMSE) and mean-absolute-error (MAE) are chosen to evaluate model performance. RMSE is defined as:

3 $${{{\rm{RMSE}}}}=\,\sqrt{\frac{1}{n}{\sum }_{i=1}^{n}{({y}_{i,p}-{y}_{i,m})}^{2}}$$

Where *y*_*i,p*_ is the predicted negative electrode potential, *y*_*i,m*_ is the measured negative electrode potential and n is the total number of samples. MAE is defined as:

4 $${{{\rm{MAE}}}}=\,\frac{{\sum }_{i=1}^{n}\left|{y}_{i,p}-{y}_{i,m}\right|}{n}$$

where all variables are defined as above.

Although low-voltage data (<3.3 V) and constant-voltage (CV) stage data were not included in the primary training dataset used in the main text, to demonstrate the robustness of our approach, an ensemble neural network was additionally trained using the full voltage domain (2.0–3.6 V), including CV-stage data, and performance is shown in Supplementary Fig. 14.

### Hyperparameter tuning

Model hyperparameters were chosen using a hyperparameter tuning process. Specifically, hidden unit size, number of hidden layers, learning rate, dropout rate, batch size, and number of ensemble models were all varied to identify the best possible model construction. We considered hidden units between 32 and 544, evenly spaced by 64, 1–4 hidden layers, learning rates of 0.1, 0.01, 0.001, dropout rates of 0.2, 0.3, and 0.5, batch sizes of 32, 64, 128, and 256, and 1–5 models for ensemble size. For hyperparameter tuning, the training set was further split (80/20) into a smaller training set and a validation set used to pick between hyperparameters. Hyperparameter tuning was done using the Keras Tuner framework, using RMSE as the “validation metric” used to judge various models against each other.

### Gaussian processing regression (GPR), linear regression model, and tree-based model

The GPR model is trained with gpflow. A Matern 5/2 kernel is primarily used, which is a generalization of the squared exponential kernel and can capture non-monotonic smooth trends. A constant kernel is added to the kernel for a non-zero mean, while white noise is added to account for noise in the observations. GPR models are known to suffer from scalability, so a Sparse Variational Gaussian Process^51^ (SVGP) model is used with 800 inducing points. The number of training data points is also reduced by a factor of 15 to achieve reasonable training times. The Adam optimizer is used to minimize the model’s training loss, and the learning rate is set to 0.001. The maximum number of iterations for the optimization process is set to be 100.

The linear regression model is also trained as a baseline comparison via the LinearRegression function in sklearn.

The tree-based model performs regression through a series of binary choices. We set the maximum depth of our tree to be 3 layers deep and set the minimum number of samples in a leaf to 100 due to the large dataset size. We additionally used an ensemble of 5 trees, as the training process can return widely different trees from the same dataset. The model was trained using scikit-learn in a regression manner.

### Smart charging implementation

Smart charging with real-time NEP prediction and current regulation is enabled by a Console TCP Interface (CTI) to the Arbin battery management system (BMS). A program written in C# interacts with the BMS to collect real-time measurements of relevant features. The program then uses the features to call the trained models written in Python and obtain an NEP prediction. The current is subsequently adjusted according to the predicted value and selected threshold, leading to the response shown in Fig. 4a. The end-to-end closed-loop latency of the smart-charging system, including data sampling, communication via the Arbin CTI interface, ensemble model inference, and current update, is approximately 115 ms under the experimental configuration used. In the experimental implementation, the ML-based current regulation was constrained within predefined cycler-level voltage and current limits, and current updates were only applied within the safe operating window of the charging protocol. If delayed communication, abnormal input values, model execution failure, or abrupt nonphysical prediction behavior occur, the control logic reverts from ML-based current adjustment to a predefined conservative CC-CV charging protocol.

### Physics-based simulation of the negative electrode potential

The physics-based model (PBM) used in the present study is a pseudo-two-dimensional (P2D) model that has been developed and documented by Zhu et al.^52^. However, the model is tailored to the physical characteristics and materials that represent the present coin cells. Where available, material properties are taken from published literature. The open-circuit potential for the Li_x_FePO_4_ positive electrode is taken from Safari and Delacourt^53^, and the open-circuit potential for the graphite negative electrode is based on Verbrugge et al.^54^. The ion conductivity, diffusion coefficient, lithium-ion transference number, and the thermodynamic enhancement factor for the electrolyte LiPF_6_ in EC:DEC (1:1 w:w) are based on measurements by Lundgren et al.^55^. Once these properties and functions are fixed, other parameters within the model are adjusted to fit measured transient voltage-current behaviors. Measured voltage and current are available at intervals of Δ*t* = 1 s, over the entire charge-discharge experiments for many cycles. The model is tuned to represent the measured cell voltage-current behavior. The major fitting parameters include the exchange current densities and symmetric factors in Butler–Volmer charge-transfer rate, as well as the nominal electrode particle size and lithium diffusion coefficient within the particles. For each cell, the PBM parameters were calibrated once using current-voltage data from an early reference cycle and were subsequently held fixed for all later-cycle predictions, without any cycle-by-cycle re-tuning.

### Post-mortem analysis

#### SEM sample preparation and imaging

After running the electrochemical experiments described in the main text, the cells were transferred to an Ar-purged glove box. After disassembling cells, graphite negative electrodes were gently rinsed with a few drops of anhydrous dioxolane and then affixed to an SEM sample stage using conductive tape after drying. The SEM stage with samples was placed in a Teflon box and tightly sealed with parafilm. The pressure in the glove box and the sealed box was greater than ambient pressure, which prevented air from leaking into the box. Once taken out of the glove box, the SEM stage was quickly transferred into the SEM chamber (approximately 5 s) to avoid air exposure. All SEM characterizations were conducted using ZEISS Supra 40VP SEM with a 10-kV acceleration voltage. Cell disassembly, electrode harvesting, rinsing, drying, and SEM sample-stage preparation were performed in an Ar-purged glove box at 25 °C.

### Li coverage (%) estimation from SEM images

Lithium coverage was quantified using two independent quantitative analysis approaches on ImageJ: pixel area analysis and grayscale analysis. For the pixel area analysis, lithium coverage was calculated by summing the pixel areas of all lithium-plating-related regions, including both nucleation sites and larger plating features, as identified in SEM images (highlighted in red in Fig. S15). Then the total lithium-plated pixel area was normalized by the total pixel area of the corresponding SEM image to obtain the lithium coverage fraction. For grayscale analysis, each SEM image was first converted to an 8-bit grayscale format (intensity range: 0–255), and the pixel density distribution across grayscale levels was obtained. Owing to variations in brightness and contrast among SEM images, an image-specific grayscale threshold was selected to represent lithium-plated regions. Lithium coverage was then quantified as the area fraction of pixels exceeding this threshold relative to the total image area. For each predicted NEP condition, three independently cycled cells were analyzed. SEM quantification was performed at the same representative region near the edge of the inner graphite ring for each cell, where Li plating is most readily observed, to improve comparability across cells and NEP conditions. For both methods, data are reported as the mean ± standard deviation across three independent cells. The 95% confidence interval (CI) of the mean Li coverage was calculated using the Student’s *t*-distribution:

5 $${{{{\rm{CI}}}}}_{95\%}\,=\,\bar{x}\pm \,{t}_{0.975,n-1}\frac{s}{\sqrt{n}}$$

where $\underline{x}$ is the mean Li coverage, s is the sample standard deviation, and n is the number of independent cell samples. In this study, n = 3 for each NEP condition, so ${t}_{{{\mathrm{0.975,2}}}}$ = 4.303. The resulting CI represents the uncertainty in the estimated mean Li coverage for each predicted NEP condition.

To quantitatively evaluate the association between predicted negative electrode potential and Li coverage, all individual cell-level sample data points across the four NEP conditions were included in the correlation analysis. For each Li-coverage quantification method, predicted NEP values were paired with the corresponding Li coverage values from individual cell samples. Spearman's rank correlation was used to assess the strength and direction of the monotonic relationship between predicted NEP and Li coverage, without assuming a linear relationship. Pearson correlation was additionally calculated to evaluate the strength of the linear relationship between predicted NEP and Li coverage. For both analyses, two-sided *p*-values were calculated under the null hypothesis of no correlation between predicted NEP and Li coverage. The *p* value represents the probability of observing a correlation at least as strong as the measured one if no true association exists; therefore, a smaller *p*-value indicates stronger statistical evidence against the null hypothesis. A negative correlation coefficient indicates that a more negative predicted NEP is associated with higher Li coverage. The correlation coefficients and corresponding *p*-values are summarized in Table S7. SEM imaging regions were not treated as additional independent replicates. Because the analysis includes four predicted NEP levels with three independent cells per level, the *p* values should be interpreted as supporting the observed monotonic trend rather than as a highly powered statistical test.

## Supplementary information

Supplementary Information

Peer Review File

## Source data

Source Data

## Data Availability

The raw battery cycling data generated and analyzed in this study are publicly available in the Zenodo repository under the dataset: 10.5281/zenodo.20937591^56^. Source data are provided with this paper.

## Code availability

The code used in this study is also released in a publicly available Zenodo repository at 10.5281/zenodo.21077954^57^.
