# Supplementary material for: Battery smart sensing via a virtual reference electrode
Source: Nat Commun. 2026 Aug 11;17:8336. doi: 10.1038/s41467-026-76535-y (PMC13473656; doi:10.1038/s41467-026-76535-y)
Supplement: Supplementary file 1 [file 41467_2026_76535_MOESM1_ESM.pdf]

Supplementary Information for

Battery smart sensing via a virtual reference electrode

Jiayi Yu*[1], Aki Takahashi*[2], Min-Ho Kim*[1], Rishi Upadhyay*[3], Howard Zhang*[3], Tian-Yu Wang*[1], Huayang Zhu[4], Robert Kee[4], Tyrone Vincent[5], Bo Liu[1], Xintong Yuan[1], Thomas Hymel[6], Kaiyan Liang[1], Keyue Liang[1], Haoyang Wu[1], Dingyi Zhao[1], Jung Tae Kim[1], **Achuta Kadambi[3]✉**, **Yuzhang Li[1]✉**

[1] Department of Chemical and Biomolecular Engineering, University of California, Los Angeles, Los Angeles, CA, USA

[2] Department of Mechanical and Aerospace Engineering, University of California, Los Angeles, Los Angeles, CA, USA

[3] Department of Electrical Engineering and Computer Science, University of California, Los Angeles, Los Angeles, CA, USA

[4] Department of Mechanical Engineering, Colorado School of Mines, Golden, CO, USA

[5] Department of Electrical Engineering, Colorado School of Mines, Golden, CO, USA

[6] Department of Materials Science and Engineering, Stanford University, Stanford, CA, USA

* These authors contributed equally to this work

✉ Corresponding author email: achuta.kadambi@gmail.com, yuzhangli@ucla.edu

Supplementary Figures

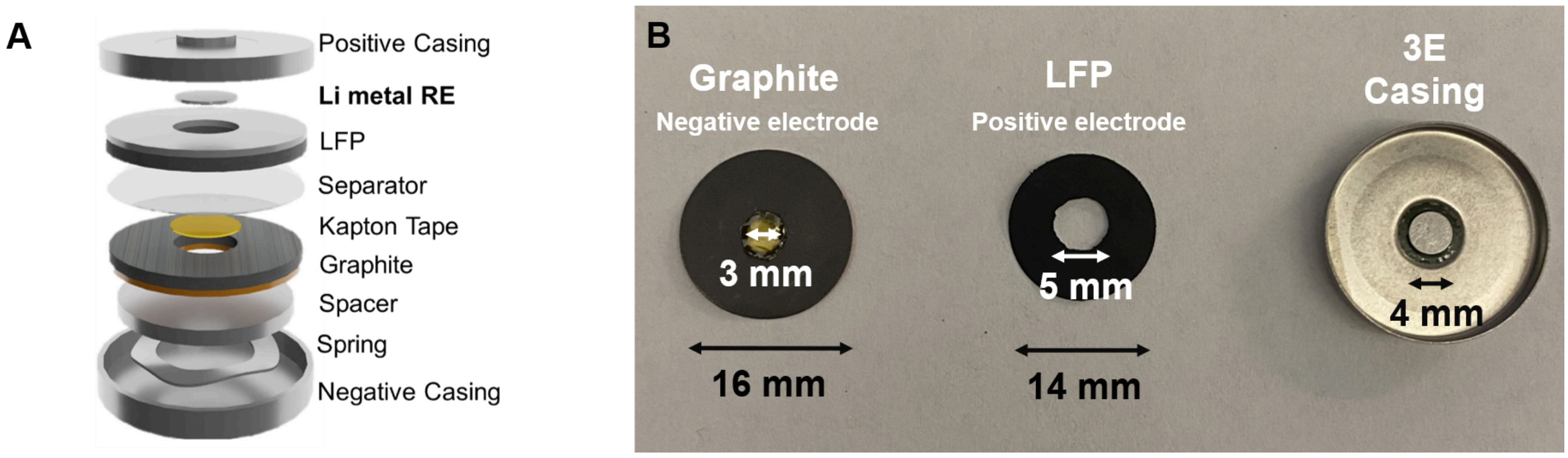

Figure S1. Experimental setup of 3E cell. a, Schematic of 3-electrode coin cell. **b,** Optical image of negative electrode, positive electrode, and a 3E casing.

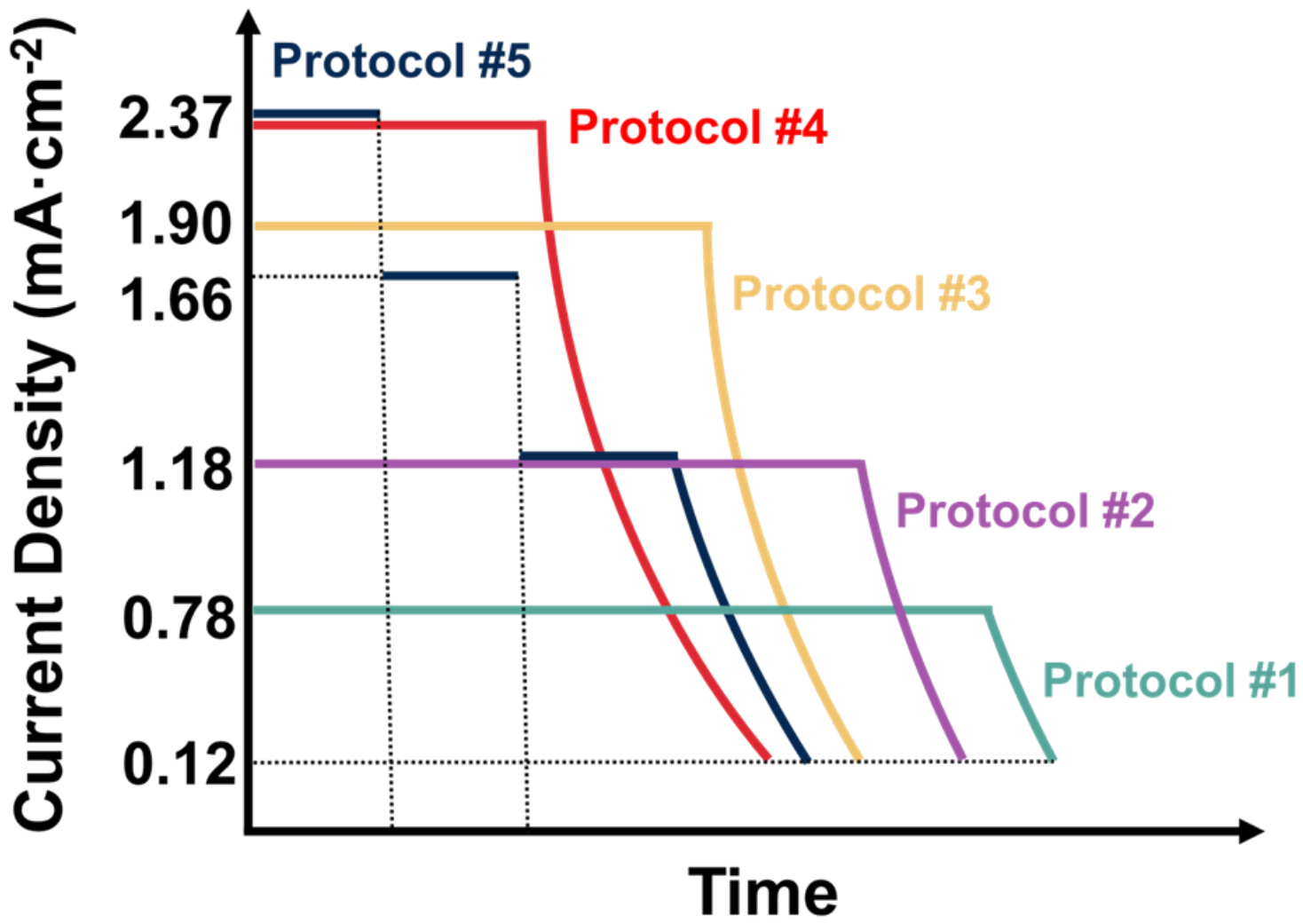

Figure S2. Charging protocols for 3E dataset. Protocol #1-#4 represent constant-CC-charging protocol. Protocol #5 represents stepped-CC-charging protocol. After CC charging, they are all followed by a CV charging until the current hits the cutoff value

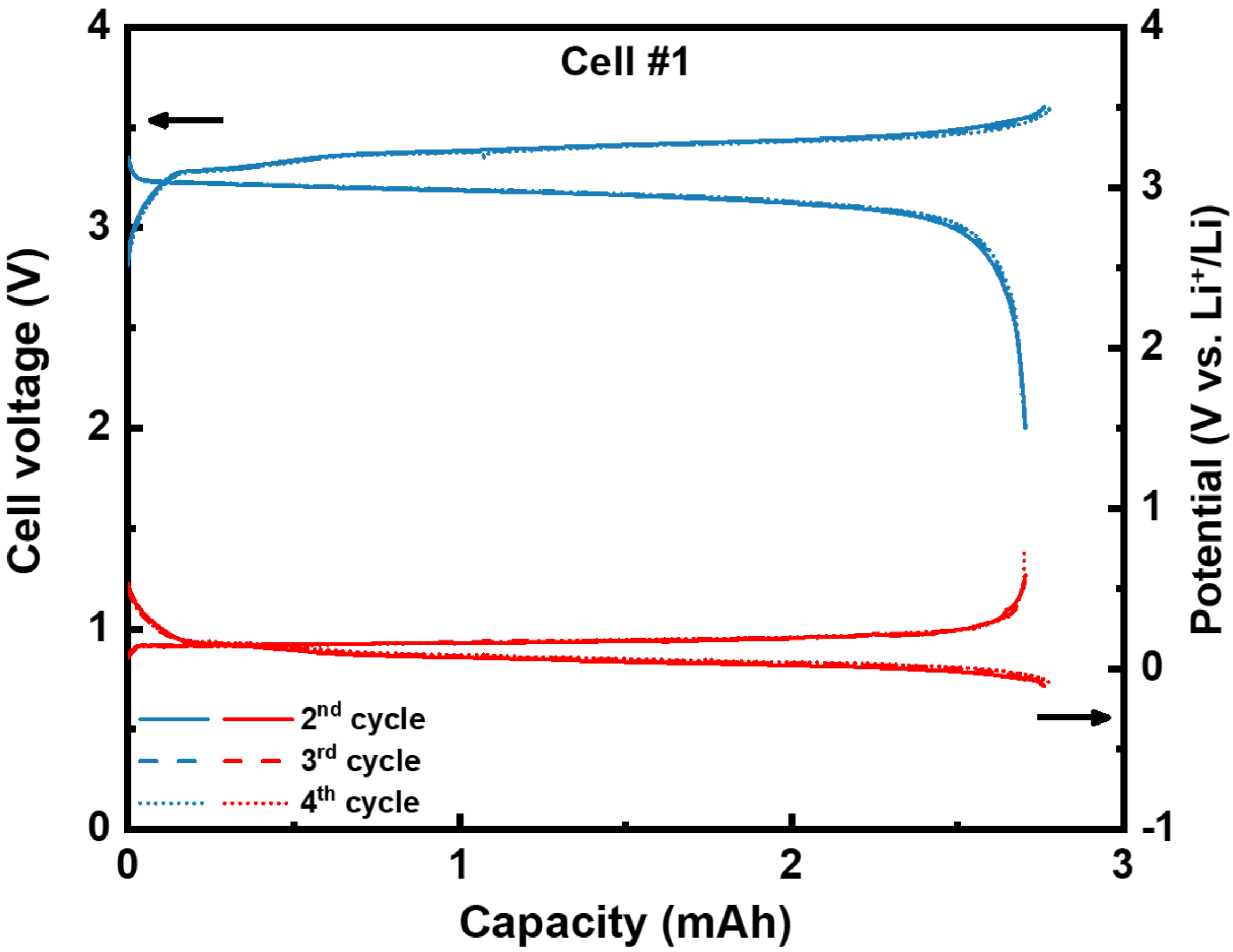

Figure S3. Cell voltage(left axis) and negative electrode potential(right axis) of Cell #1, from cycle 2 to cycle 4. It demonstrated the capability of 3E cell to simultaneously measure both parameters during cycling process. All electrochemical tests were performed at 25 °C. Source data are provided as a Source Data file.

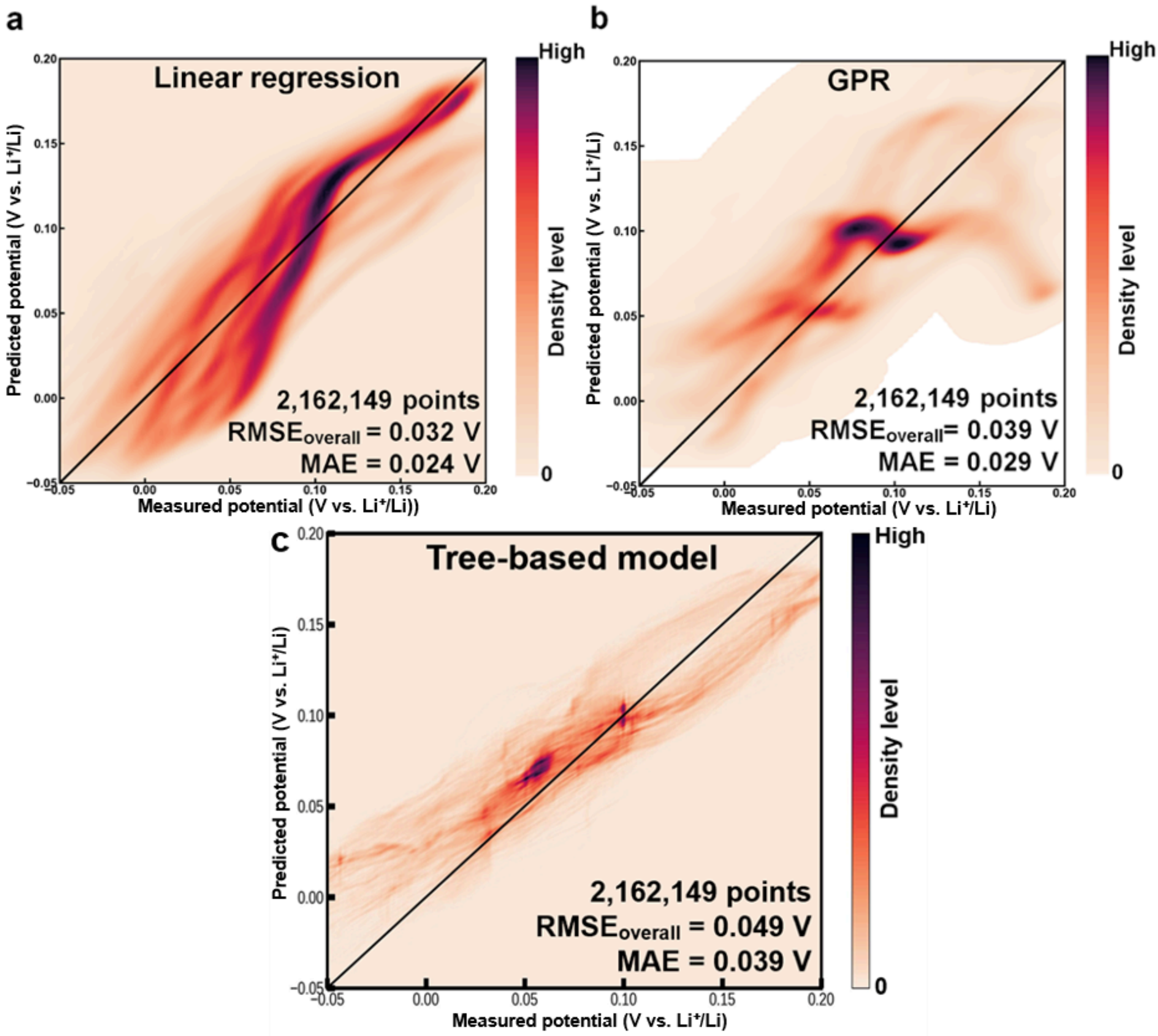

Figure S4. Performance of baseline models. 2D density plot of predicted NEP versus measured NEP in all cells in test for **a,** linear regression model; **b,** GPR model; **c**, Tree-based model

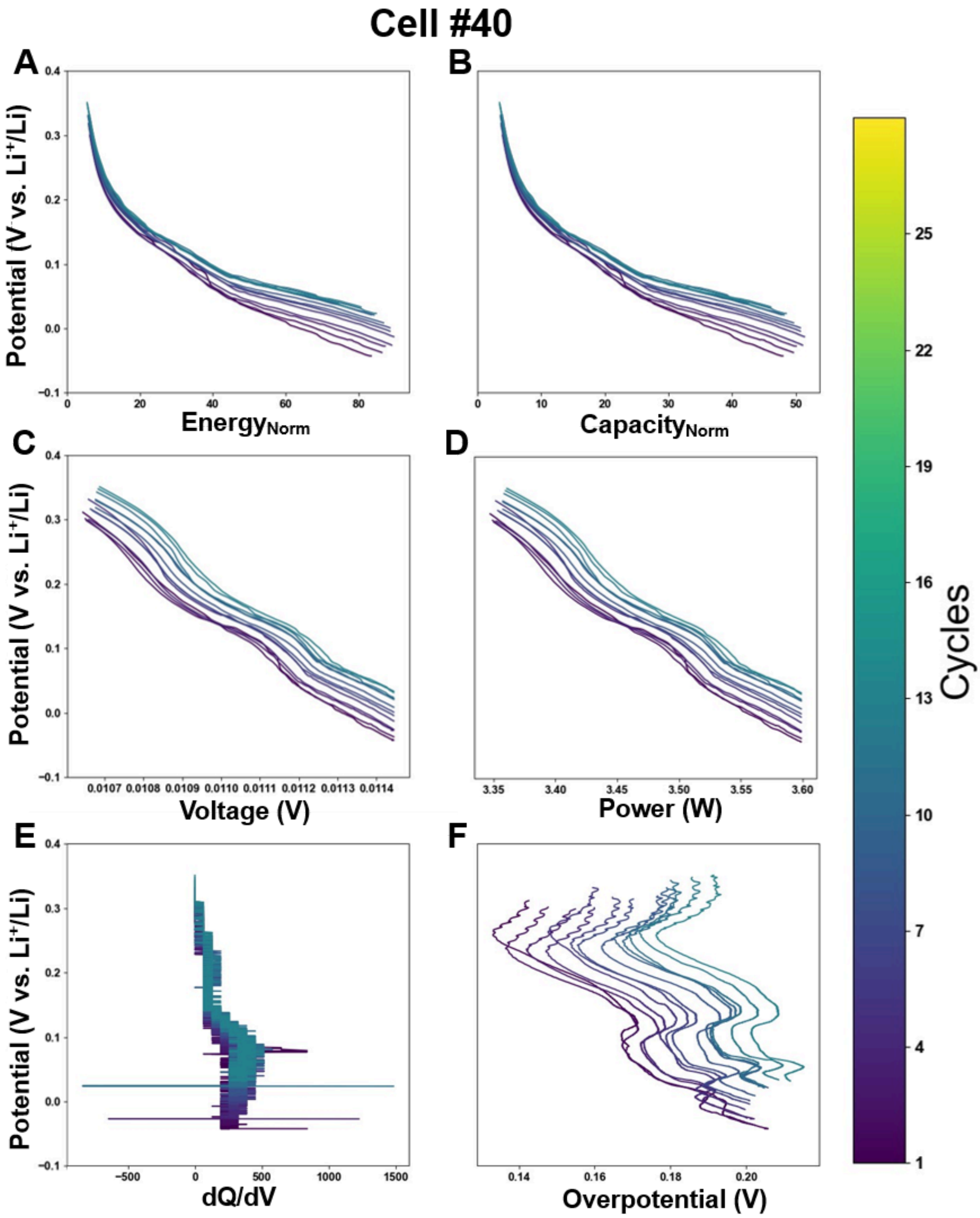

Figure S5. Negative electrode potential versus different features for a representative cell in 3E dataset. **a, b, c, d,** negative electrode potential versus selected features with high relative importance. **e, f,** negative electrode potential versus two of unselected features with low relative importance.

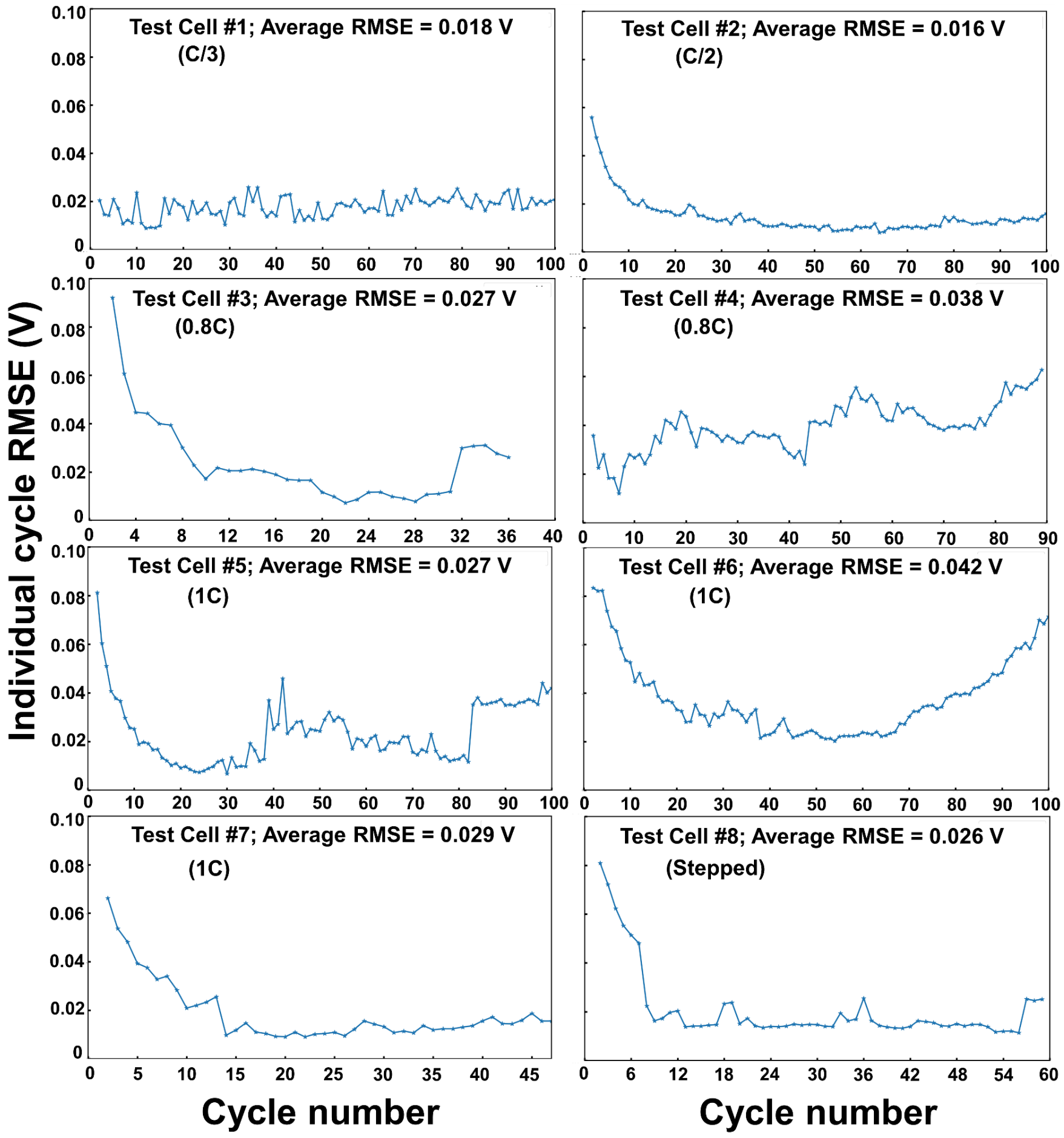

Figure S6. Error metrics of ensemble neural network for individual cycle of all 3E cells in the test set

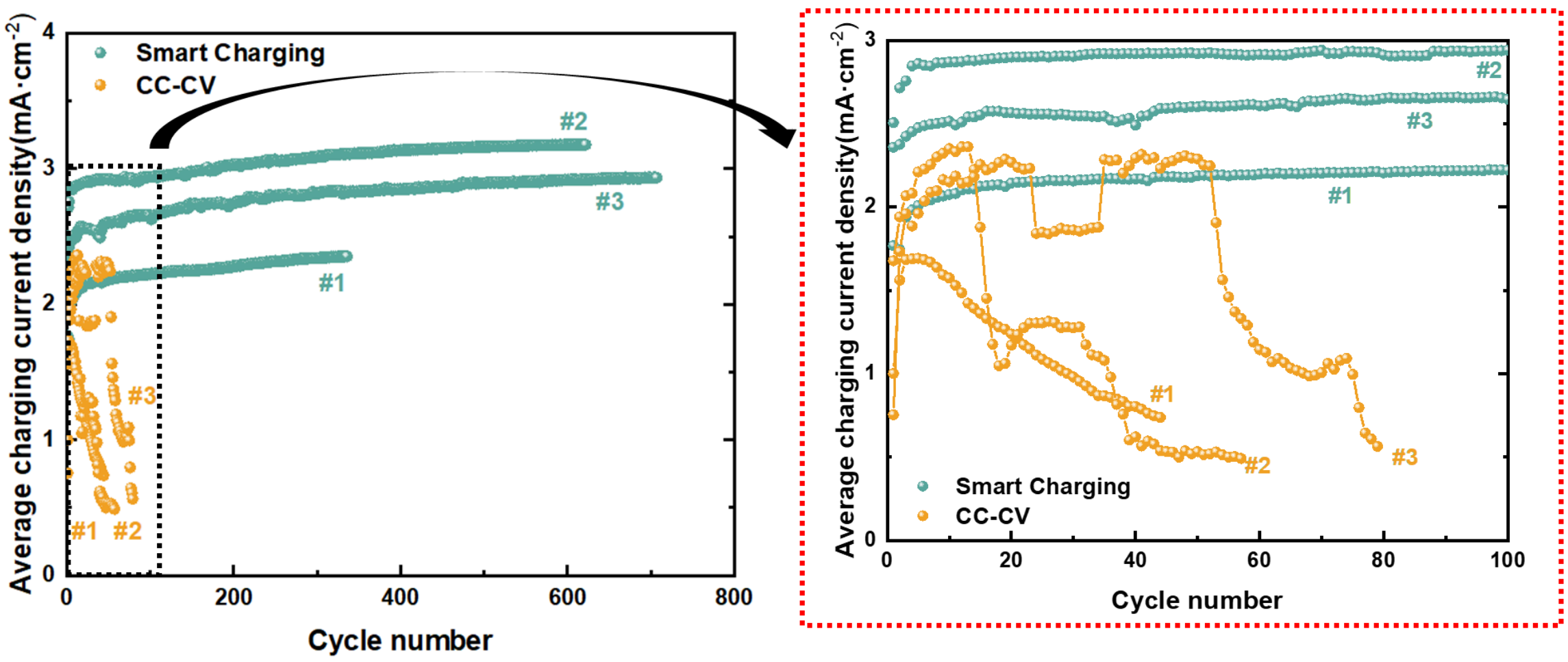

Figure S7. Average charging rate in current density for smart charging and CC-CV protocol. All electrochemical tests were performed at 25 °C. Source data are provided as a Source Data file.

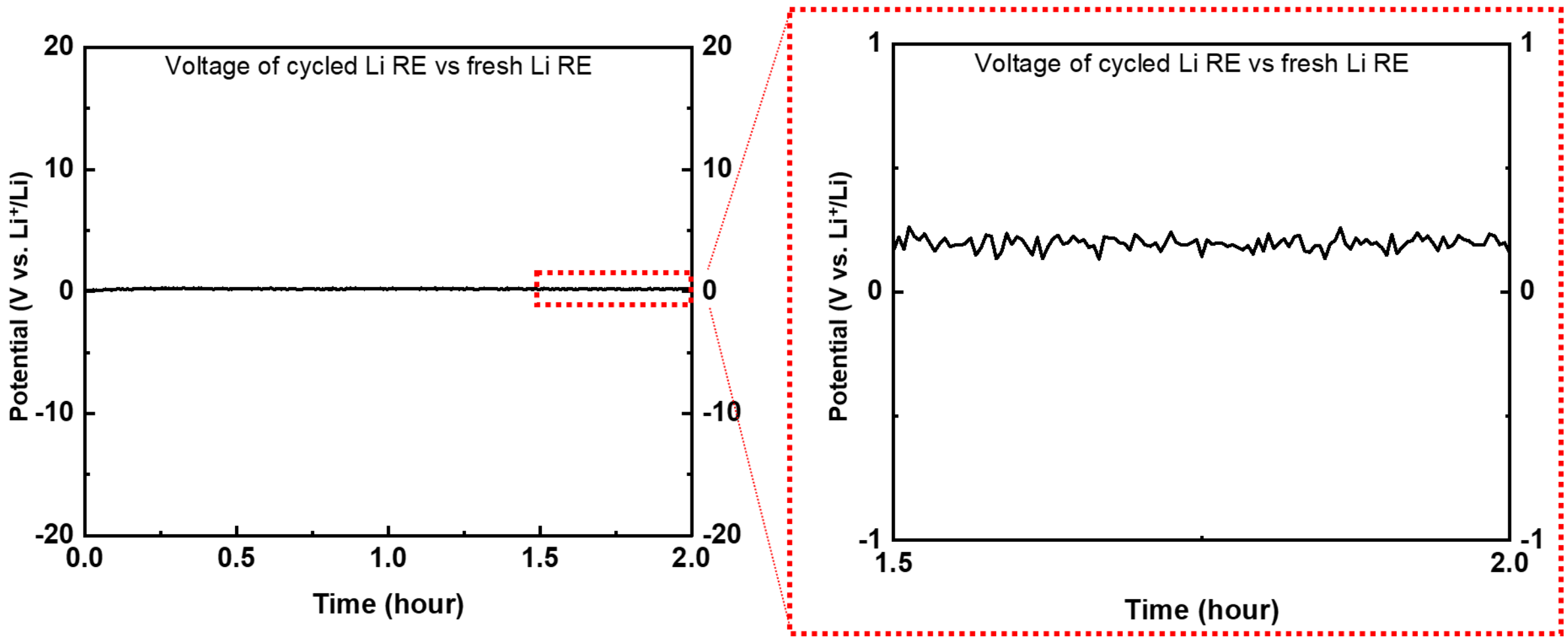

Figure S8. **Stability of Li metal RE, demonstrated by voltage difference between a cycled Li RE vs a fresh Li RE.** All electrochemical tests were performed at 25 °C. Source data are provided as a Source Data file.

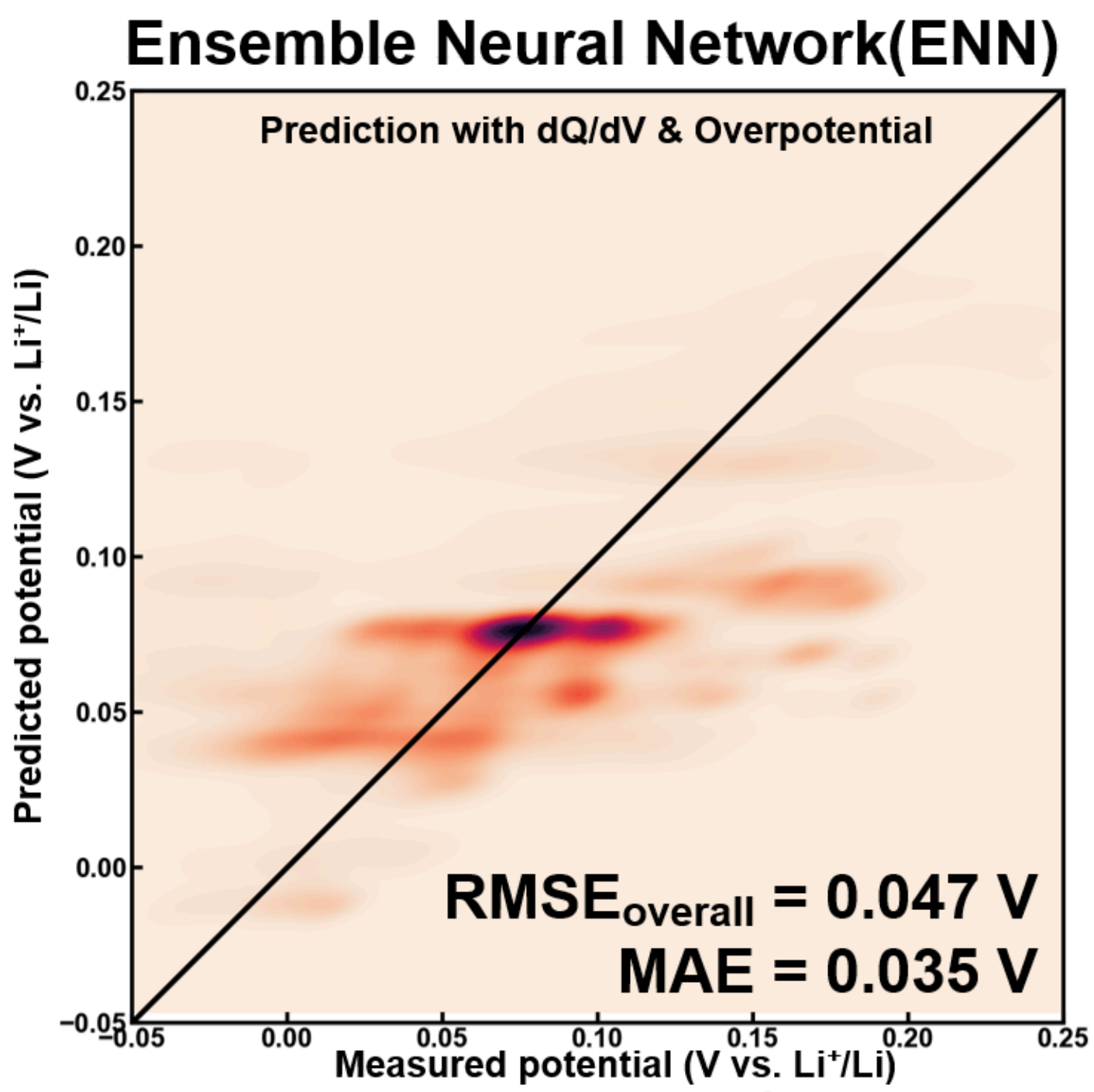

Figure S9. Performance of Ensemble neural network with dQ/dV & Overpotential as applied features on

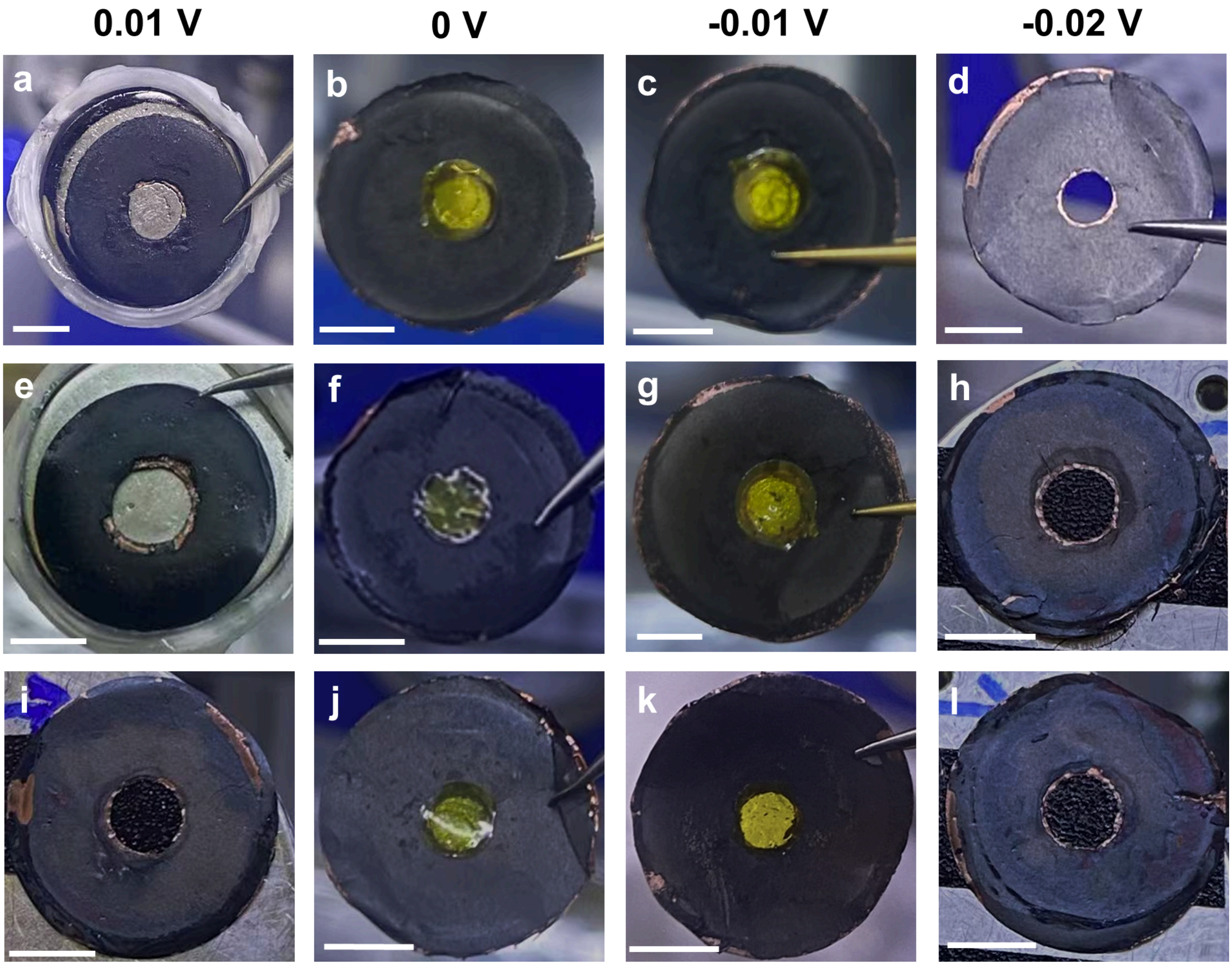

Figure S10. Optical image of graphite electrode for predicted NEP of (a,e,i) 0.01 V; **(b,f,j)** 0 V; **(c,g,k)** -0.01 V; **(d,h,l)** -0.02 V. Scale bar: 5 mm

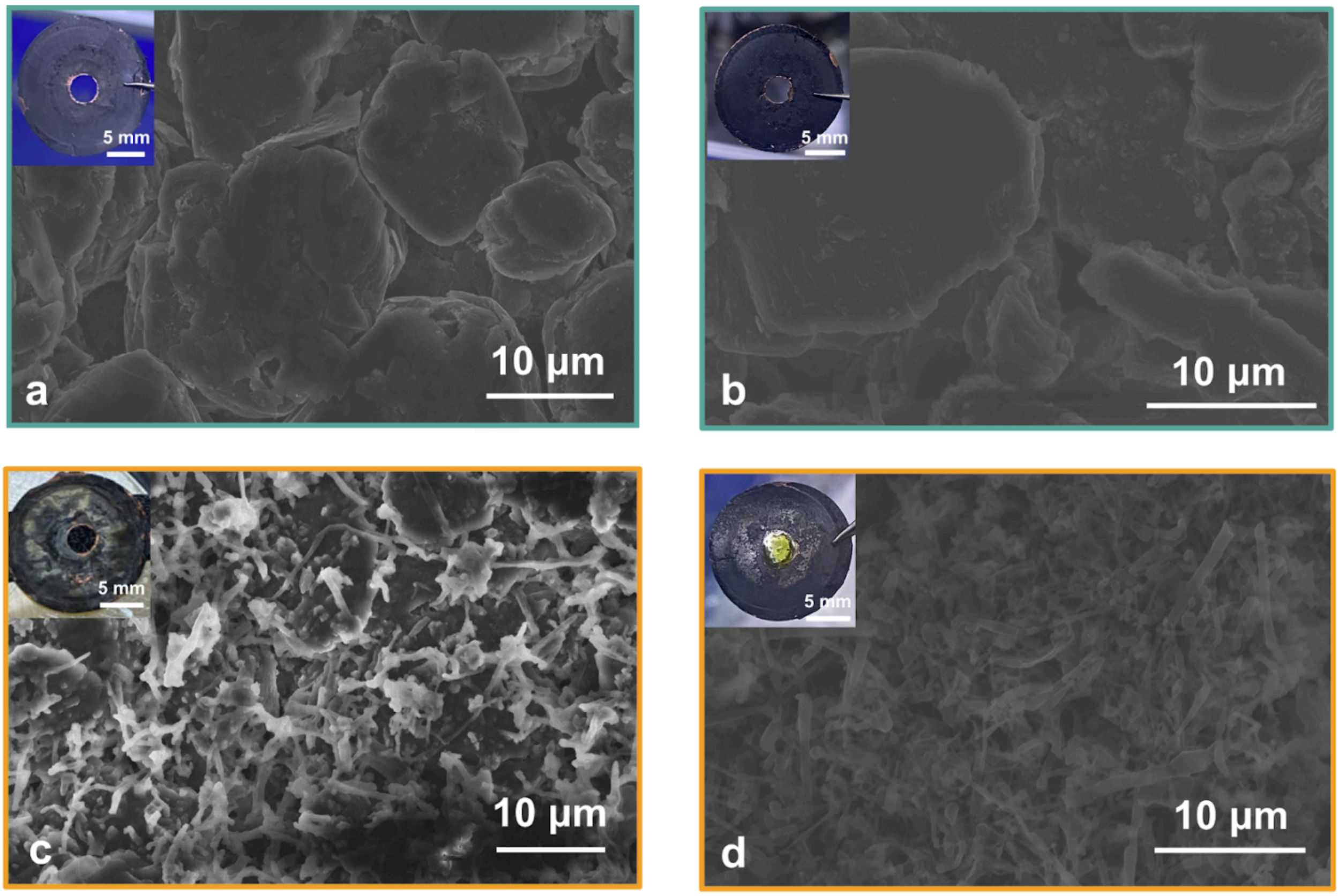

Figure S11. SEM images & optical images of the top surface of graphite negative electrode at end-of-life for (a) Smart charging cell #1; **(b)** Smart charging cell #2**; (c)** CC-CV charging cell #1**; (d)** CC-CV charging cell #2

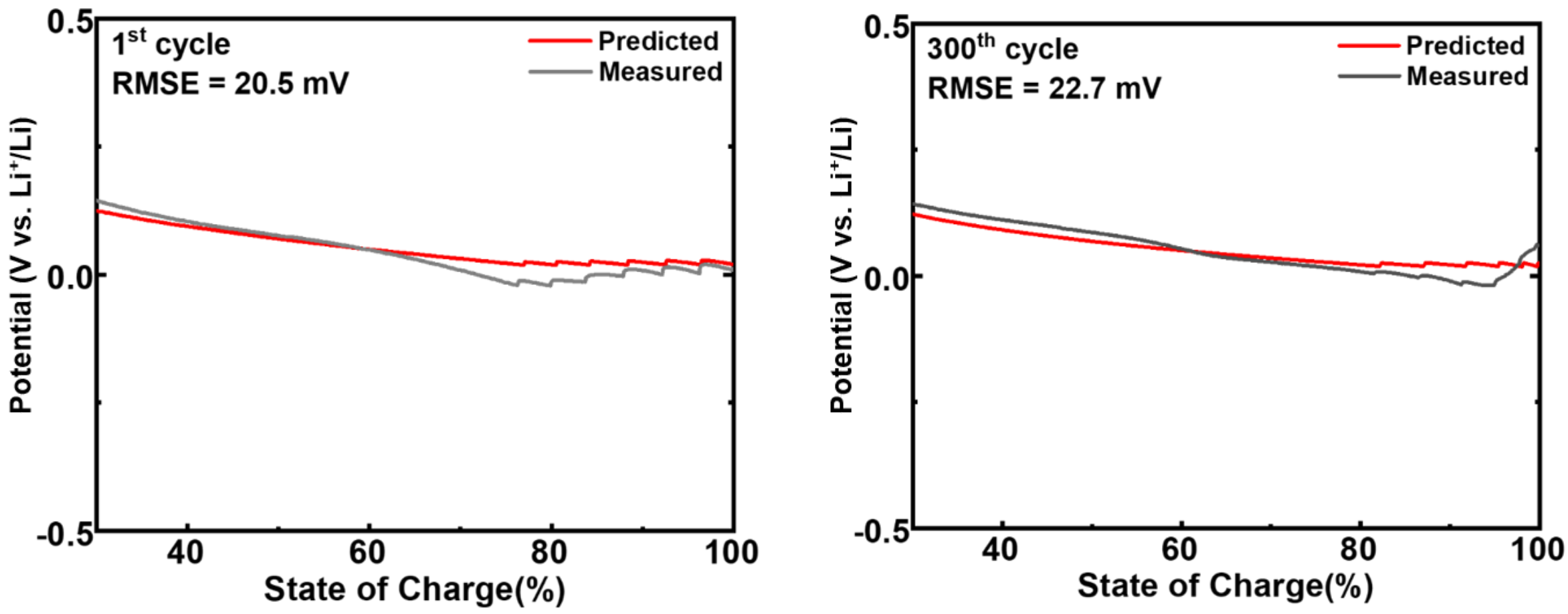

Figure S12. Predicted negative electrode potential vs Measured negative electrode potential throughout the life cycle of smart charging protocol, collected from a 3E cell. All electrochemical tests were performed at 25 °C. Source data are provided as a Source Data file.

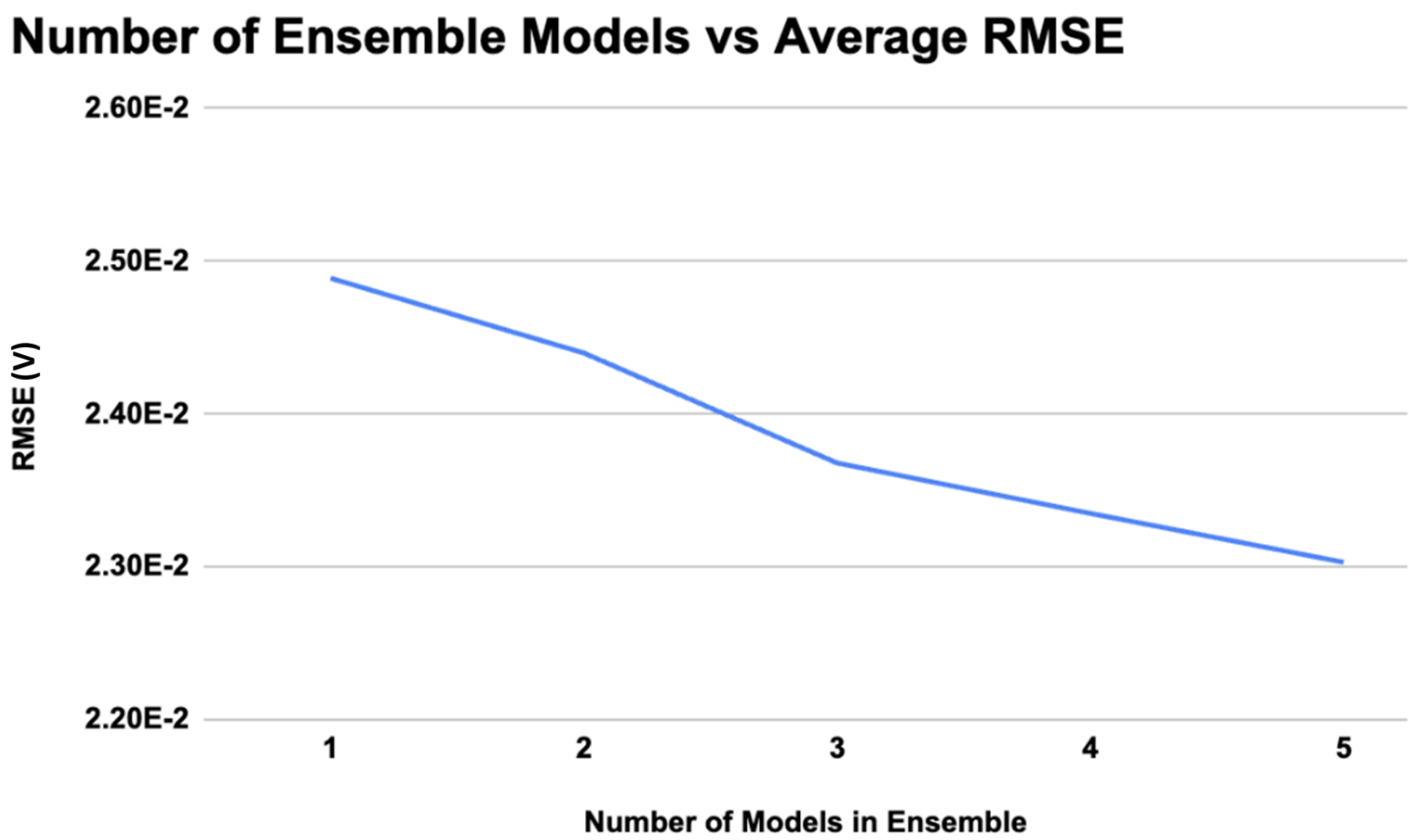

Figure S13. Number of models in the ensemble versus RMSE on the test set. The RMSE values represent the mean of five independent model-training runs performed using different random seeds. Source data are provided as a Source Data file.

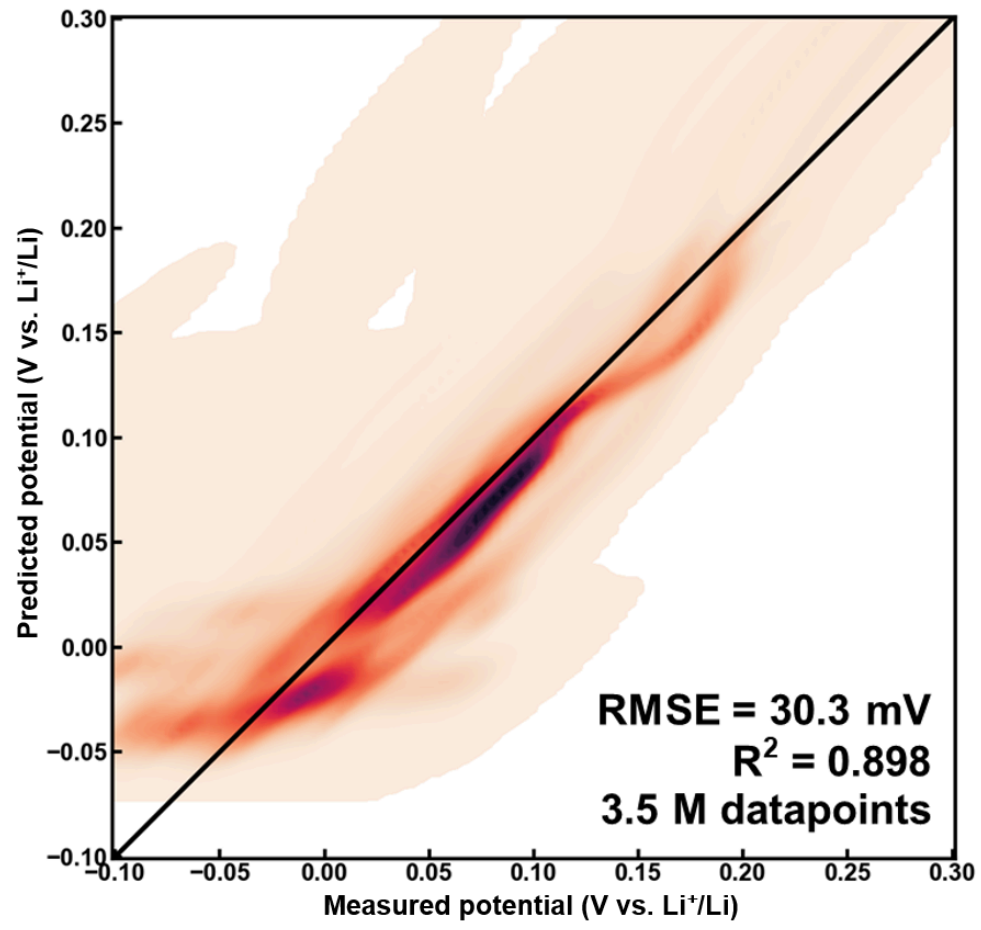

Figure S14. 2D density plot of predicted NEP versus measured NEP in all cells in test with full-voltage domain(2-3.6 V) and CV-stage included

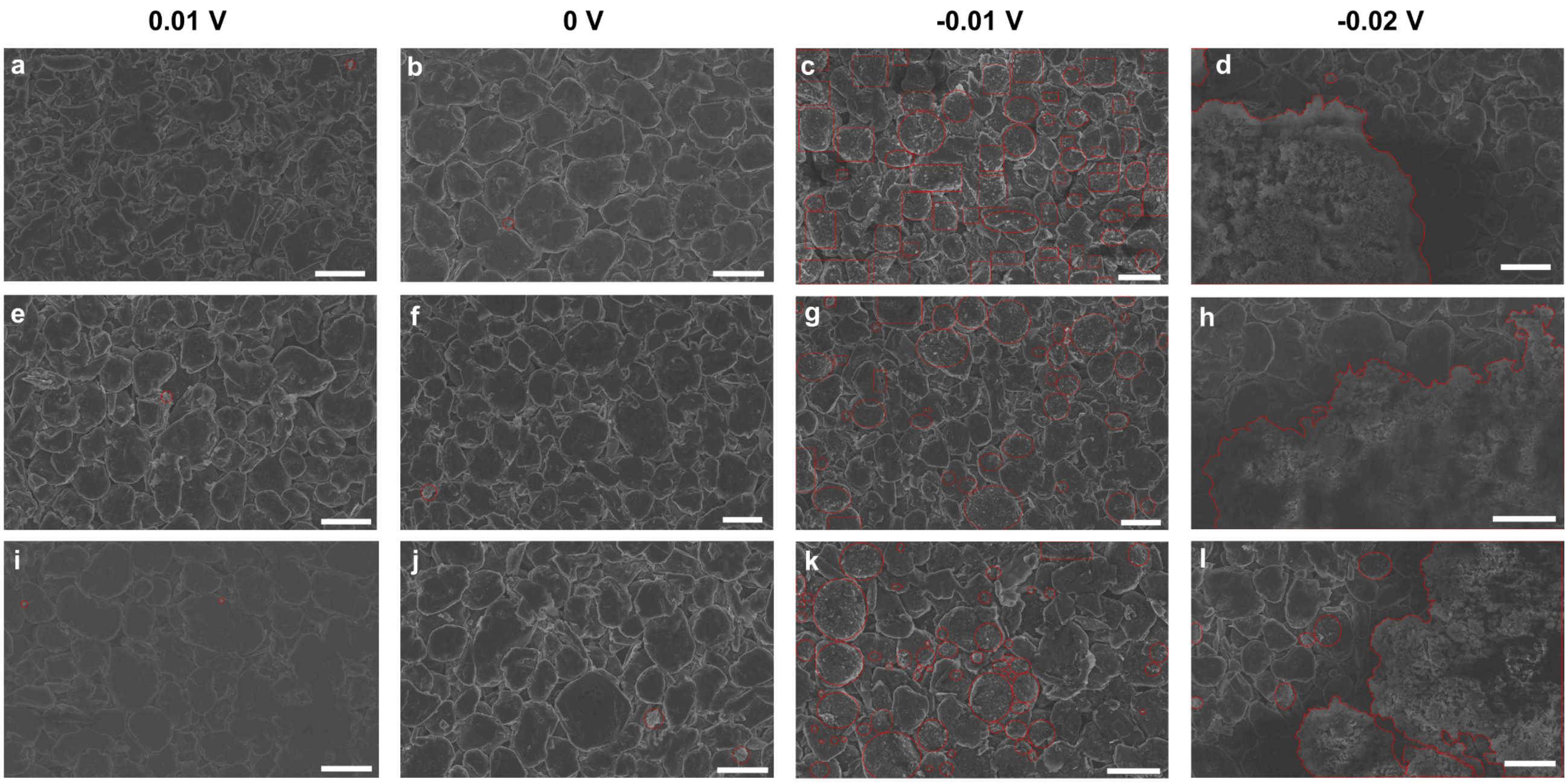

Figure S15. SEM images of graphite electrodes for predicted negative electrode potential of (a,e,i) 0.01 V; **(b,f,j)** 0 V; **(c,g,k)** -0.01 V; **(d,h,l)** -0.02 V. Figure 3f-i correspond one-to-one with Figure S15 a-d, respectively, and present cropped regions from the same SEM images for improved layout. All regions outlined in red indicate areas identified as lithium plating, including both Li particle nuclei and larger deposited lithium features. Scale bar: 20 μm

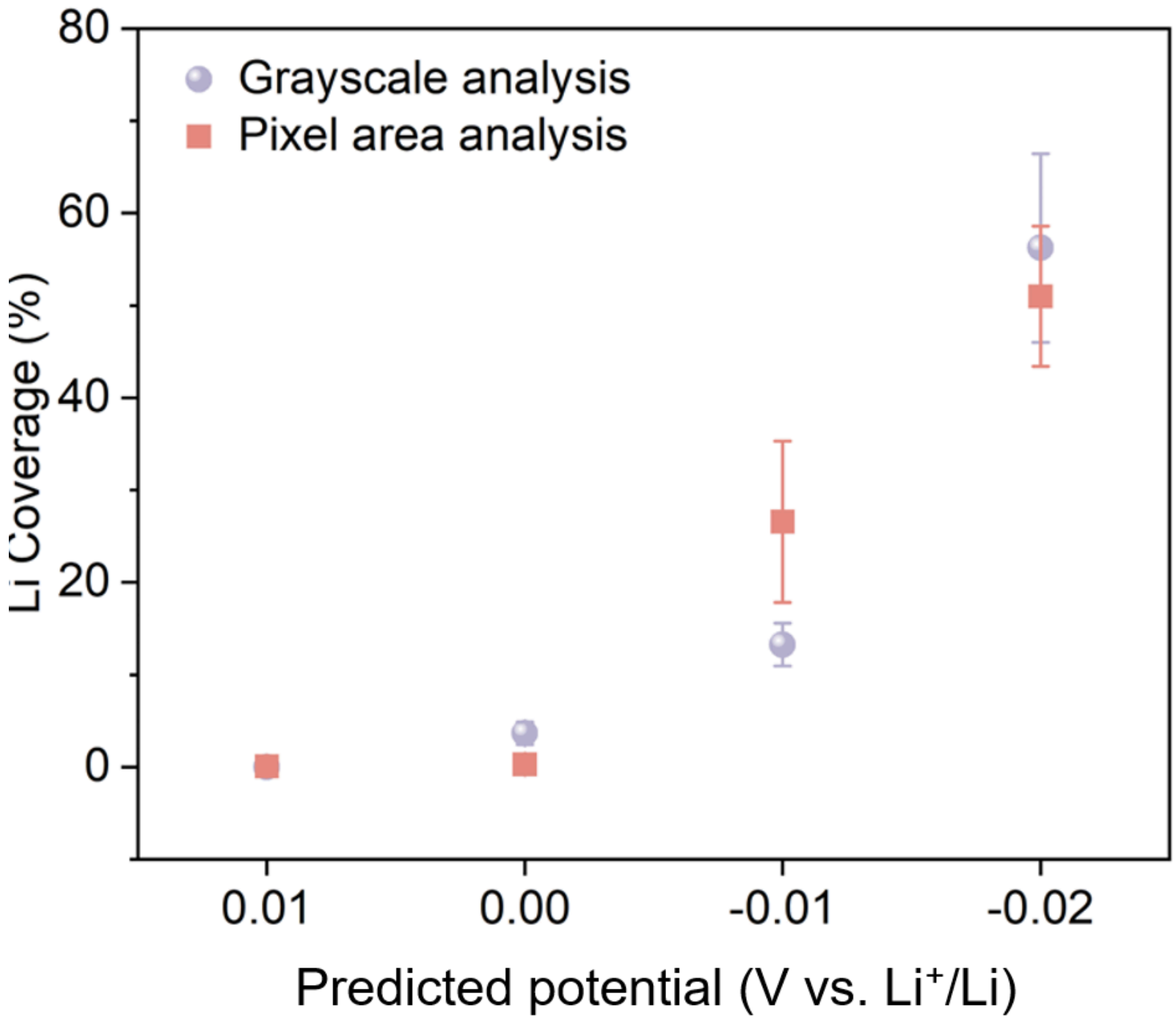

Figure S16. Li coverage with error bars for different predicted negative electrode potential. Li coverage was quantified from the SEM images in Fig. S15 via grayscale and pixel area analysis, by summing the pixel areas of all Li plating regions (including nuclei and large plating features, highlighted in red in Fig. S15) and normalizing by the total image pixel area. Data are presented as mean ± standard deviation across three cells per condition. Data are presented as mean values ± standard deviation from n = 3 independently cycled cells for each predicted negative electrode potential condition. Each data point corresponds to one independently cycled cell, and SEM images or quantified regions were not treated as additional independent replicates. Source data are provided as a Source Data file.

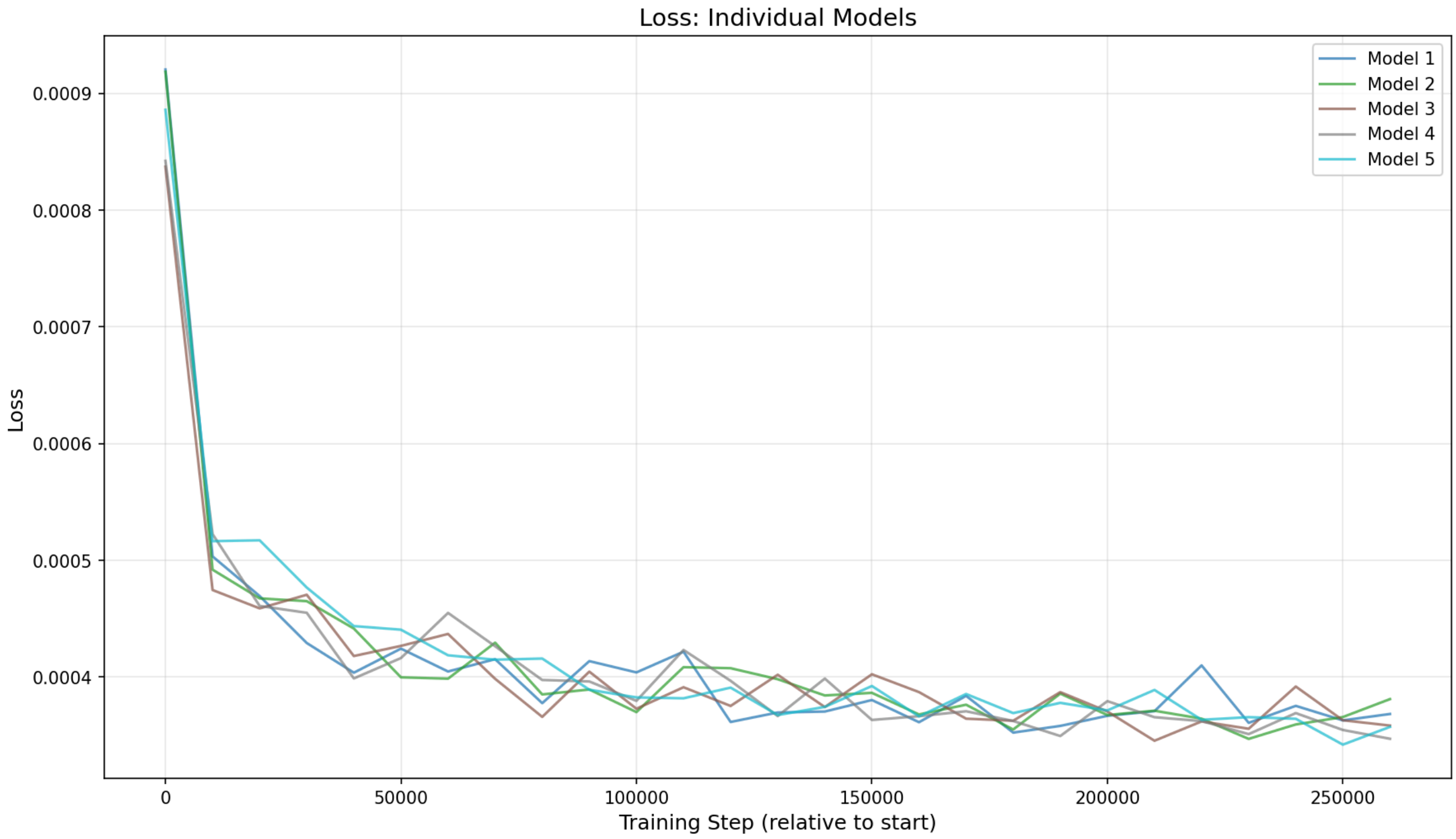

Figure S17. Test loss over the course of training

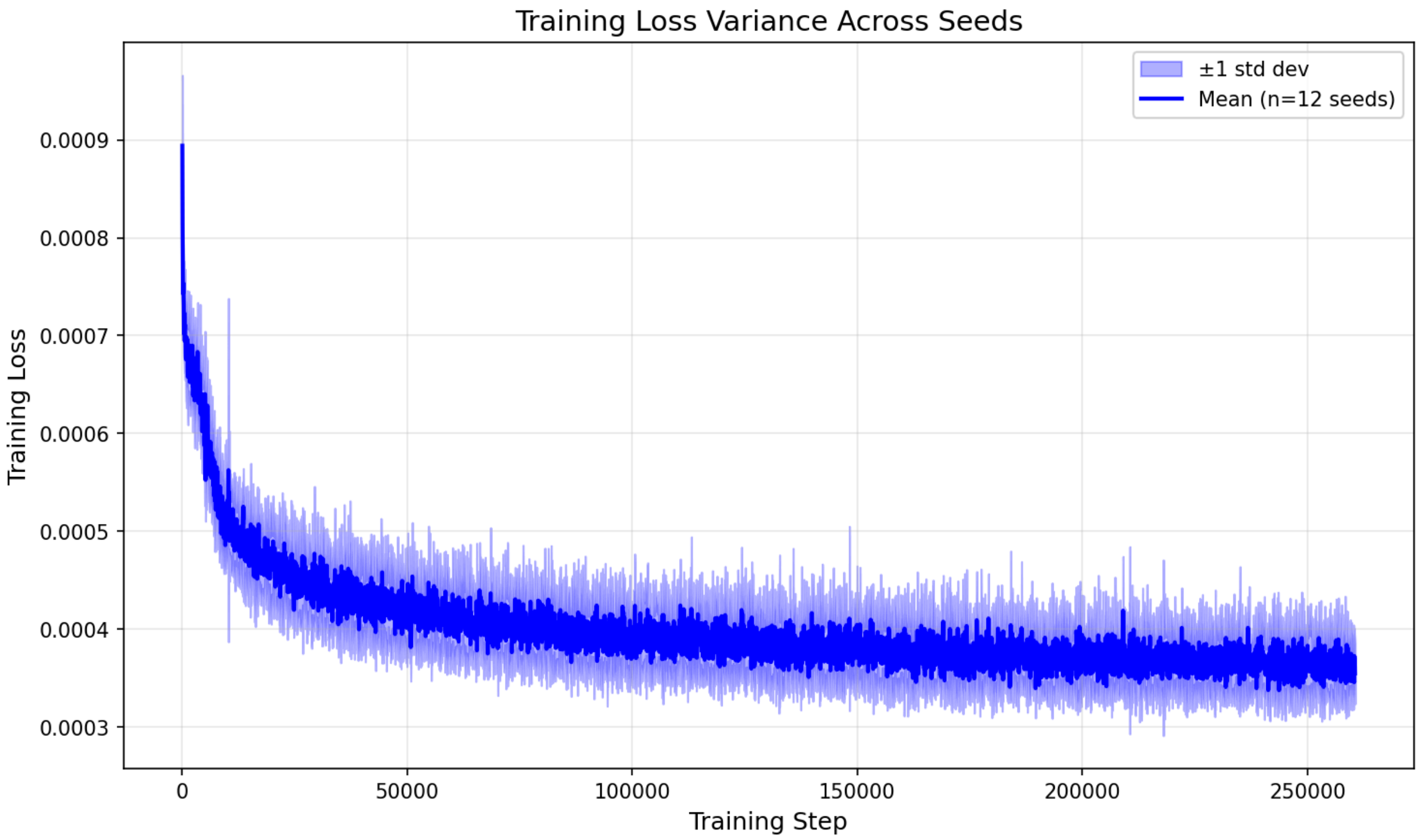

Figure S18. Mean and Std Dev of training loss across 12 different seeds. The dark line shows the mean while light blue shows ± 1 std dev

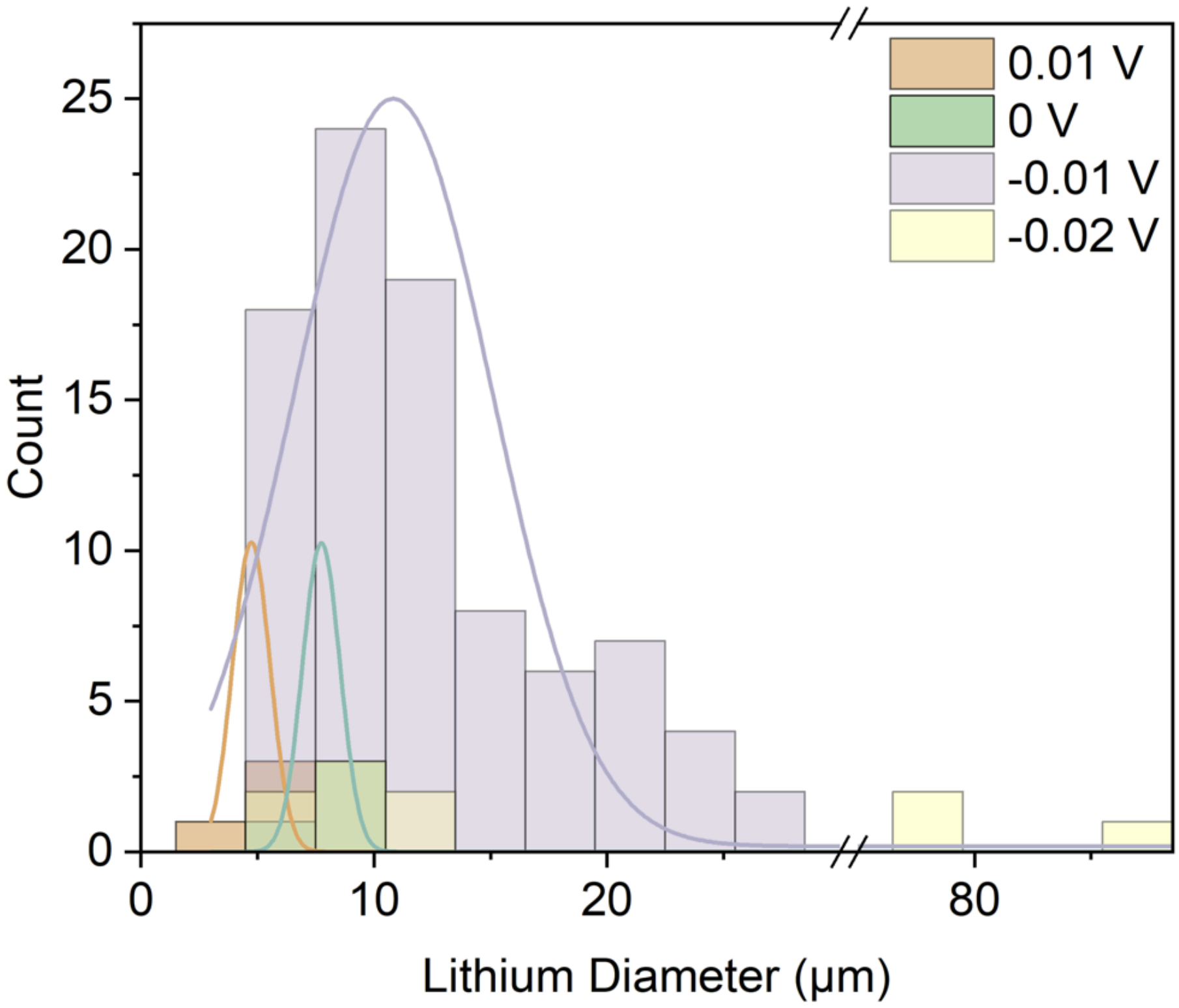

Figure S19. Li deposit size distribution analysis under different predicted NEPs. Results were collected from the SEM images in Fig. S15. Three independent cells were examined for each predicted NEP, with one representative SEM image acquired from the inner region of the graphite electrode from each cell, resulting in three SEM images per condition and 12 SEM images in total. The numbers of individually identified Li deposits analysed were 4 at 0.01 V, 4 at 0 V, 88 at -0.01 V, and 10 at -0.02 V. Each individually identified Li deposit was treated as the statistical unit for the size-distribution analysis, whereas the cell was the independent experimental unit. When the NEP is 0.01 V or 0 V, only sparse lithium deposition is present. When the NEP decreases to -0.01 V, a large amount of lithium deposition appears, with particle sizes ranging from 3 to 12 μm. As the NEP further decreases to -0.02 V, lithium deposition evolves from isolated spots to widespread surface coverage, with large lithium deposits appearing on the surface and particle sizes exceeding 70 μm. Source data are provided as a Source Data file.

Supplementary Tables

Table S1. Cell-by-cell charging protocol for 3E dataset

**3E Cell number index**	**Charging Protocol**
#1-#4	Protocol #1
#5-#9	Protocol #2
#10-#28	Protocol #3
#29-#47	Protocol #4
#48-#55*	Protocol #5

***** In protocol #5, The condition for the charging current density to change from 2.37 to 1.66 $mA \cdot cm^{-2}$ and from 1.66 to 1.18 $mA \cdot cm^{-2}$ is that the negative electrode potential is lower than a certain threshold to simulate dynamic charging. Negative electrode potential threshold: cell #48-#49: -0.15V; cell #50: -0.05 V; cell #51: -0.04 V; cell #52-#55: -0.01 V.

Table S2. Specification of Train-Test split for 3E dataset*

	Cell index	No. of datapoints	Ratio (%)
Training set	#1, #2, #4-#7, #9-#14, #16-#18, #20-#29, #32, #33, #35-#50, #52-#55	7,623,689	78
Test set	#3, #8, #15, #19, #30, #31, #34, #51	2,162,149	22

*Data partitioning is performed strictly at the cell level. All cycles from a given cell are assigned exclusively to either the training set or the test set. Therefore, no cycle-level or time-series data from the same cell appear in both training and test datasets, and no temporal information is shared across the split.

Table S3. All feature candidates before feature selection process

Feature Category	Feature List
Basic Features	Cell Voltage, Charging Current, Energy*, Capacity, Time, Power
Engineered Features	Throughput, Overpotential, dQ/dV, d(Cell Voltage)/dt, dEnergy/dt, dCapacity/dt, dOverpotential/dt,
Lagged Features ("-Y" means Y seconds ago)	X - 2, X - 4, X - 6, X - 8 (X represents all 13 features in both "Basic Features" and "Engineered Features")

*Energy is defined as positive energy value and calculated by the formula ∫I*V*dt, where I is the charging current, and V is the cell voltage.

Table S4. Robustness of our ensemble NN method in predicting C-rates not existing in training set

Training C-rates	Testing C-rates	# of Training Points	RMSE (V)	MAE (V)
0.33, 0.5, 0.8, 1.0, stepped **(Base model)**	0.33, 0.5, 0.8, 1.0, stepped	9.79 M	0.023	0.018
0.5, 0.8, 1.0, stepped	0.33	6.92 M	0.022	0.018
0.33, 0.8, 1.0, stepped	0.5	7.36 M	0.023	0.017

Table S5. False-negative analysis for smart-charging threshold settings

False-negative condition	False-negative rate(%)
Real NEP < 0 V && Predicted NEP ≥ 0.02 V	0.947
Real NEP < 0 V && Predicted NEP ≥ 0.01 V	1.445
Real NEP < 0 V && Predicted NEP ≥ 0 V	2.140

Table S6. Mean value, standard deviation (SD), and confidence interval (CI) analysis of Li coverage (%) calculated by pixel-area and greyscale methods under different predicted NEP conditions. The relatively wider CIs under negative NEP conditions reflect the localized and heterogeneous nature of Li plating on the graphite interface, highlighting the importance of follow-up research on localized lithium plating.

Predicted AV Group	Mean coverage (%)-pixel analysis	SD (%)-pixel analysis	95% CI (%)-pixel analysis	Mean coverage (%)-greyscale analysis	SD (%)-greyscale analysis	95% CI (%)-greyscale analysis
**0.01 V**	0.08	0.02	0.02 to 0.15	0	0	0.00 to 0.00
**0 V**	0.30	0.25	-0.32 to 0.93	3.67	1.22	0.64 to 6.70
**-0.01 V**	26.57	8.75	4.84 to 48.31	13.26	2.32	7.50 to 19.02
**-0.02 V**	50.99	7.60	32.09 to 69.88	56.22	10.21	30.86 to 81.58

Table S7. Correlation analysis between predicted NEP and Li coverage for different quantification methods*

Li coverage quantification method	Spearman's ρ	p-value	Pearson's r	p-value
Pixel area analysis	-0.950	2.25×10^{-6}	-0.924	1.77×10^{-5}
Greyscale analysis	-0.978	3.50×10^{-8}	-0.873	2.12×10^{-4}

Table S8. Generalization performance of the ML model under unseen charging protocols and different voltage domains.

Validation condition	RMSE(V)
Unseen 7-step smart charging*-1st cycle	0.0205
Unseen 7-step smart charging*-300 th cycle	0.0227
Train on CC cells only, test on stepped cells only	0.0382
3-3.6 V cutoff voltage domain, w/ CV stage	0.0303
**3.3-3.6 V cutoff voltage domain, w/o CV stage**	**0.0226**
3.3-3.4 V cutoff voltage domain, w/o CV stage	0.0209
3.4-3.5 V cutoff voltage domain, w/o CV stage	0.0194
3.5-3.6 V cutoff voltage domain, w/o CV stage	0.0288

***In this 7-step smart-charging protocol, charging starts at 1C and is reduced in 0.1C steps when the predicted NEP falls below 0.02 V until 0.4C. This protocol differs from the stepped protocol used in the training set, where a three-step 1C-0.7C-0.5C protocol was applied. The NEP thresholds used in the training-set stepped protocol were: -0.15 V for cells #48–49, -0.05 V for cell #50, -0.04 V for cell #51, and -0.01 V for cells #52–55.**

Table S9. Cell-to-cell variability analysis of ML prediction performance across cells in test set

	Mean	Std Dev
**RMSE(V)**	0.0263	0.0088
**MAE(V)**	0.0217	0.0085
**R^2**	0.821	0.071

Table S10. Effect of hidden-layer dimension on validation performance during hyperparameter tuning. The highlighted row indicates the final model used in this study.

**Hidden Size**	**RMSE(V)**	**R^2**
32	0.0472	0.73
96	0.0499	0.74
160	0.0443	0.76
224	0.0355	0.82
**256**	**0.0262**	**0.91**
288	0.0278	0.92
352	0.0311	0.86
416	0.0308	0.88
480	0.0373	0.80
544	0.0385	0.80

Table S11. Effect of hidden layer number on validation performance during hyperparameter tuning. The highlighted row indicates the final model used in this study.

**# of hidden Layers**	**RMSE(V)**	**R^2**
1	0.0512	0.71
2	0.0354	0.84
**3**	**0.0258**	**0.92**
4	0.0291	0.87

Table S12. Comparison of baselines and proposed model across RMSE, MAE, Latency, Parameter count, and Memory. All latencies and memory values were measured on a RTX3090

Model	RMSE(V)	MAE(V)	Latency (ms)	# of Parameters	Memory (MiB)
**Ensemble NN**	0.023	0.018	0.51	133K	13.7
**Single NN**	0.025	0.019	0.11	666K	11.7
**Linear Fit**	0.032	0.024	0.01	6	<1
**GPR**	0.039	0.029	0.05	325K	1.2
**Regression Tree**	0.049	0.039	0.02	350K	15.1

Supplementary Notes

Note #1. Stability of Li reference electrode

To prove the stability of Li metal reference electrode, we took out a used Li reference electrode in a Gr||LFP 3E cell, which has been cycled under 1C for 100 times, and we assembled a coin cell with the used Li RE and a piece of fresh Li metal to measure the voltage difference between them (**Figure S8**). 2-hour OCV measurement is performed after 12-hour rest because for all Gr||LFP cells in our dataset, 12-hour rest is applied before activation cycles for electrolyte to infiltrate all electrodes including Li RE. It is demonstrated that the voltage difference between them is **less than 0.3 mV**, indicating great stability of Li RE. It seems uncommon for reactive Li metal, but it makes sense because the reaction between fresh Li metal and electrolyte to form SEI initiates immediately when we add electrolyte on fresh Li during cell assembly (< 1s[1]). Furthermore, there is no current passing through the reference electrode throughout battery cycling, so there won't exist electrochemical corrosions as side reactions which will undermine the stability of RE and subsequent data collection reliability. Therefore, after a 12-hour rest with electrolyte, the chemical status of fresh Li metal will be close to used Li RE.

Note #2. Rationale of elements in our feature pool

The feature pool contains 65 features (Table S3). The first category is called basic features, which can be directly measured in BMS. From existing battery domain knowledge, the relationship between NEP and **Voltage, Energy, and Power** is intrinsic. With the increase of **battery voltage and charging current** (C-rate), as well as **charging time**, NEP will decrease. That is why high charging current and overcharge will initiate Li plating (NEP < 0). **Power** interprets the accumulation effect of **voltage** and **charging current**, while Energy demonstrates the accumulation of **voltage, charging current,** and **charging time**. Time itself is not selected by RFE in our work, which is good because NEP is a time-series parameter. If time is selected as an important feature, that could raise concerns for over-memorization and overfitting[2]. Secondly, we included engineered features, which are derived mathematically from basic features. Some of them are time-derivatives of basic features. Besides them, Throughput here is the number of total charges throughout the lifetime of a battery, indicating the aging effect on a battery. dQ/dV and overpotential are believed in past works to be diagnostic for Li plating. In the third category we added the lagged form of all features in basic and engineered features. Lagged features are widely used to improve the prediction of time-series parameters in daily life, such as Internet traffic forecasting[3], as well as time-dependent parameters in batteries (e.g. state of charge[4], state of health[5, 6]). In our work, NEP is a time-dependent parameter, so lagged features are introduced to capture NEP profile in stepped charging due to voltage relaxation behavior when charging current changes[7].

Note #3. Rationale of 0.02 V threshold in smart charging

In this study, the smart-charging controller uses a predicted negative electrode potential threshold of 0.02 V as the control limit for current regulation. This value was selected as a conservative engineering safety margin, rather than to imply that Li plating begins exactly when the spatially averaged NEP crosses 0 V.

Electrochemically, 0 V corresponds to the thermodynamic onset condition for Li plating. However, localized plating may already occur when the spatially averaged NEP remains slightly positive because of electrode heterogeneity, local overpotentials, and kinetic effects[8,9]. Accordingly, in the present framework, NEP < 0 V is interpreted as a physically meaningful plating-risk indicator rather than a strict binary classifier. Since the control objective is to more conservatively target Li-plating-free charging, the use of a threshold above 0 V is appropriate. We note that the false-negative analysis based on true NEP < 0 V is therefore a safety-relevant metric based on the spatially averaged/effective NEP, rather than a complete guarantee against all possible localized plating events.

The selection of 0.02 V was based on two complementary considerations. First, the virtual reference electrode achieves a mean absolute error (MAE) of approximately 0.018 V on the test set, such that a positive margin of similar magnitude provides a practical buffer against prediction uncertainty near the plating threshold. Second, we performed a false-negative analysis on the full 2.2 million-point test set near the threshold **(Table S5)**, defined as the fraction of cases where the true NEP was already negative while the predicted NEP remained above a candidate control threshold. The false-negative rates were 2.140% for a threshold of 0 V, 1.445% for 0.01 V, and 0.947% for 0.02 V. These results show that shifting the control threshold slightly above 0 V systematically reduces the probability of missing plating-risk points near the threshold. On this basis, 0.02 V was selected as a

conservative threshold because it was the first tested value that reduced the false-negative rate to below approximately 1%.

This conservative choice is further supported by prior literature showing that early plating nuclei or residual plated Li can facilitate subsequent Li deposition during later cycling, making suppression of even very small initial plating events desirable[10]. Consistent with this interpretation, the Li quantification in this study shows that Li coverage at a predicted NEP of 0.01 V is already very close to zero, whereas substantially greater coverage is observed once NEP becomes negative. As shown in Fig. S16, this trend was observed using both grayscale-based and pixel-area-based analyses across three cells per condition, supporting that small positive and near-zero predicted NEP conditions showed little detectable Li coverage within the examined fields of view. This near-threshold behavior supports the use of a control threshold above 0 V when the aim is not merely to delay severe plating, but to more conservatively avoid detectable plating.

To further assess the practical validity of this threshold, we performed a post hoc SEM analysis of the graphite electrode cycled under the 0.02 V smart-charging protocol shown in the **Fig. 4e&f** and **Fig. S11a&b**. Both grayscale-based and pixel-area-based quantification yielded 0% Li coverage for this protocol at end-of-life, supporting that the selected threshold successfully avoided detectable Li plating under the tested conditions.

Taken together, the 0.02 V threshold should be understood as a conservative engineering margin selected based on model uncertainty, false-negative analysis near the threshold, and experimental validation of plating-free behavior under the resulting control protocol.

Reference

1. Odziemkowski, M. & Irish, D.E. An Electrochemical Study of the Reactivity at the Lithium Electrolyte/Bare Lithium Metal Interface: I . Purified Electrolytes. *Journal of The Electrochemical Society* **139**, 3063 (1992).
2. Lin, R., Yu, C., Han, B. & Liu, T. On the Over-Memorization During Natural, Robust and Catastrophic Overfitting. *Preprint at https://arxiv.org/abs/2310.08847v4* (2024).
3. Cortez, P., Rio, M., Rocha, M. & Sousa, P. in The 2006 IEEE International Joint Conference on Neural Network Proceedings 2635-2642 (2006).
4. Dineva, A., Csomós, B., Kocsis Sz, S. & Vajda, I. Investigation of the performance of direct forecasting strategy using machine learning in State-of-Charge prediction of Li-ion batteries exposed to dynamic loads. *Journal of Energy Storage* **36**, 102351 (2021).
5. Ma, G. et al. Real-time personalized health status prediction of lithium-ion batteries using deep transfer learning. *Energy & Environmental Science* **15**, 4083-4094 (2022).
6. Shah, A.A. et al. Nonlinear autoregressive models for high accuracy early prediction of Li-ion battery end-of-life. *Journal of Energy Storage* **73**, 109014 (2023).
7. Shkrob, I.A., Rodrigues, M.-T.F. & Abraham, D.P. Fast Charging of Li-Ion Cells: Part III. Relaxation Dynamics and Trap-Controlled Lithium Ion Transport. *Journal of The Electrochemical Society* **166**, A4168 (2019).
8. Tanim, T. et al. Heterogeneous Behavior of Lithium Plating during Extreme Fast Charging. *Cell Reports Physical Science* **1**, 100114 (2020)
9. Wang, H. et al. Underpotential lithium plating on graphite anodes caused by temperature heterogeneity. *Proceedings of the National Academy of Sciences* **117**, 29453-29461 (2020)
10. Lin, Y. et al. Unveiling the Three Stages of Li Plating and Dynamic Evolution Processes in Pouch C/$LiFePO_4$ Batteries. *Advanced Energy Materials* **14**, 2400894 (2024).
