# Supplementary material for: Battery smart sensing via a virtual reference electrode
Source: Nat Commun. 2026 Aug 11;17:8336. doi: 10.1038/s41467-026-76535-y (PMC13473656; doi:10.1038/s41467-026-76535-y)
Supplement: Supplementary file 2 [file 41467_2026_76535_MOESM2_ESM.pdf]

nature portfolio

Peer Review File

Battery smart sensing via a virtual reference electrode

Corresponding Author: Professor Yuzhang Li

Parts of this Peer Review File have been redacted as indicated to remove third-party material.

This file contains all reviewer reports in order by version, followed by all author rebuttals in order by version.

Attachments originally included by the reviewers as part of their assessment can be found at the end of this file.

Version 0:

Reviewer comments:

Reviewer #1

(Remarks to the Author)
This paper proposes a method of virtual reference electrode sensor based on artificial intelligence, which is very meaningful for the problem it addresses, but I think this paper does not provide solutions to the problems that readers may be concerned about, such as commercial batteries and ultra-fast charging. The author can consider the following suggestions:
1. The paper is not divided into chapters, so the logic and readability of the whole paper need to be improved.
2. The proposed method is based on coin cells, and lacks verification of the scalability of this method on commercial batteries that readers are more concerned about.
3. A higher charging rate or a battery in the late aging stage both may cause a larger overpotential of the battery. Can this method distinguish them? The prediction accuracy of this method at different aging stages, especially in the late aging stage, needs to be analyzed.
4. The robustness of this method needs to be analyzed. The prediction accuracy of this method when encountering a charging rate that does not exist in the training set is lacking, and the prediction results under the condition where the charging rate changes with time are not shown.
5. In the final verification of smart charging, a 3E battery cell should be used at the same time to verify the accuracy of this method in predicting the anode potential over the entire life cycle.

Reviewer #2

(Remarks to the Author)
SUMMARY

This manuscript proposes a virtual sensor to measure anode voltage, for the purpose of mitigating lithium plating at the anode-separator interface. This virtual sensor is, ultimately, an ensemble of 4-layer feedforward neural nets that predict anode voltage from measured voltage and current signals. This machine learning model is trained from data collected from coin cells with reference electrodes.

The overall idea is intriguing, original from my experience, and has merit. However, the execution of the research needs significant improvements. Comments are categorized below as MAJOR and MINOR:

MAJOR COMMENTS:

1. [Ensemble Neural Networks] Do all the feedforward neural networks have identical structures and training processes? If yes, then it’s unclear why an ensemble approach is used versus simply adding 5X more nodes in the inner layers. Please explain, as the two approaches appear functionally (and perhaps mathematically) equivalent.

2. [High C-rate Data collection] It seems the highest current loading is 2.37 mA-cm2 and the cells have an areal capacity of 2.37 mAh-cm2. In other words, the data is collect at 1C charging rate and lower. However, many LFP battery applications experience higher C-rates, where lithium plating is a significant risk. For example, the recently publicized Zeeker “Golden Brick Battery” – an LFP chemistry – charges at up to 5.5C. Please explain why higher C-rates were not used, which is precisely when the problem of lithium plating is more acute.

3. [“AI model” versus “ML model” used throughout the paper] The manuscript is quite liberal about using “AI model” and “ML model” interchangeably. This is not only confusing, but misleading. I strongly urge the authors to replace any mention of “AI model” with “ML model”. Specifically, an AI model is an algorithm which performs human-like tasks, including reasoning, problem solving, perception, and language understanding. An “ML model” is a specific subclass of AI models focused on learning patterns from data, using statistical and optimization methods. Without question, this manuscript falls in the narrow category of “ML models”. To be honest, I am irked by the framing of the proposed AV prediction model as “AI” instead of “ML”, which gives the impression of salesmanship over scientific rigor.

4. [Fig. 4d] It is strange that cell voltage is higher for CC-CV versus smart charging – at the same SOC. Can you explain this? Specifically, let’s focus on the CC stage for both protocols. Smart charging uses a higher C-rate (2.37 mA-cm2) whereas the CC-CV baseline uses a lower C-rate (2.23 mA-cm2). Assuming the cells are equivalent, I would expect smart charging to have a higher voltage, because of greater ohmic overpotential. However, the opposite happens in Fig. 4d. Please explain.

5. [Comparison to Open-Loop Physics models – MOST IMPORTANT COMMENT] Ultimately, the proposed ML algorithm produces a RMSE of 23 mV, which is about 5% error considering the AV values range from +450 mV to -50mV. Meanwhile, the manuscript claims the proposed ML-powered “virtual sensor” outperforms physics-based models. I request that the authors directly compare their ML model to a state-of-art open-loop physics-based model. Relevant benchmark open-loop models can be found in the following references:

- https://doi.org/10.1109/TCST.2011.2178604
- https://doi.org/10.1109/TCST.2021.3135149

Specifically, the authors should justify their contribution of ~5% RMSE to be better than a physics-based model. This manuscript is essentially a ML paper, and just like all ML papers, the authors need to compare against the state-of-art benchmarks. I would not expect a lower level of scientific rigor in Nature Communications, and the editorial board should not expect a lower level.

6. [Smart Charging] The proposed smart charging, which experiences chattering, is a strange choice. The natural choice is CC-CAV. That is, once the AV reaches 20mV, for example, then control current to hold this value. This would eliminate chattering and potentially further improve longevity. Can the authors comment on why they applied the chattering approach instead of CC-CAV? Obviously, this is more involved to implement experimentally. But that’s a simple engineering problem. I want more explanation beyond “an engineering challenge”.

7. [Scalability: From Coin cells to commercial cells] Please comment on the challenges of scaling this approach from single-layer coin cells to commercial cells that could be 50Ah or more.

MINOR COMMENTS:

8. [Line 57] What are “N/P ratios”? This terminology should be defined. Most of the more general audience of Nature Communications will not know this jargon.

9. [Line 112] Supplementary Fig. 4 does NOT demonstrate that an ensemble of 5 feedforward NNs provide the highest accuracy. This figure compares linear regression with GPR. Please correct the reference.

10. [Line 120] Comparing a 4-layer NN to LLMs is outrageous. Comparing the prediction of thenext words (tokens to be exa ct) is remarkably more complex than predicting the next AV values (which decrease monotonically). Obviously, the training resources for LLMs are orders of magnitude more than those used for the NNs in this paper. Please remove the comparisons with LLMs. It is incredibly distracting to the reader, and provides no substantive value to the paper.

11. [Equation for “Energy” feature] Please provide this equation in the supplementary feature. It is a crucial part of the AV prediction model. For clarity and to enable others to reproduce the work, the equation should be included.

12. [Line 185] missing a period.

13. [Fig. 4a and 4b] Why are the x-axes and y-axes missing values? This is very strange. Please provide, or explain why you are hiding data from the results?

14. [Fig. 4c] It is notable that the baseline CC-CV LFP cell reaches end-of-life at only 100 cycles, considering that high-quality commercial LFP cells have a lifetime of 3000 – 5000 cycles. Can the authors explain this further? The N/P ratio of 1.0 is obviously part of the reason. But is there something else occurring?

Reviewer #3

(Remarks to the Author)
The authors examine the problem of rapidly charging a lithium ion battery. They argue, convincingly, that this is a very important problem in the literature. Then they point out that battery safety is an issue during fast charging due to lithium plating, which can also compromise battery useful life. Again, this is an excellent point, and it is very well-established in the literature. Then they argue that they can use artificial intelligence to develop a virtual reference electrode. They execute this

task, and report an estimation error of 25mV. This allows them to improve battery life during fast charging by a reported 8.5-fold improvement.

The ideas behind this manuscript are sound. However, the manuscript itself is unfortunately very weak, and should not be accepted for publication in its current form. For one thing, contrary to the authors' main claim, the virtual reference electrode is neither a novel or innovative addition to the literature. I encourage the authors to just go to Google Scholar and type the words "virtual reference electrode" into the search box. They will immediately see work in this area spanning multiple decades. For another thing, an estimation error of 23mV is not good at all when trying to avoid lithium plating. The over-potentials driving such plating are usually an order of magnitude smaller, which means that the reported estimation error may be too high for the purpose of preventing plating. Last but not least, the paper is a little too strong on hype (with too much of the hype being in boldface font), and a little too weak on substance. The ultimate metric for the quality of a paper, in terms of substance, is the degree to which it is written with enough clarity and specificity to the point of being reproducible. As currently written, I doubt that anyone can easily reproduce the work in this paper without excessive digging into supplemental materials - and in fact, even with such digging, I am not sure that the work would be truly reproducible.

Perhaps with some very significant rewriting, the paper can become publishable. In particular, I do want to give the authors credit where credit is due by emphasizing the fact that I have not yet seen an ML-based virtual reference electrode in the literature, and have not yet seen a credible, scholarly, hype-free comparison of such an ML-based virtual reference electrode versus a physics-based virtual reference electrodes. Those particular elements of the work may indeed be novel, and I would be excited to see them in publication. One challenge I see with this work is its excessive emphasis on hype at the expense of emphasizing the more thoughtful elements of the work. Hopefully the authors can revise the work with this feedback in mind.

Version 1:

Reviewer comments:

Reviewer #1

(Remarks to the Author)
The topic of this study is very interesting. However, unfortunately, the following three points still make me feel that this manuscript does not meet the strict requirements of Nature Communications:
(1) The current estimation results are based on a large number of experiments conducted on coin cells. The authors have not yet clearly explained how this work can be applied to commercial high-capacity batteries, as it is difficult to obtain such a large amount of anode-voltage data in commercial batteries.
(2) The anode potential is highly sensitive to charging rates and testing environments. The current estimation error is, in my opinion, too large to meet application requirements. As shown in Figure S12, the measured anode potential is already below 0 V near 80% SOC, which will directly trigger lithium plating, while the predicted anode potential remains above 0 V, failing to provide warning.
(3) Ultra-fast charging is an inevitable trend. The 1 C rate mentioned in the manuscript is insufficient to provide further guidance for researchers in this field. Research on lithium plating should instead focus on high-rate or low-temperature conditions.

Reviewer #2

(Remarks to the Author)
Please see attached

Version 2:

Reviewer comments:

Reviewer #1

(Remarks to the Author)
(1) On page 8, the test dataset was directly used in the comparison of results for different models (see Supplementary Fig. 13), and the authors ultimately chose an ensemble of five feedforward neural networks. However, I think that the test dataset should not be used during the model selection process, as this can contaminate the model and lead to overfitting. I believe that the number of models in ensemble also falls under the category of hyperparameters.
(2) When the proposed ensemble model is trained, are the five neural networks trained individually, or are they trained as a whole based on the average of their predictions?
(3) Table S4 illustrates the robustness of the ensemble NN method in predicting C-rates not existing in the training set. Considering that this model will mainly be used to regulate charging strategies in the future, is it feasible to use 0.33 C, 0.5 C, 0.8 C, and 1.0 C as the training set and stepped protocol as the test set? Or, what is the prediction accuracy of the neural network in the entire process of smart charging protocol (which I believe is also a new stepped condition not present in the training set) shown in Fig. 4? The relevant results may be very convincing for the promotion and application of this network.

Reviewer #4

(Remarks to the Author)
1.You split 78/22 by the number of per-second measurements. Please clarify whether different cycles from the same cell can appear in both train and test, which would cause time-series leakage and inflate generalization performance.
2.The SI states you removed CC data below 3.3 V and all CV data. Real BMS charging often includes CV or multi-stage strategies. How will the model behave when CV/low-voltage segments are present, and how do you avoid deployment distribution shift caused by this exclusion?
3.You use AV<0 as the thermodynamic plating criterion, but real plating depends on kinetics, temperature, overpotential, and local current density. How does the method handle scenarios where AV is slightly above 0 yet localized plating occurs?
4.SEM validation uses two points (predicted 0.01 V and -0.02 V). Is this anecdotal? Please provide more SOC points and replicated cells (e.g., n≥3), and include quantitative metrics (coverage, deposit thickness distribution), not only qualitative images.
5.You emphasize N/P≈1.0 as a stress test, but many commercial cells have higher N/P. Please discuss whether AV distributions and prediction error change for typical N/P (1.1–1.2), and whether smart-charging benefits remain significant.
6.Each ensemble network uses two 256-unit layers, 70 epochs, and 20% dropout. Please provide learning curves, early stopping/regularization details, variance across random seeds, and justify why such a large network is necessary with only 5 input features.
7.The response and SI says parameters are tuned using measured voltage-current over many cycles. Please clarify whether the PBM is re-tuned per cycle or tuned early and extrapolated.
8.Smart charging uses a C# interface to the cycler and calls Python models. Please report the end-to-end closed-loop latency (sampling→prediction→current update) and describe fail-safe strategies under jitter, packet loss, or outliers.

Version 3:

Reviewer comments:

Reviewer #4

(Remarks to the Author)
1.The rebuttal usefully shows that when the model is trained only on constant-current charging data and tested on stepped-charging protocols, the test error increases from approximately 23 mV to approximately 38 mV. While this supports some degree of extrapolative capability, I would still encourage the authors to more systematically define the generalization boundary of the model with respect to unseen charging protocols. Beyond stepped charging, has the model been evaluated under more complex multi-stage protocols, different cutoff voltages, different rest durations, or different temperature conditions? Providing error statistics stratified by protocol type, rather than only an aggregate RMSE, would help clarify the model's practical applicability under realistic BMS operating scenarios.
2.The authors have clarified that the train/test partition is performed strictly at the cell level, which avoids leakage across cycles from the same cell; meanwhile, the PBM comparison relies on a one-time early-cycle calibration for each cell, with fixed parameters thereafter. In light of this, I encourage the authors to state more explicitly whether the ML model requires absolutely no per-cell calibration when deployed on a new cell. In addition, the current validation still appears to remain within one chemistry and one experimental distribution. If the model is transferred to cells from different manufacturing batches, electrolyte formulations, electrode loadings, or broader cell-to-cell variability, can it still be used directly? An external validation across batches or manufacturing variability would substantially strengthen the claim of general applicability.
3.The rebuttal appropriately acknowledges that localized lithium plating may already occur when the average AV remains slightly above 0 V, and therefore AV < 0 V should be interpreted as a physically meaningful thermodynamic risk indicator rather than a strict binary classifier. Since the proposed smart-charging strategy is built around this indicator, I would encourage the authors to further clarify how the framework avoids false negatives in the near-threshold regime where AV is still slightly positive but localized plating may already emerge. At present, the 0.02 V safety buffer appears to be chosen primarily based on the reported MAE of about 0.018 V. A more direct risk-oriented analysis, such as a threshold sensitivity study or a false-positive/false-negative tradeoff near the plating threshold, would make the control rationale more convincing.
4.The authors have substantially strengthened the SEM validation by expanding it to four AV conditions with n = 3 cells per condition and by adding quantitative lithium-coverage analysis, which clearly improves the manuscript. However, the quantitative validation still appears to rely mainly on a two-dimensional surface coverage metric. I would encourage the authors to provide more complete statistical information, such as raw per-sample coverage values, field-of-view variability, confidence intervals, and—if available—deposit size or thickness distributions. In addition, since the rebuttal emphasizes a clear correlation between predicted AV and the extent of lithium deposition, it would be valuable to support this claim with a more formal statistical analysis rather than a trend display alone.
5.Although the final model uses only five input features, the authors employ a two-layer network with 256 hidden units per layer and an ensemble of five independently trained models. The rebuttal adds learning curves, seed variance, L1 regularization, and the qualitative explanation that the learned function requires a wide network; however, the justification remains somewhat empirical. I would encourage the authors to provide a more systematic ablation study, for example by comparing narrower architectures, non-deep-learning baselines such as linear or tree-based models, different lag-feature combinations, and different ensemble sizes. Such analyses would better demonstrate that the chosen architecture is not simply over-parameterized, but instead reflects a meaningful tradeoff among accuracy, robustness, and real-time deployability.
6.The additional estimate of an end-to-end closed-loop latency of about 115 ms, which is much smaller than the 2 s sampling interval, is helpful. However, for practical BMS deployment, average latency alone is not sufficient. A key concern is whether the system includes explicit fail-safe logic and fallback control policies under abnormal conditions such as communication jitter, packet loss, outlier inputs, abrupt prediction spikes, or model execution failure. The current rebuttal

mainly reports average latency, but does not yet describe worst-case latency, anomaly handling, or conservative fallback strategies. I encourage the authors to provide these implementation details to better support the practical deployment claim.

Version 4:

Reviewer comments:

Reviewer #4

(Remarks to the Author)
1.The added transfer-learning results are useful, but can the authors clarify how much target-domain data was used for fine-tuning, whether the split was strictly cell-level, and whether the original trained model has any zero-shot performance on the other LFP, NCM811, electrolyte, and pouch-cell datasets before transfer learning?
2.The 0.02 V threshold reduces the false-negative rate below 1%, but what is the corresponding false-positive rate and impact on charging time/average charging current? Could the authors provide a threshold-sensitivity table including false negatives, false positives, charging speed, and cycle-life outcome if available?
3.Since localized plating may occur even when spatially averaged AV is slightly positive, can the authors clarify whether the false-negative analysis based on true AV < 0 V may underestimate missed localized plating events, and whether SEM data at small positive predicted AV values, e.g., 0.005–0.02 V, support the selected safety margin?
4.The added SEM quantification is valuable, but can the authors report field-of-view-level variability or clarify how many SEM fields were analyzed per cell? Also, because the correlation analysis uses only four AV levels with three cells each, can the authors confirm that the p-values are not inflated by treating repeated AV-level observations as fully independent?
5.The ablation study supports the final network, but can the authors reconcile the stated architecture description with Table S11 and provide a compact table comparing final ENN, single NN, linear/GPR/tree baselines in terms of RMSE, MAE, inference latency, parameter count, and memory footprint under the same test set?

Open Access This Peer Review File is licensed under a Creative Commons Attribution 4.0 International License, which permits use, sharing, adaptation, distribution and reproduction in any medium or format, as long as you give appropriate credit to the original author(s) and the source, provide a link to the Creative Commons license, and indicate if changes were made.
In cases where reviewers are anonymous, credit should be given to 'Anonymous Referee' and the source.
The images or other third party material in this Peer Review File are included in the article's Creative Commons license, unless indicated otherwise in a credit line to the material. If material is not included in the article's Creative Commons license and your intended use is not permitted by statutory regulation or exceeds the permitted use, you will need to obtain permission directly from the copyright holder.
To view a copy of this license, visit https://creativecommons.org/licenses/by/4.0/

Comments to the Authors

Title: Battery smart sensing via a virtual reference electrode

Manuscript Number: NCOMMS-25-27724A

I appreciate the authors' thorough efforts to address my comments. The effort is impressive. Meanwhile, I wish to reiterate that the proposed idea is novel and impactful, which is why I am motivated to assist in the review process. Also I acknowledge that some comments may not seem directly relevant to the conclusions. However, they are relevant to readers with my expertise. Thank you for considering them.

All that said, I am still unsatisfied with one response. Specifically:

R2-5: This was my MOST important comment, because it is crucial to the manuscript's claimed contributions. However, the authors are avoiding a direct comparison to a physics-based model. I ask again, please perform a comparative analysis, instead of shrugging off the suggestions as irrelevant. The authors claim that 23mV RMSE is better than state-of-art physics-based models. If this is their claim, then they must PROVE it, no? Specifically, they can and should do the following:

Indeed, most existing physics-based models do not adapt their parameters to reflect aging, and this would introduce errors in predicting AV. Great! This gives the authors' approach an inherent advantage! The key question is this - which has greater error, physics-based models (with parameters that do not adapt) or the proposed ML approach? If the authors reject my suggestions for a physics-based model comparison, then they can select the one that is most appropriate in their view.

Measured AV via a 3rd reference electrode is obviously the best physics-based prediction. But installing a 3rd reference electrode is not practical in commercial applications. Meanwhile, the state-of-art competing technology would be a physics-based state observer using control theory. This is the gold standard. A simple physics-based state observer is an open-loop observer, which is simply a prediction model. This prediction model will be imperfect, in some sense. For example, the parameters may not adapt to aging. But this approach is still the gold standard. This is why I am insistent on comparing the key contribution to the gold standard / state-of-art approach.

Response Letter to Reviewers' Comments

Manuscript ID: NCOMMS-25-27724

Title: Battery smart sensing via a virtual reference electrode

Authors: Jiayi Yu, Aki Takahashi, Min-Ho Kim, Rishi Upadhyay, Howard Zhang, Tianyu Wang, Bo Liu, Xintong Yuan, Thomas Hymel, Kaiyan Liang, Keyue Liang, Haoyang Wu, Dingyi Zhao, Jung Tae Kim, Achuta Kadambi, Yuzhang Li

Response to Reviewer #1

Summary: *This paper proposes a method of virtual reference electrode sensor based on artificial intelligence, which is very meaningful for the problem it addresses, but I think this paper does not provide solutions to the problems that readers may be concerned about, such as commercial batteries and ultra-fast charging. The author can consider the following suggestions:*

Authors' Response: We appreciate Reviewer #1 for recognizing the novelty of our work and thank the reviewer for the positive feedback on our study. We take this opportunity to respond to each comment in detail below.

Specific Comments R1-1. *The paper is not divided into chapters, so the logic and readability of the whole paper need to be improved.*

Authors' Response: We appreciate Reviewer #1's suggestion. To improve the logic and readability of the manuscript, we have added subtitles to clearly delineate each section.

In the revised manuscript:

1. Data Generation and Machine-learning model performance
2. Real-time validation of ML model
3. Implementation of ML model on cyclelife improvement

Specific Comments R1-2. *The proposed method is based on coin cells, and lacks verification of the scalability of this method on commercial batteries that readers are more concerned about.*

Authors' Response: We appreciate Reviewer #1 for pointing it out. First we would like to reiterate that our research paper aims to provide **a proof-of-concept for machine-learning-driven**

virtual reference electrodes based on real-world experimental data instead of simulated data. Our model can accurately predict anode voltage without the need for a physical reference electrode—a capability that is especially valuable given that traditional physics-based models often fall short in capturing the complex, nonlinear and time-evolving behaviors of real batteries.

While full-scale demonstrations are still in progress due to limitations in funding and resources, we strongly believe that our approach lays a critical foundation for future implementation in practical BMS applications, where data-driven adaptability and real-time monitoring are essential.

Although our initial validation was conducted in coin cell configuration—a widely used and controlled benchmark platform in lab-scale battery research, we fully recognize the importance of demonstrating scalability of our method. To that end, we are glad to show some preliminary results applying our ML model on prediction of anode voltage measured in **Gr|NCM523 3-electrode pouch cells** (**Table R1**). These results help address concerns on applicability of our model/approach in commercial batteries with higher capacity and different architecture.

**Cell type**	**RMSE (V)**
LFP coin cell	0.023
NCM523 pouch cell	0.045

Table R1. Model performance in scalability verification tested on commercial pouch cells equipped with Li reference electrode

Specific Comments R1-3. *A higher charging rate or a battery in the late aging stage both may cause a larger overpotential of the battery. Can this method distinguish them? The prediction accuracy of this method at different aging stages, especially in the late aging stage, needs to be analyzed.*

<u>**Authors' Response**</u>: We thank Reviewer #1 for this comment. In **Figure S6**, we plotted the model performance under different charging rates, and different aging stages. We here specify the C-

rates for all individual cell in test set in revised Figure S6, as below, and add the demonstration to highlight that in main text

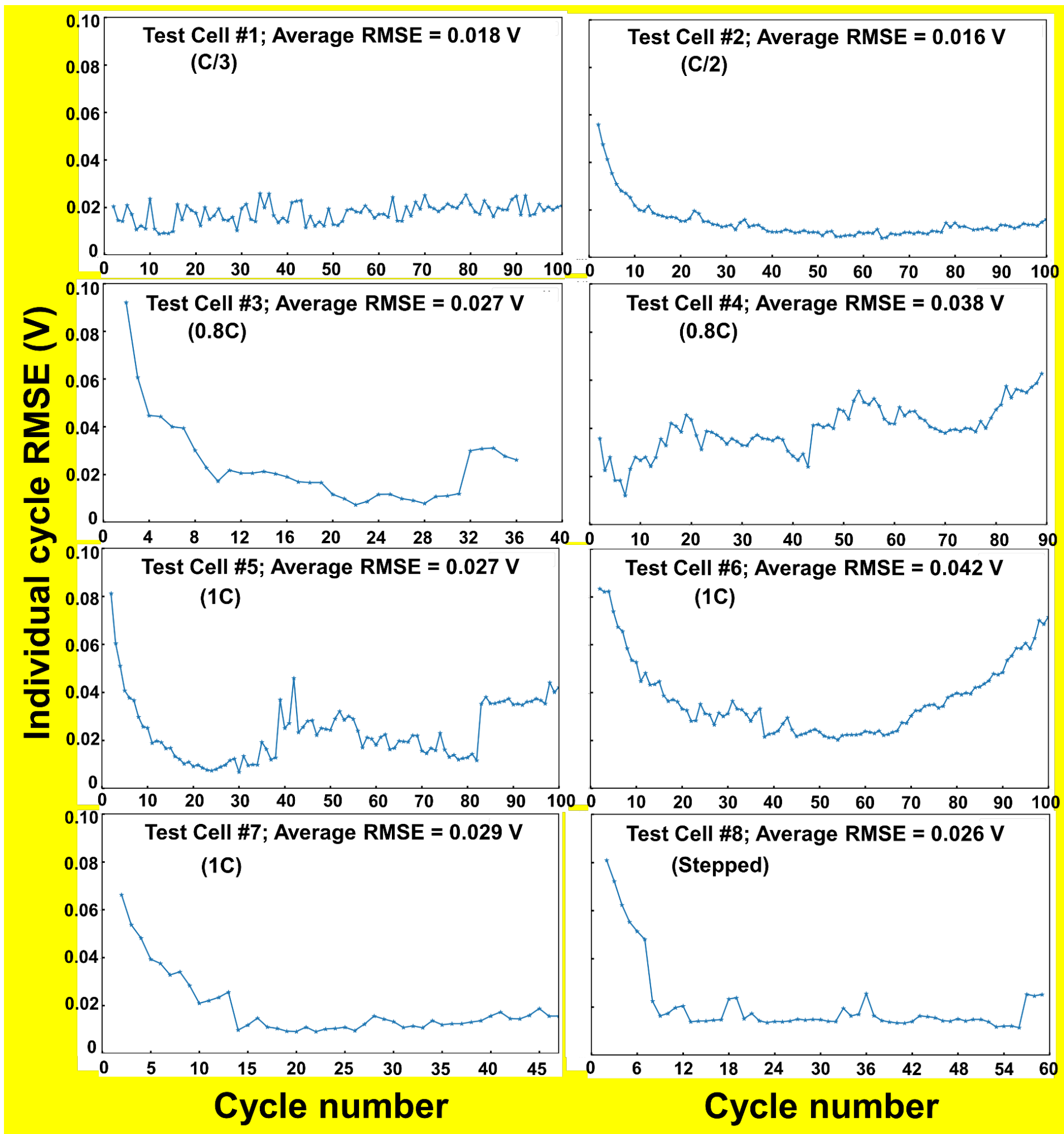

Figure S6. Error metrics of ensemble neural network for individual cycle of all 3E cells in the test set

<u>Manuscript edit</u>:

Not only are these error metrics competitive with even the best performing physics-based simulation[26, 39, 47], but also remain robust throughout battery lifetime without a decay in accuracy across multiple C-rates(see Supplementary Fig. 6). This is largely due to the variety of cycling conditions and aging data included in our training set. Furthermore, the consistency of model performance indicates the robustness of our model against overfitting

Specific Comments R1-4. *The robustness of this method needs to be analyzed. The prediction accuracy of this method when encountering a charging rate that does not exist in the training set is lacking, and the prediction results under the condition where the charging rate changes with time are not shown.*

__Authors' Response__: We appreciate Reviewer #1 for pointing it out in this test. In order to evaluate how well our model generalizes to new C-rates, we trained new models where we purposefully hold out all cells with specific C-rates during training and then evaluate our model on those cells. Here is a table of our results:

**Training C-rates**	**Testing C-rates**	**# of Training Points**	**RMSE (V)**	**MAE (V)**
0.33, 0.5, 0.8, 1.0, stepped **(Base model)**	0.33, 0.5, 0.8, 1.0, stepped	9.79 M	0.023	0.018
0.5, 0.8, 1.0, stepped	0.33	6.92 M	0.022	0.018
0.33, 0.8, 1.0, stepped	0.5	7.36 M	0.023	0.017

Table R2. Robustness of our ensemble NN method in predicting C-rates not existing in training set

As shown in **Table R2**, for 0.33 C and 0.5C, despite never seeing them during training, the model performs just as good as it does when it has seen all the C-rates during training.

Furthermore, in smart charging, a 3E battery cell was used to verify the accuracy of the model over its entire cycle life **(Figure S12)**. During our smart charging, there are several steps (0.9C, 0.7C, 0.6C, 0.4C) that are not included in our training set (C/3, C/2, 0.8C, 1C). However, the RMSE error in the last cycle(22.7 mV) is comparable with the beginning of life (20.5 mV), and they are as good as the RMSE of our model on average in **Fig. 2** (23 mV). That demonstrated the robustness of our model in predicting the conditions not included in the training set.

Manuscript edit:

1. Whereas metallic Li is clearly observed both optically and with SEM in fully discharged CC-CV cells at end-of-life (80% capacity retention) **(Fig. 4f)**, the surface of the graphite anode remains smooth and pristine in smart BMS cells after smart charging **(Fig. 4e & Supplementary Fig. 11)**. At the same time, a 3E cell is applied throughout the cycle life of smart charging protocol to verify the accuracy of this method in predicting the anode potential over the entire life cycle. The high and consistent accuracy between measured and predicted AV verified the robustness of our model while dealing with long-cycled batteries and unseen charging C-rates **(Supplementary Fig. 12)**

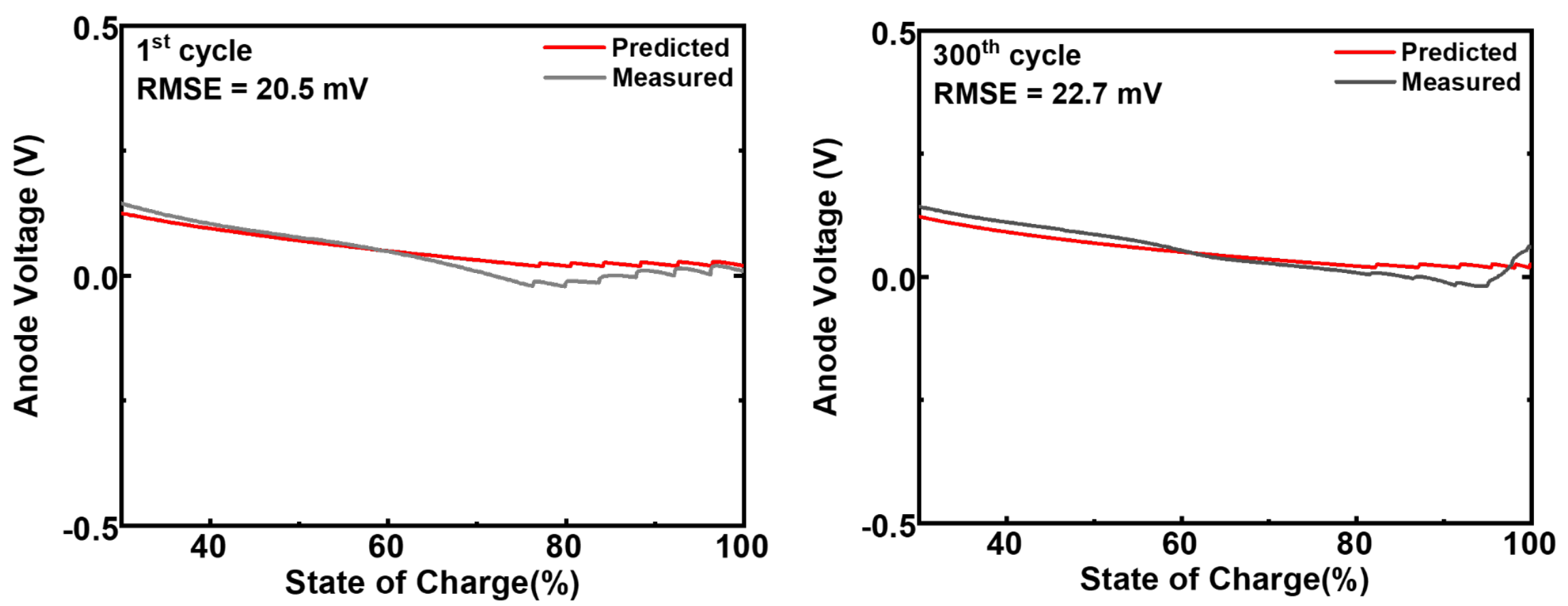

Figure S12. Predicted anode voltage vs Measured anode voltage throughout the life cycle of smart charging protocol, collected from a 3E cell

2. Furthermore, to prove the robustness of our ML model when encountering a charging rate that does not exist in the training set, we trained additional models where we purposefully hold out all cells with specific C-rates during training and then evaluate our model on those cells (**Supplementary Table S4**). These additional models exhibit performance comparable to the base model, demonstrating our robustness in predicting under different C-rate conditions.

Training C-rates	Testing C-rates	# of Training Points	RMSE (V)	MAE (V)
0.33, 0.5, 0.8,	0.33, 0.5, 0.8,	9.79 M	0.023	0.018

1.0, stepped **(Base model)**	1.0, stepped			
0.5, 0.8, 1.0, stepped	0.33	6.92 M	0.022	0.018
0.33, 0.8, 1.0, stepped	0.5	7.36 M	0.023	0.017

Table S4. Robustness of our ensemble NN method in predicting C-rates not existing in training set

Specific Comments R1-5. *In the final verification of smart charging, a 3E battery cell should be used at the same time to verify the accuracy of this method in predicting the anode potential over the entire life cycle***.**

Authors' Response: We appreciate Reviewer #1 for pointing it out. Here a 3E battery cell was used to verify the accuracy of the model over its entire cycle life (**Figure S12**). During our smart charging, there are several steps (0.9C, 0.7C, 0.6C, 0.4C) that are not included in our training set (C/3, C/2, 0.8C, 1C). However, the RMSE error in the last cycle(22.7 mV) is comparable with the beginning of life (20.5 mV), and they are as good as the RMSE of our model on average in Figure 2 (23 mV). That demonstrated the robustness of our model in predicting the conditions not included in the training set.

Manuscript edit:

1. Whereas metallic Li is clearly observed both optically and with SEM in fully discharged CC-CV cells at end-of-life (80% capacity retention) **(Fig. 4f)**, the surface of the graphite anode remains smooth and pristine in smart BMS cells after smart charging **(Fig. 4e & Supplementary Fig. 11)**. At the same time, a 3E cell is applied throughout the cycle life of smart charging protocol to verify the accuracy of this method in predicting the anode potential over the entire life cycle. The high and consistent accuracy between measured and predicted AV verified the robustness of our model while dealing with long-cycled batteries and unseen charging C-rates **(Supplementary Fig. 12)**

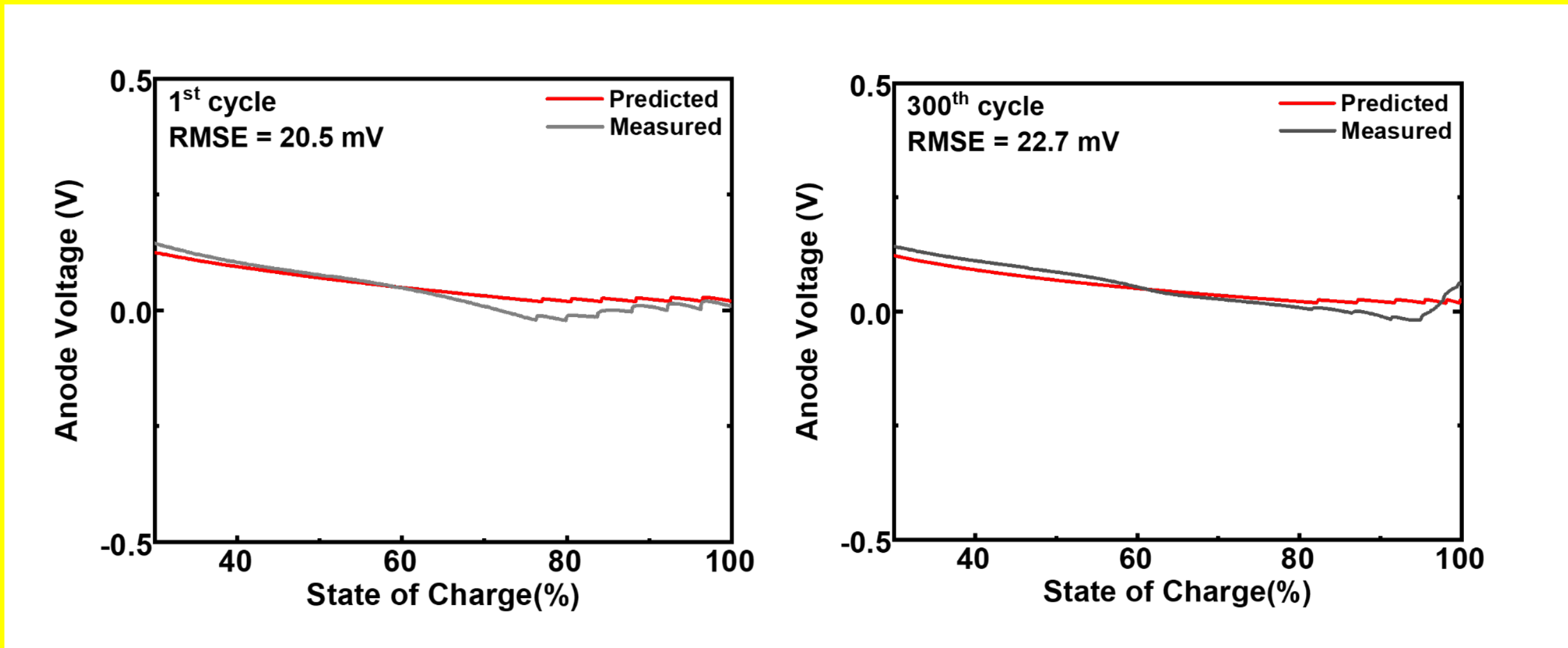

Figure S12. Predicted anode voltage vs Measured anode voltage throughout the life cycle of smart charging protocol, collected from a 3E cell

Response to Reviewer #2

Summary: *This manuscript proposes a virtual sensor to measure anode voltage, for the purpose of mitigating lithium plating at the anode-separator interface. This virtual sensor is, ultimately, an ensemble of 4-layer feedforward neural nets that predict anode voltage from measured voltage and current signals. This machine learning model is trained from data collected from coin cells with reference electrodes.*

The overall idea is intriguing, original from my experience, and has merit. However, the execution of the research needs significant improvements. Comments are categorized below as MAJOR and MINOR::

Authors' Response: We appreciate Reviewer #2 for recognizing the originality of our work and thank the reviewer for the positive feedback on our study. We take this opportunity to respond to each comment in detail below.

Specific Comments R2-1. *[Ensemble Neural Networks] Do all the feedforward neural networks have identical structures and training processes? If yes, then it's unclear why an ensemble approach is used versus simply adding 5X more nodes in the inner layers. Please explain, as the two approaches appear functionally (and perhaps mathematically) equivalent.*

Authors' Response: Yes, the neural networks all have identical structures and training processes. However, the ensemble approach still has unique benefits over a 5X larger network because they have different goals: ensembling 5 networks, where each neural network samples a subset of the training set, is done to increase the generalizability on our dataset. On the one hand, a 5X larger network would increase the complexity of patterns that our neural network could learn. Although this may be desired for a very complex dataset, if there is not enough data, a 5X larger network could easily start to overfit, rendering it useless for prediction and inference. Instead, an ensemble of 5 networks prevents any individual network from overfitting (via sampling from the larger training set), while also improving generalization. This is also supported by the paper ***Kondratyuk, Dan, et al. "When ensembling smaller models is more efficient than single large models." arXiv preprint arXiv:2005.00570 (2020)***. which found an ensemble to be more efficient and performant than a single large model.

Specific Comments R2-2. *[High C-rate Data collection] It seems the highest current loading is 2.37 mA-cm2 and the cells have an areal capacity of 2.37 mAh-cm2. In other words, the data is collected at 1C charging rate and lower. However, many LFP battery applications experience higher C-rates, where lithium plating is a significant risk. For example, the recently publicized*

Zeeker “Golden Brick Battery” – an LFP chemistry – charges at up to 5.5C. Please explain why higher C-rates were not used, which is precisely when the problem of lithium plating is more acute.

Authors’ Response: We appreciate the reviewer’s insightful comment regarding the importance of higher C-rate testing, particularly given the increased risk of lithium plating under fast charging. In this study, we intentionally selected a commercially available bare LFP material with a rate capability of 1C (see **Figure R1**, tested in 2-electrode cells with a **15mm-diameter (thickness: 0.5 mm) Li metal disc** and **10mm-diameter LFP**. Electrolyte applied is basic 1M LiPF6 in EC/DEC (v/v = 50:50) without additives), and paired it with a graphite anode to create a model system with a low N/P ratio (~1.0), which is an aggressive condition for a practical battery system under which to test our approach. These commercial products didn’t cause safety issue, like anode voltage of < 0 V, at C-rates higher than 1 C-rate, and it is hypothesized that as-received *bare* LFP that we purchased has too poor rate capabilities to drive anode voltage into 0 V (In other words, cell potential instantly reaches upper cut-off voltage before anode voltage drops below 0 V at high C-rates), making it difficult to collect sufficient Li plating data points for model training.

As is widely known, LFP is a cathode material with intrinsically low kinetics and poor rate performance. To overcome this drawback, commercial products are commonly enhanced via carbon coating or state-of-the-art electrode engineering techniques, in stark contrast to the bare one that we used in this work.

It is noteworthy that the severely reduced rate capability observed at C-rates above 1C doesn’t stem from issues in cell fabrication. Figure R1 shows not only reproducibility but also capacity recovery when C-rate returned to lower C-rates. Thus, while this limitation reflects the inherent challenge of our current material system, it does not compromise the scientific rigor or impact of our work, as all dataset generation and testing were conducted under C-rates ≤ 1C, where our LFP normally works.

Furthermore, while this material does not support fast charging beyond 1C, **it offered us a platform to investigate early stage lithium plating behavior with 1 C-rate, and allows us to start with a more well-controlled model system that can capture many key features for mechanistic investigation and algorithm development**. Furthermore, through a low N/P ratio, we collected millions of data points with <0 anode voltage, *i.e.,* when Li plating truly happened, which are constructive for our model training, with various but moderate C-rates.

Meanwhile, we fully agree that lithium plating becomes even more severe at higher C-rates, as seen in advanced systems such as the Zeekr “Golden Brick Battery”. Extending our framework to those fast-charging regimes, where the electrochemical environment becomes more

aggressive and the modeling challenge correspondingly increases, is part of our ongoing and future work. We are actively pursuing such extensions by adapting our ML pipeline to fast-charging-capable cells and anticipate that our current work will serve as a solid foundation for those efforts.

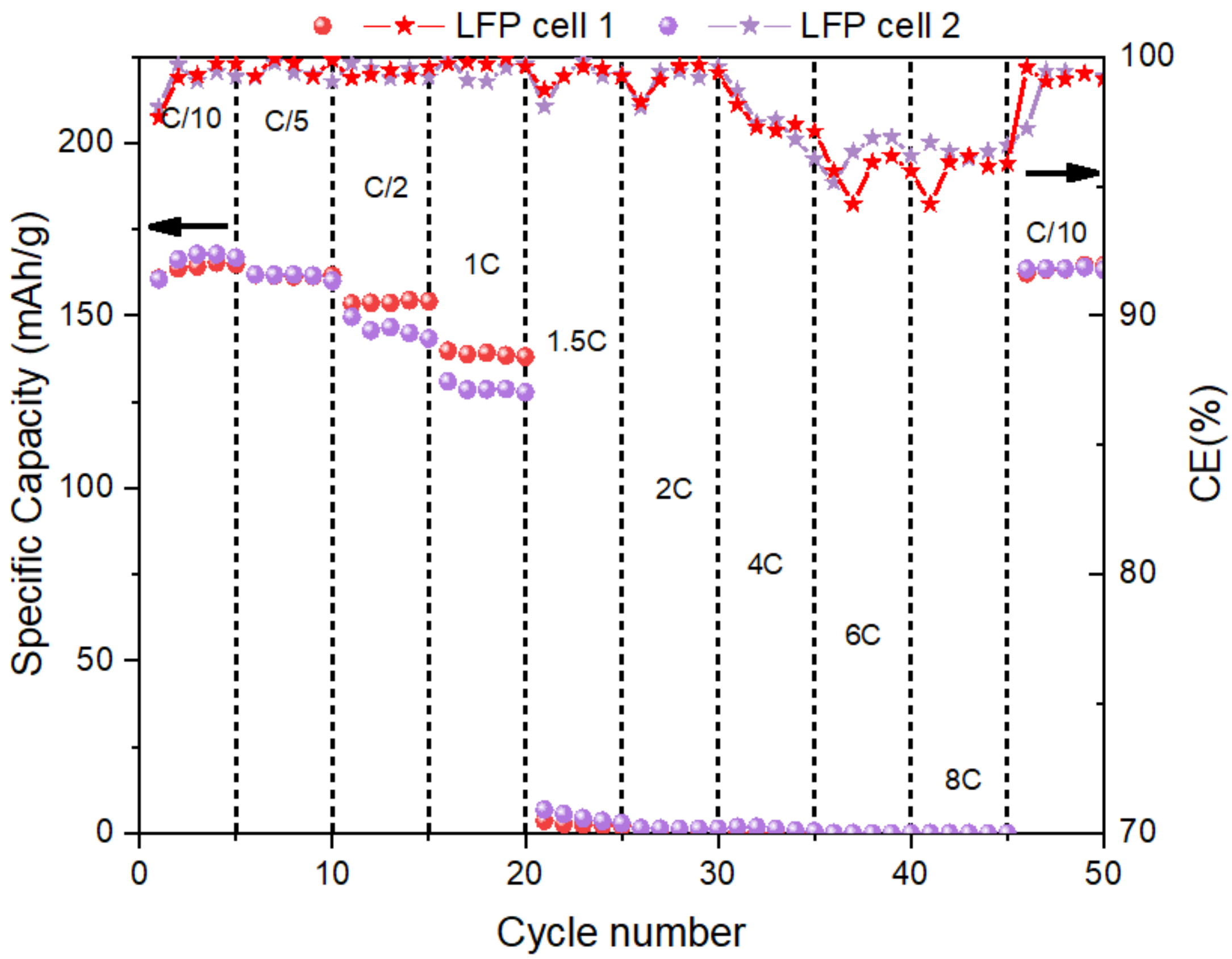

Figure R1. Rate capability test with coulombic efficiency(CE) for our LFP materials measured in Li||LFP half cell, which indicate under C-rates higher than 1C, the specific capacity will become extremely low (<10 mAh/g), 15mm-diameter(thickness: 0.5 mm) Li metal disc and 10mm-diameter LFP electrode are applied as electrodes. Electrolyte: 1M $LiPF_6$ in EC/DEC (v/v = 50:50)

Specific Comments R2-3. *["AI model" versus "ML model" used throughout the paper] The manuscript is quite liberal about using "AI model" and "ML model" interchangeably. This is not only confusing, but misleading. I strongly urge the authors to replace any mention of "AI model" with "ML model". Specifically, an AI model is an algorithm which performs human-like tasks, including reasoning, problem solving, perception, and language understanding. An "ML model" is a specific subclass of AI models focused on learning patterns from data, using statistical and optimization methods. Without question, this manuscript falls in the narrow category of "ML*

models". To be honest, I am irked by the framing of the proposed AV prediction model as "AI" instead of "ML", which gives the impression of salesmanship over scientific rigor.

<u>Authors' Response</u>: We appreciate Reviewer #2 for pointing it out. We have replaced all "AI" with "ML" and highlighted in main text

Specific Comments R2-4. *[Fig. 4d] It is strange that cell voltage is higher for CC-CV versus smart charging – at the same SOC. Can you explain this? Specifically, let's focus on the CC stage for both protocols. Smart charging uses a higher C-rate (2.37 mA-cm2) whereas the CC-CV baseline uses a lower C-rate (2.23 mA-cm2). Assuming the cells are equivalent, I would expect smart charging to have a higher voltage, because of greater ohmic overpotential. However, the opposite happens in Fig. 4d. Please explain.*

<u>Authors' Response</u>: We appreciate Reviewer #2 for the question here. While the smart charging cells and CC-CV cells are operated under the same initial current density settings, **their effective electrochemical environments diverge in a few cycles due to differing degradation behaviors**.

In figure 4d, we present the voltage profile for Cycle #4, whereas Fig. 4c shows that the capacity of CC-CV cells has substantially declined by this point, in contrast to the smart charging cells, which retain much of their initial capacity. This divergence plays a crucial role in interpreting the observed voltage behavior.

Since both groups are charged under the same nominal current density, the absolute current remains fixed. However, at Cycle #4, the actual areal capacity of the CC-CV cells has diminished, which means the C-rate effectively increases (*i.e.*, same current applied to a smaller capacity). This results in higher polarization and overpotential in the CC-CV group. In contrast, the smart charging cells maintain their capacity and therefore operate under more moderate, stable electrochemical conditions despite experiencing higher nominal current densities.

In conclusion, **the CC-CV cells experience relatively harsher kinetic conditions at cycle #4 due to their degraded capacity, which leads to greater overpotential and faster deterioration.**

In the meantime, we truly appreciate this point, as it raises an important perspective that inspires ideas for next research directions on how to build smart BMS that can handle *"effective current issue"* generated by capacity degradation.

Specific Comments R2-5. *[Comparison to Open-Loop Physics models – MOST IMPORTANT COMMENT] Ultimately, the proposed ML algorithm produces a RMSE of 23 mV, which is about*

5% error considering the AV values range from +450 mV to -50mV. Meanwhile, the manuscript claims the proposed ML-powered "virtual sensor" outperforms physics-based models. I request that the authors directly compare their ML model to a state-of-art open-loop physics-based model. Relevant benchmark open-loop models can be found in the following references:
• *https://doi.org/10.1109/TCST.2011.2178604*
• *https://doi.org/10.1109/TCST.2021.3135149*

Specifically, the authors should justify their contribution of ~5% RMSE to be better than a physics-based model. This manuscript is essentially a ML paper, and just like all ML papers, the authors need to compare against the state-of-art benchmarks. I would not expect a lower level of scientific rigor in Nature Communications, and the editorial board should not expect a lower level.

Authors' Response: We appreciate Reviewer #2 for pointing it out. We agree that a direct comparison against state-of-the-art open-loop physics-based models is important to justify the performance of our ML-powered virtual sensor. In the first paper you refer to (***IEEE Transactions on Control System Technology,*** **2013, 21(2)**), the authors compare their prediction with simulated anode voltage, instead of measured AV in 3-electrode cells, for a short period of time (2-3 hours at most). Furthermore, the simulation didn't take aging effect during battery activation cycle, a necessary step in battery cycling into consideration, which will undermine the realistic applicability of this work. The author didn't provide actual simulated anode voltage range so the relative error remains unknown, but we have demonstrated 23 mV error (better than 30 mV reported in this work),from cells throughout their lifetime and from 5 different charging conditions. Our model **stands out in RMSE value, real-world applicability, and variety of train/test sets**, proving the robustness of our model. We would like to point out that a true "virtual reference electrode" should essentially behave like a real physical reference: which means that it should be able to dynamically sense the anode voltage as battery operating parameters change (e.g., current) in real time and maintain this fidelity throughout the lifetime of a battery. To the best of our knowledge, physics-based simulations cannot dynamically predict the anode voltage on the fly (unless a new simulation is run every few seconds to account for the newly updated measured parameters (e.g., current voltage)), or maintain its fidelity throughout the lifetime of a battery. We have highlighted this in our manuscript.

For the second paper (***IEEE Transactions on Control System Technology,*** **2021, 30(5)**), the authors focus on prediction of **cell voltage** and **thermodynamic-related parameters, like temperature.** There is no demonstration of **anode-voltage** prediction so the work focuses on a relatively different area compared with our work. Furthermore, both works mentioned are driven entirely by simulation with no analysis of the degradation mechanisms via characterization or

disassembly; in particular, the first work does not explicitly demonstrate why the cell lifetime improves. Even if the model itself is validated in real-world cells using macroscopic electrochemical data, it is performed in the very first cycle, whereas our approach maintains its prediction accuracy throughout battery lifetime.

Manuscript edit: Added first paper mentioned in introduction part of our manuscript, and added definition of virtual reference electrode:

Although physics-based models that simulate AV based on simplified transport and interfacial kinetics can reproduce early-cycle behavior, they fail to account for dynamic degradation phenomena—such as evolving impedance, lithium inventory loss, and interfacial damage—that span the chemical, thermal, and mechanical heterogeneities over the full lifetime of a battery due to its static or assumed parameter sets. A real physical reference electrode would be expected to be accurate throughout the lifetime of a cell and respond to dynamically changing parameters (e.g., current); any virtual reference electrode should then also have this ability to maintain its prediction accuracy and provide on-the-fly anode voltage sensing capabilities in real time.

Specific Comments R2-6. *[Smart Charging] The proposed smart charging, which experiences chattering, is a strange choice. The natural choice is CC-CAV. That is, once the AV reaches 20mV, for example, then control current to hold this value. This would eliminate chattering and potentially further improve longevity. Can the authors comment on why they applied the chattering approach instead of CC-CAV? Obviously, this is more involved to implement experimentally. But that's a simple engineering problem. I want more explanation beyond "an engineering challenge".*

Authors' Response: We sincerely thank the reviewer for the insightful suggestion regarding the use of a CC-CAV (Constant Current–Constant Anode Voltage) strategy to reduce chattering and improve charging stability. We agree that CC-CAV represents a logical and potentially more effective approach, and we appreciate the opportunity to clarify our reasoning for not implementing it in the current study.

As the reviewer correctly pointed out, the implementation of CC-CAV requires precise control of the charging current in real time to maintain a stable anode voltage. However, in our current setup, the precision for the current control is 0.04 mA. Considering the nominal capacity for our coin cell is 3.18 mAh (1C = 3,18 mA here), the precision of our BMS is about 0.1C. Based on this precision

limitation of our BMS, we applied a stepped charging with 0.1C step size to approximate CC-CAV protocol, which nevertheless showed significant life-cycle improvement as a proof-of-concept and verified the importance of our work. In future work, we plan to address this by upgrading to an advanced BMS equipped with higher-resolution current sensing and control capabilities or test dynamic charging on high-capacity(>100 mAh) pouch cell, which the Arbin BMS is capable of testing, and the current precision will not be a limitation. Additionally, we are exploring the integration of a PID-based current control loop to stabilize the anode voltage during the charging phase, effectively enabling a CC-CAV regime.

Specific Comments R2-7. *[Scalability: From Coin cells to commercial cells] Please comment on the challenges of scaling this approach from single-layer coin cells to commercial cells that could be 50Ah or more.*

Authors' Response: We appreciate Reviewer #2 for asking that. Currently the challenge includes 1) Commercial cells experience non-uniform temperature, local overpotentials, and current density variations, especially at high C-rates or large formats; 2) Commercial cells require richer feature sets: localized temperature, SOC distribution, impedance evolution across layers, tab design, and more; 3) Commercial cells exhibit longer time horizons and more complex dynamics, potentially requiring larger models, online adaptation, or transfer learning to maintain accuracy over the life of the cell. Here we are glad to show some preliminary results we currently have in scalability verification on applying our ML model on prediction of anode voltage measured in Gr|NCM523 3-electrode pouch cells (**Table R1**), which could relieve possible concern on applicability of our model/approach in commercial batteries with higher capacity and different architecture by demonstrating great potential of our base model on predicting different chemistry/architecture.

**Cell type**	**RMSE (V)**
LFP coin cell	0.023
NCM523 pouch cell	0.045

Table R1. Model performance in scalability verification tested on commercial pouch cells equipped with Li reference electrode

To further address the challenges associated with scaling from coin cells with one battery chemistry to large-format commercial cells with various anode/cathode chemistries, we envision leveraging advanced machine learning strategies such as **transfer learning** and **physics-informed neural networks (PINNs)** in future work. Transfer learning will enable us to utilize knowledge gained from well-characterized coin cell data to accelerate model development for larger cells, which are typically more expensive and time-consuming to test. Here we show preliminary results on accurate prediction of AV in NCM811 chemistry applying our LFP model with transfer learning, showing its great potential in model generalization **(Figure R2)**.

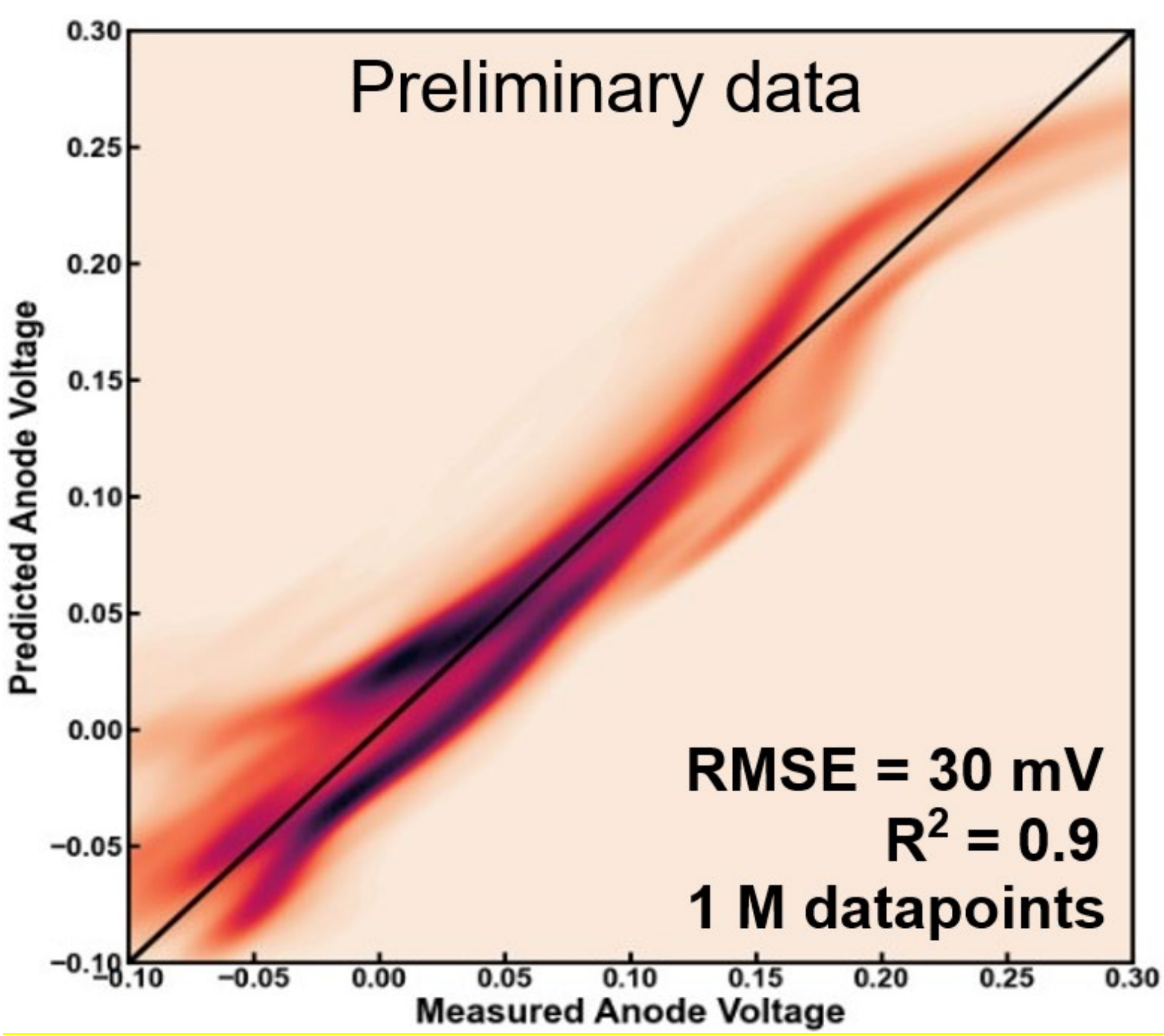

Figure R2. Model performance in scalability verification tested on NCM811 coin cells equipped with Li reference electrode via transfer learning paradigm.

In parallel, PINNs provide a powerful framework to embed electrochemical and thermal physics directly into the learning process, allowing the models to remain accurate and generalizable even when extrapolating to different cell formats and operating conditions. Here we showed the improvement of our model after applying PINN. **In Figure R3, we added physical principles**

(i.e., anode voltage should monotonically decrease during the charging process) and set up a preliminary PINN model. It shows outstanding performance improvement (RMSE = 8 mV). More studies will be explored on optimizing the PINN model and add more physics and electrochemical principles in the future. Together, these techniques will help bridge the gap between small-scale experimental validation and real-world, high-capacity cell implementation with various chemistries, ultimately guiding the rational design and optimization of scalable battery systems.

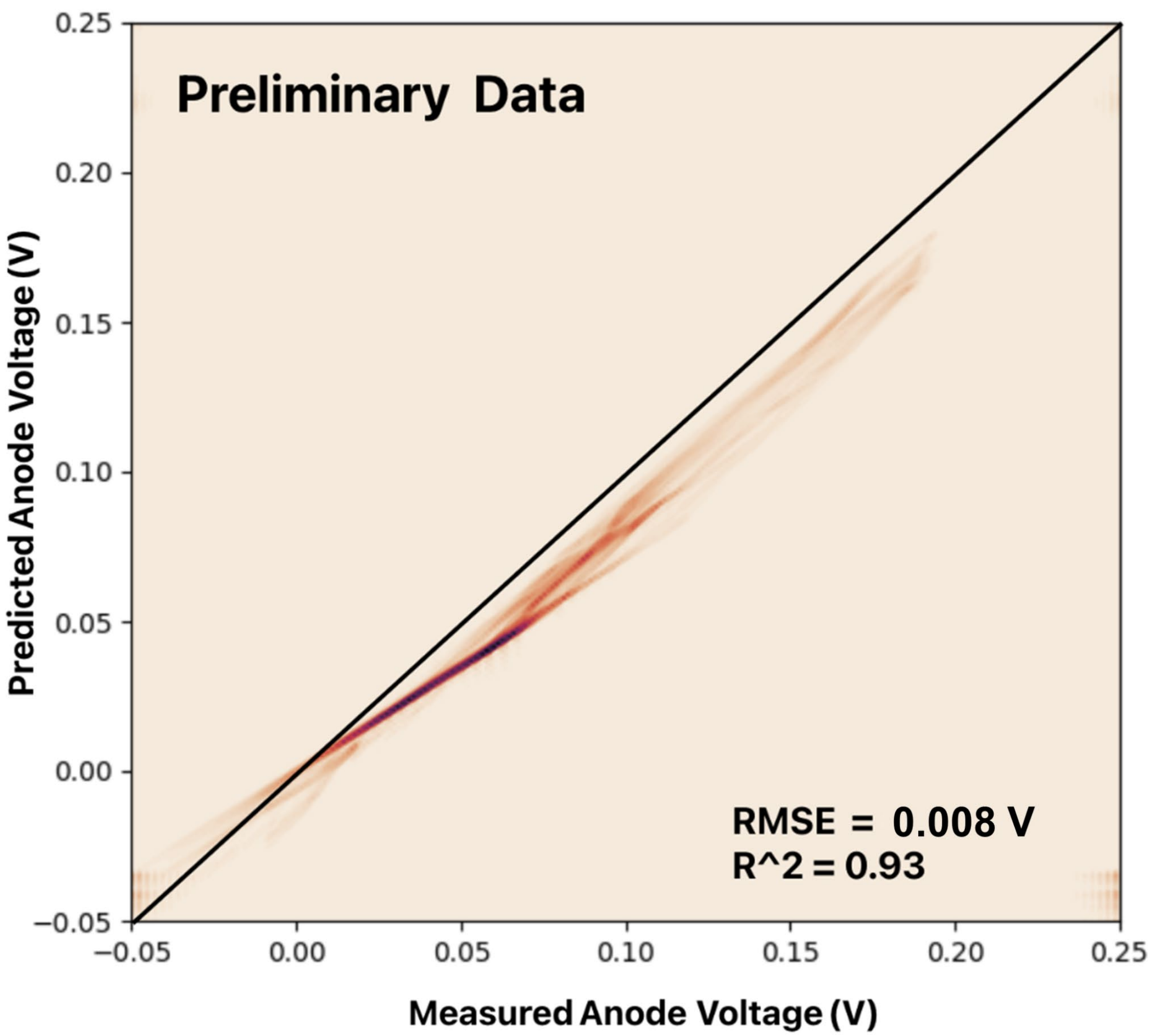

S

Figure R3. Preliminary Data from Physics-Informed Neural Networks trained on LFP dataset.

Specific Comments R2-8. *[Line 57] What are "N/P ratios"? This terminology should be defined. Most of the more general audience of Nature Communications will not know this jargon.*

Authors' Response: We appreciate Reviewer #2 for the suggestion, we added a clear definition of the term "N/P ratio" in the main text when it's first mentioned, and highlighted it.

Manuscript edit: while low N/P ratios (i.e., the ratio of the capacity of the negative electrode (anode) to the positive electrode (cathode)) can enable increased energy density and utilization efficiency of materials

Specific Comments R2-9. *[Line 112] Supplementary Fig. 4 does NOT demonstrate that an ensemble of 5 feedforward NNs provide the highest accuracy. This figure compares linear regression with GPR. Please correct the reference.*

Authors' Response: Thank you for bringing this to our attention. We have updated the reference and have included a new figure, **Supplementary Figure 13** which provides results on how ensembles with fewer models perform. We find that there is a clear trend between more models in the ensemble and a lower RMSE on the test set, showing there is a benefit to having an ensemble of 5 models over just 1 model or a smaller ensemble.

Manuscript edit:

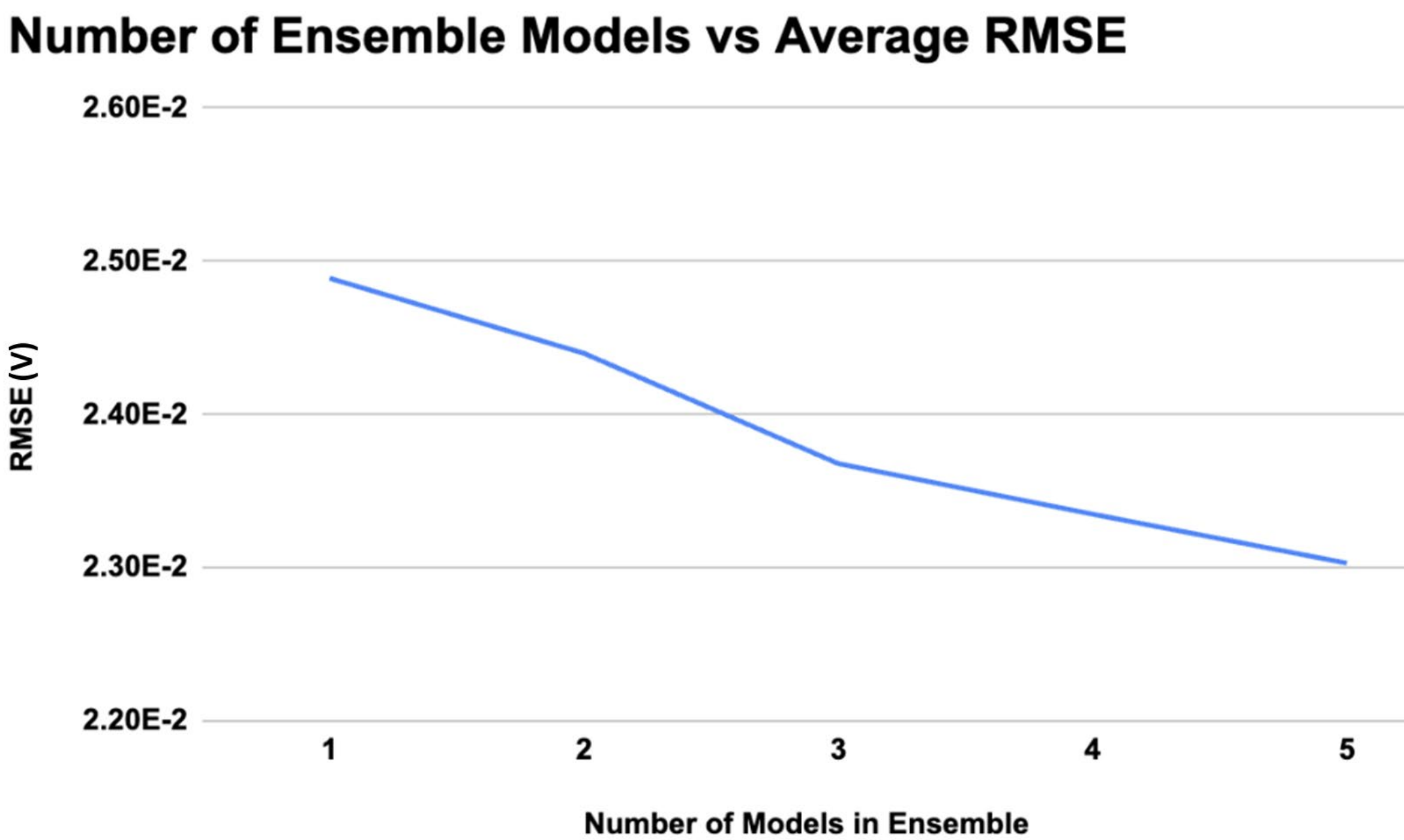

Figure S13. Number of Models in Ensemble vs. RMSE on test set. Results were averaged across multiple runs to reduce noise.

Specific Comments R2-10. *[Line 120] Comparing a 4-layer NN to LLMs is outrageous.Comparing the prediction of the next words (tokens to be exact) is remarkably more complex than predicting the next AV values (which decrease monotonically). Obviously, the training resources for LLMs are orders of magnitude more than those used for the NNs in this paper. Please remove the comparisons with LLMs. It is incredibly distracting to the reader, and provides no substantive value to the paper.*

Authors' Response: We appreciate Reviewer #2 for suggesting. We already deleted the comparison with LLMs.

Specific Comments R2-11. *[Equation for "Energy" feature] Please provide this equation in the supplementary feature. It is a crucial part of the AV prediction model. For clarity and to enable others to reproduce the work, the equation should be included.*

Authors' Response: We appreciate Reviewer #2 for suggesting. We added the definition for "Energy" in Table S3. Note that we used the Energy output from the Arbin software directly, which quantitatively matches the definition we have included.

Manuscript edit:

Table S3. All feature candidates before feature selection process

Feature Category	Feature List
Basic Features	Cell Voltage, Charging Current, Energy*, Capacity, Time, Power
Engineered Features	Throughput, Overpotential, dQ/dV, d(Cell Voltage)/dt, dEnergy/dt, dCapacity/dt, dOverpotential/dt,
Lagged Features ("-Y" means Y seconds ago)	X - 2, X - 4, X - 6, X - 8 (X represents all 13 features in both "Basic Features" and "Engineered Features")

***Energy is defined as positive energy value and calculated by the formula ∫I*V*dt, where I is the charging current, and V is the cell voltage.**

Specific Comments R2-12. *[Line 185] missing a period*

Authors' Response: We appreciate Reviewer #2 for checking our articles carefully. We added the period rightly here.

Specific Comments R2-13. *[Fig. 4a and 4b] Why are the x-axes and y-axes missing values? This is very strange. Please provide, or explain why you are hiding data from the results?*

Authors' Response: We apologize for the confusion caused by the omission of axis values in **Fig.4a and 4b**. Our intention was not to hide any data but to provide a schematic-level comparison between our anode voltage-based control and the conventional cell voltage-based control. These plots were deliberately designed as conceptual illustrations rather than data-driven graphs, in order to highlight the fundamental differences in voltage control strategy and its implication on cell behavior. Actual experimental data was provided in **Fig. 4d**. To avoid any further confusion, in

the revised manuscript, we explicitly label them as schematic diagrams in both the figures, as follows.

Manuscript edit:

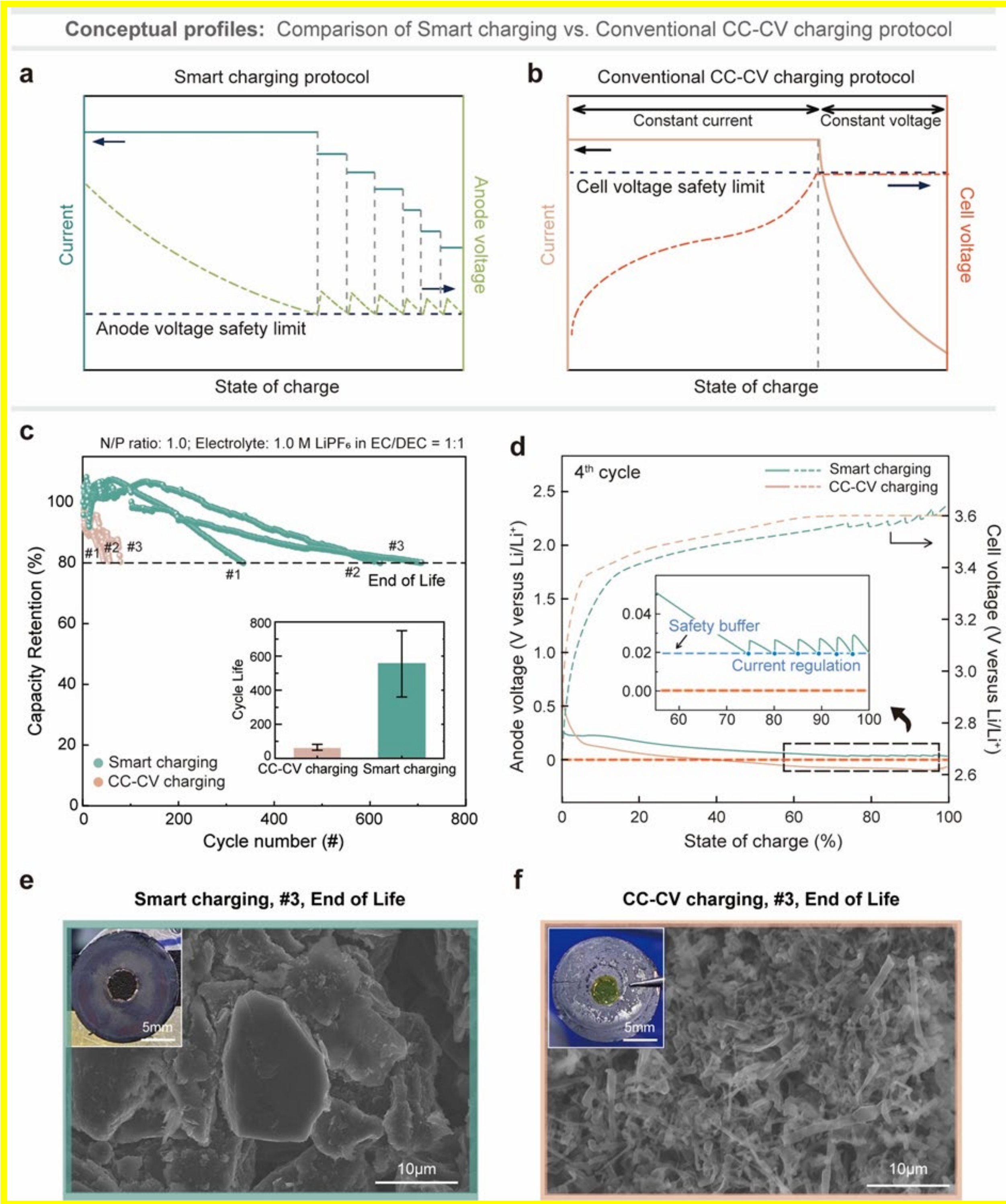

Fig. 4 | A smart charging protocol enabled by our virtual reference electrode. a, b, Conceptual profiles of voltage and current for **(a),** smart charging protocol and **(b),** CC-CV protocol. **c,**

Capacity retention of smart charging versus conventional CC-CV charging. The inset figure shows the average cycle life for both protocols (3 duplicates for each) **d,** Plots of measured AV in CC-CV cell #3 (shown in solid brown line), predicted AV in smart charging cell #3 (shown in solid green line), and cell voltage of both protocols (dashed brown line for CC-CV, and dashed green line for smart charging). **e, f,** SEM images & optical images of the top surface of graphite anode at end-of-life for **(e),** smart charging cell #3**,** and **(f),** CC-CV charging cell #3**.**

Specific Comments R2-14. *[Fig. 4c] It is notable that the baseline CC-CV LFP cell reaches end-of-life at only 100 cycles, considering that high-quality commercial LFP cells have a lifetime of 3000 – 5000 cycles. Can the authors explain this further? The N/P ratio of 1.0 is obviously part of the reason. But is there something else occurring?*

Authors' Response: We thank the reviewer for this important point. Indeed, the early capacity fade observed in the baseline CC-CV LFP cells is notably shorter than the cycle life of high-quality commercial LFP cells. This is primarily due to the intentional design of the test cell as a model system, rather than a fully optimized commercial product. Indeed, it would take many years for lifetime improvement to be quantified in such high-quality commercial cells, which is not our primary purpose. In particular, the N/P ratio was set to 1.0 to simplify interpretation of lithium plating and other degradation mechanisms, even though this configuration is known to reduce long-term stability. Additionally, the anode and cathode formulations were not optimized for high durability; they were selected to accentuate degradation phenomena such as lithium plating for modeling and analysis. This accelerated degradation enabled us to efficiently capture failure signatures and validate our prediction framework within a practical experimental timeframe. For common electrolyte without additives, common commercial electrodes, basic formation protocols. Li plating is just one of many degradation modes, including mechanical cracking of particles, SEI formation & growth, structural disordering of cathode material, etc. *(J. Power Sources., 2017, **341**, 373e386*, shown in **Figure R4**).

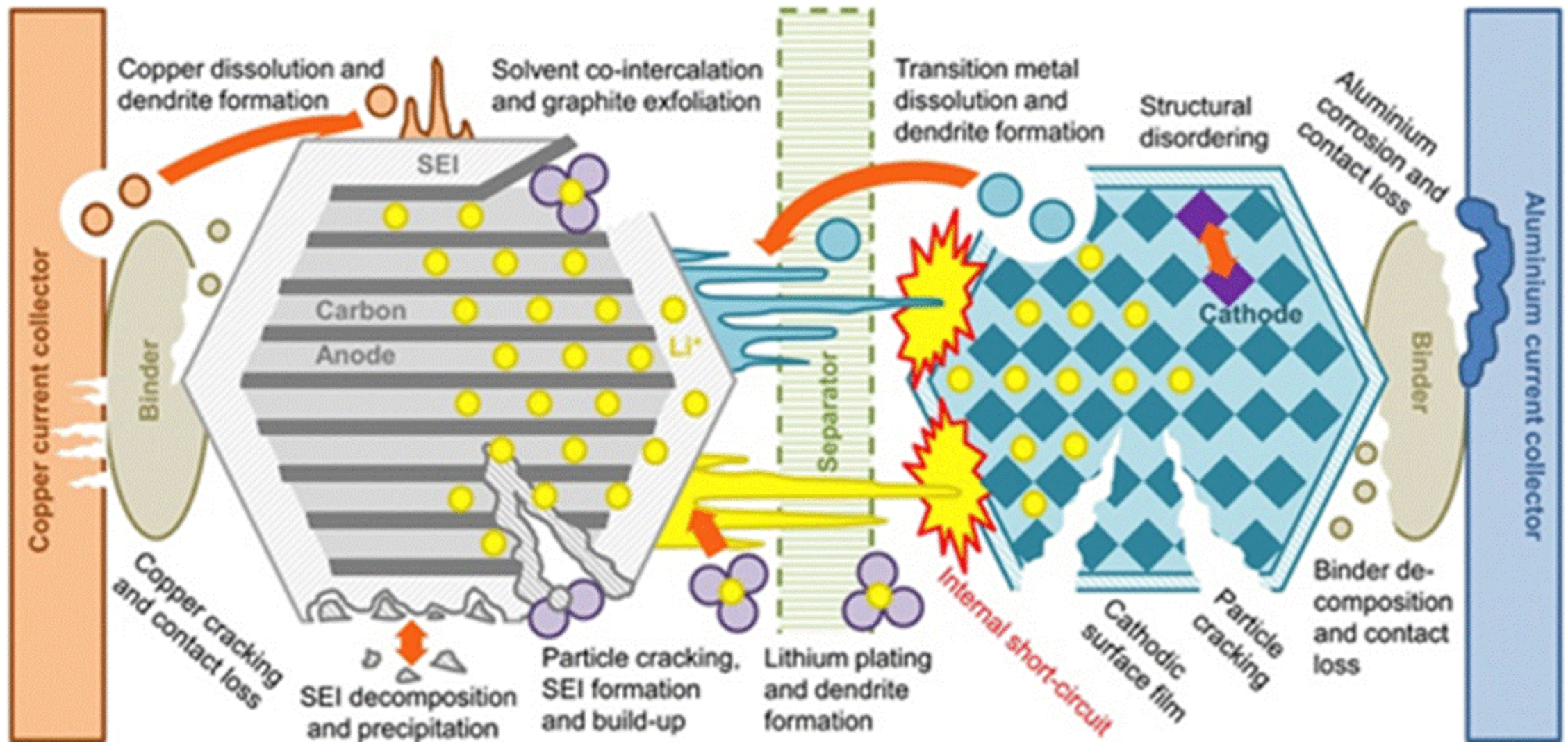

Figure R4. Various degradation mechanisms in Li-ion cells. Reproduced from *J. Power Sources., 2017,* ***341****, 373e386*

Therefore, we expect that smart-charging cells will have reasonable decay resulting from factors other than Li plating, especially considering the cell system is not commercially optimized. And actually, that is something we needed because our ML model should have enough training data in "dangerous" and "decay" situations to give accurate prediction of AV throughout different cycling stages of the battery. And we would like to emphasize that the experimental settings (electrode, electrolyte, temperature) are identical for both protocols, so it is still a fair comparison between them, highlighting the impact that smart charging made on extending battery lifetime in a scenario with high risk of Li plating. We also acknowledge that extending the study to long-life, high-energy commercial-grade cells is an important direction for future validation.

Response to Reviewer #3

Summary: *The authors examine the problem of rapidly charging a lithium ion battery. They argue, convincingly, that this is a very important problem in the literature. Then they point out that battery safety is an issue during fast charging due to lithium plating, which can also compromise battery useful life. Again, this is an excellent point, and it is very well-established in the literature. Then they argue that they can use artificial intelligence to develop a virtual reference electrode. They execute this task, and report an estimation error of 25mV. This allows them to improve battery life during fast charging by a reported 8.5-fold improvement.*

Authors' Response: We appreciate Reviewer #3 for recognizing the originality and impact of our work and thank the reviewer for the positive feedback on our study. We take this opportunity to respond to each comment and suggestion in detail below

Specific Comments R3-1: *"The ideas behind this manuscript are sound. However, the manuscript itself is unfortunately very weak, and should not be accepted for publication in its current form. For one thing, contrary to the authors' main claim, the virtual reference electrode is neither a novel or innovative addition to the literature. I encourage the authors to just go to Google Scholar and type the words "virtual reference electrode" into the search box. They will immediately see work in this area spanning multiple decades. "*

Authors' Response:

As reviewer #3 mentioned, there are past works that include virtual reference electrodes which focus on anode voltage control based on physics-based models. They did provide ways for AV estimation. However, there still exists shortcomings that inhibit the approach from further real-world applications. **First,** although the accuracy of the 1st cycle in these works is good, the models are not validated either in error metrics or with experimental evidence beyond the 1st cycle, which makes the prediction not practical in real-world implementations. This is largely because the aging behavior is so complex (***J. Power Sources.*, 2017, 341, 373e386**, including mechanical cracking, SEI/CEI growth, Li plating, etc) that the parameter set established in fresh batteries will inevitably struggle in late cycles where most parameters' value will drift as the cell ages. We would like to point out that a true "virtual reference electrode" should essentially behave like a real physical reference: which means that it should be able to dynamically sense the anode voltage as battery operating parameters change (e.g., current) in real time and maintain this fidelity throughout the lifetime of a battery. To the best of our knowledge, physics-based simulations cannot dynamically

predict the anode voltage on the fly (unless a new simulation is run every few seconds to account for the newly updated measured parameters (e.g., current voltage)), or maintain its fidelity throughout the lifetime of a battery.. **Second**, past works are dependent on simulated AV signals, where some parameters applied at batteries' microscopic level come from unsubstantiated assumptions (e.g in ***Electrochimica Acta.*, 2021, 395, 139133**, a physics-based model is proposed, where one key parameter applied, the equivalent surface area for electrons to tunnel, is assumed.). Additionally, most PBM didn't take the aging effect of activation cycles, a necessary step in battery cycling into consideration. They only simulate the first cycle at a fresh battery without any activation.(***IEEE Transactions on Control System Technology*, 2013, 21(2)**). Both of the factors will cast further doubt on the real-world impact. **Third**, the validation of AV threshold, as well as SEM/optical image evidence to connect the predicted and observed anode morphology, are absent in past works. In our work, we made several novel improvements to overcome these shortcomings: **1)**: Our data-driven approaches enable accurate prediction of battery health throughout the battery life beyond the 1st cycle. Although it is not commercialized yet, our work sheds light on a brand-new path toward future smart sensors. **2)**: Our research is driven by a large amount of real-world data. To the best of our knowledge, our anode voltage dataset collected with 3E cells is the 1st of its kind. Beyond past works which are dependent on non-real-world simulated data (***Journal of Power Sources 608 (2024) 234620***), our work will have an impact for real-world batteries-related applications. **3)**: We provide clear and solid experimental evidence (not limited to an error metric value), including real-time validation of AV safety threshold, as well as the difference we make by implementing smart-charging protocol (with supplementary experiments, there are 3 reproducible duplicates for each protocol for fair comparison) with AV smart sensing (without physical reference), in order to prove the robustness of the virtual reference electrode. With the smart charging protocol, cycle life of batteries can be improved by 8.25x with comparable or higher average charging speed, which demonstrates our work's potential in facilitating ultra-safe fast charging and screening of defective batteries. As for the fast-charging implementation (10-min charging to 80% SOC), it is not the main focus of our first proof-of-concept paper, but in the future, our work, as a catalyst, will inspire more valuable insights on fast-charging based on AV smart sensing, including application of transfer learning and Physics-informed neural network in improving generality and industrial applicability.

Specific Comments R3-2: *"For another thing, an estimation error of 23mV is not good at all when trying to avoid lithium plating. The over-potentials driving such plating are usually an order of*

magnitude smaller, which means that the reported estimation error may be too high for the purpose of preventing plating."

Authors' Response:

We thank the reviewer for their comment regarding the 23 mV RMSE in our anode voltage prediction model. We agree that further improvement is always desirable. However, we would like to highlight that this performance is already a meaningful advance compared to existing physics-based models. Specifically, traditional physics-based models such as the pseudo-two-dimensional (P2D) and single-particle models (SPM) often exhibit strong predictive accuracy only in the first few cycles (***J. Power Sources.*, 2017, 341, 373e386; *IEEE Transactions on Control System Technology*, 2013, 21(2)**). Their accuracy after the 5th cycle is rarely reported, especially when critical aging phenomena—such as SEI growth, loss of active material, and evolving electrode kinetics during activation—are not explicitly modeled or calibrated. In contrast, our model not only achieves competitive throughout-life accuracy (23 mV RMSE) but also maintains strong generalization across cycles. This reflects our model's ability to implicitly account for degradation dynamics without the need for complex aging parameterization, which is often difficult to calibrate in first-principles frameworks. Furthermore, while physics-based models require detailed knowledge of material and cell-level parameters—which may not be readily accessible in practical settings—our approach offers a scalable, data-driven alternative that delivers robust cycle-to-cycle performance with minimal physical assumptions. In sum, while we recognize room for future improvement, the 23 mV RMSE should be viewed in the context of the enhanced predictive consistency across cycles and the reduced reliance on hard-to-measure parameters. This makes our method not only accurate but also more applicable to real-world cell monitoring and control. By adding a safety barrier (20 mV), we were able to realize outstanding lifetime improvement through real-time anode potential control and dynamic smart charging.

Furthermore, previous studies have reported that Li plating generally exhibits a nucleation overpotential of approximately 100 mV on bare Cu (***Energy Environ. Sci.*, 2024, 17 (18), 6521-6532**) and that of 75 mV on graphite electrodes. (***Energy Environ. Sci.*, 2020, 13, 3723-3731).** These findings strongly support that our RMSE (23 mV) and safety buffer (20 mV) are sufficient to resolve Li plating concerns during battery operation.

To further improve the generalizability and physics interpretability, we are glad to show some preliminary results we currently have for 2 pathways below, and in future studies we

will focus on application of transfer learning as well as Physics-informed Neural network to further push forward the accuracy of AV prediction and we will expect more exciting lifetime improvement through that spiral exploratory process:

1) scalability verification on applying our ML model on prediction of anode voltage measured in Gr|NCM523 3-electrode pouch cells (**Table R1**), which could relieve possible concern on applicability of our model/approach in commercial batteries with higher capacity and different architecture by demonstrating great potential of our base model on predicting different chemistry/architecture;

Cell type	RMSE (V)
LFP coin cell	0.023
NCM523 pouch cell	0.045

Table R1. Model performance in scalability verification tested on commercial pouch cells equipped with Li reference electrode

2) To further address the challenges associated with scaling from coin cells with one battery chemistry to large-format commercial cells with various anode/cathode chemistries, we envision leveraging advanced machine learning strategies such as **transfer learning** and **physics-informed neural networks (PINNs)** in future work. Transfer learning will enable us to utilize knowledge gained from well-characterized coin cell data to accelerate model development for larger cells, which are typically more expensive and time-consuming to test. Here we showed preliminary results on accurate prediction of AV in NCM811 chemistry applying our LFP model with transfer learning, showing its great potential in model generalization **(Figure R2)**. In parallel, PINNs provide a powerful framework to embed electrochemical and thermal physics directly into the learning process, allowing the models to remain accurate and generalizable even when extrapolating to different cell formats and operating conditions. Here we showed the improvement of our model after applying PINN. **In Figure R3, we added physical principles (i.e., anode voltage should monotonically decrease during the charging process) and set up a preliminary PINN model. It shows outstanding performance improvement (RMSE = 8 mV)**. More studies will be explored on optimizing the PINN model and add more physics and electrochemical principles in the future. Together, these techniques will help bridge the gap between small-scale experimental validation and real-world, high-capacity cell implementation

with various chemistries, ultimately guiding the rational design and optimization of scalable battery systems

.

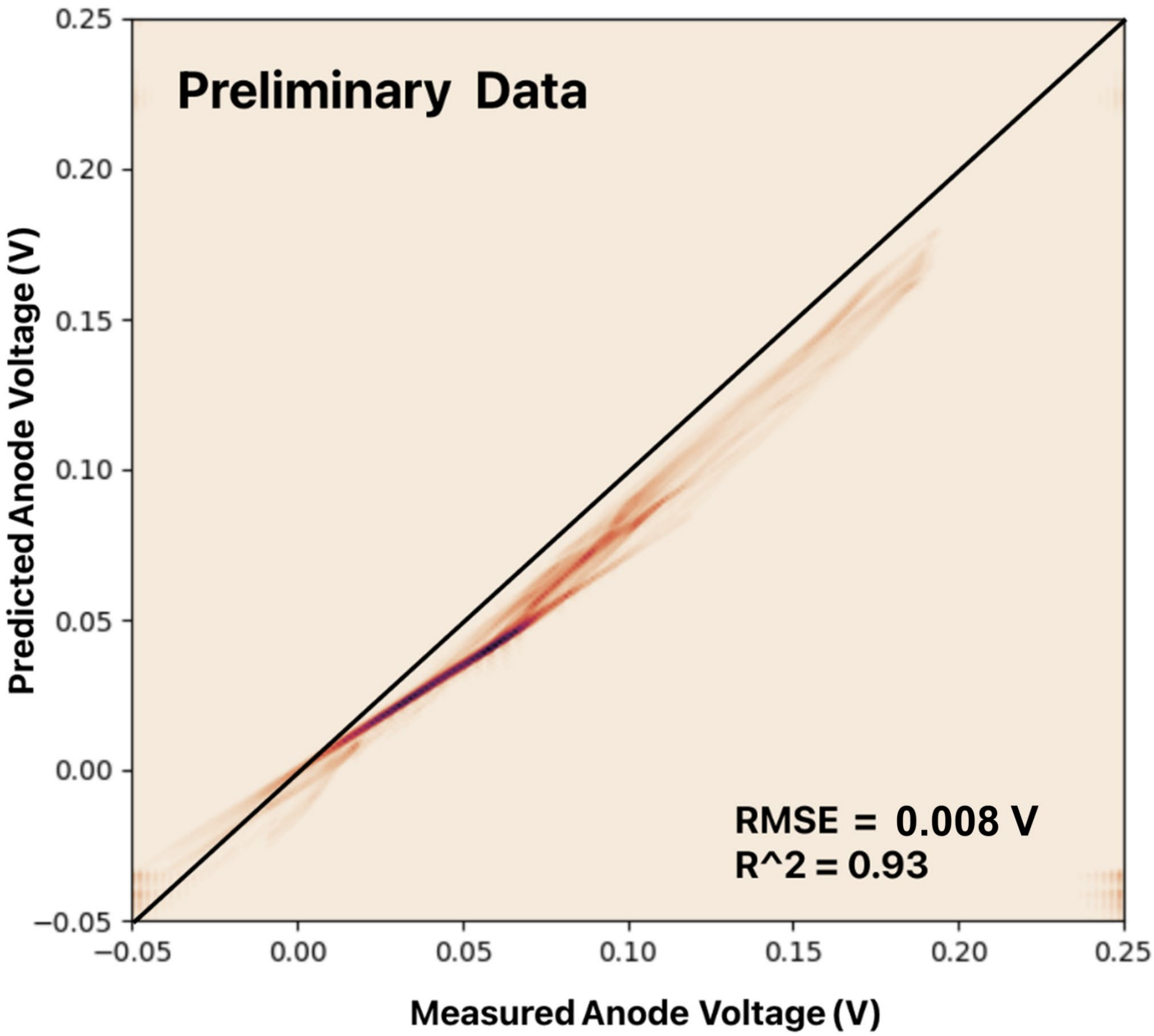

Figure R3. Preliminary Data from Physics-Informed Neural Networks train on LFP data.

Specific Comments R3-3: *"Last but not least, the paper is a little too strong on hype (with too much of the hype being in boldface font), and a little too weak on substance. The ultimate metric for the quality of a paper, in terms of substance, is the degree to which it is written with enough clarity and specificity to the point of being reproducible. As currently written, I doubt that anyone*

can easily reproduce the work in this paper without excessive digging into supplemental materials - and in fact, even with such digging, I am not sure that the work would be truly reproducible. ”

“Perhaps with some very significant rewriting, the paper can become publishable. In particular, I do want to give the authors credit where credit is due by emphasizing the fact that I have not yet seen an ML-based virtual reference electrode in the literature, and have not yet seen a credible, scholarly, hype-free comparison of such an ML-based virtual reference electrode versus a physics-based virtual reference electrodes. Those particular elements of the work may indeed be novel, and I would be excited to see them in publication. One challenge I see with this work is its excessive emphasis on hype at the expense of emphasizing the more thoughtful elements of the work. Hopefully the authors can revise the work with this feedback in mind.”

Authors' Response:

We sincerely thank Reviewer #3 for recognizing the impact of our work, and your suggestions on rewriting and your concerns on reproducibility.

According to your sincere suggestions, we did significant rewriting of the manuscript, including: **1)** Rewrote the entire “background” part (Please kindly read main text Paragraph 1-3), removed all unnecessarily **highlighted** terms that remains over-hype concern, including comparison between our ML model with ChatGpt, as well as replace all “AI” with “ML” (we highlighted “”ML” in manuscript with yellow);

Manuscript edit: ~~Considering our relatively small dataset compared to those used to train foundation models (e.g., large language models like ChatGPT[40]), the high prediction accuracy exhibited by our virtual reference electrode is quite promising with room for even further improvement.~~

2) Clarified the difference we make in advance to previous works on “partial virtual reference electrode” in **response to comment 3-1**
3) Added clarification of battery items (e.g. N/P ratio) when it first shows, as well as subtitles for each divided chapter to make the article more understandable.

Manuscript edit: while low N/P ratios (i.e., the ratio of the capacity of the negative electrode (anode) to the positive electrode (cathode)) can enable increased energy density and utilization efficiency of materials

4) Provided supplementary **data/tables/figures (Added Fig. R1-R4, Fig. S12-S13, Table R1, , and Table S4, Revised Table S3)** and to demonstrate the robustness of our model in predicting C-rates throughout cycles that's not provided in the training set, the advantage of our model against physics-based models, and the potential of our data-driven approach towards broader battery chemistries and architectures.

Last but not least, we will make our raw dataset, code for model training and dynamic charging available to the public upon acceptance of our manuscript. We are confident that everyone with our open-source information for materials and code can reproduce our results.

Response Letter to Reviewers' Comments

Manuscript ID: NCOMMS-25-27724A

Title: Battery smart sensing via a virtual reference electrode

Authors: Jiayi Yu, Aki Takahashi, Min-Ho Kim, Rishi Upadhyay, Howard Zhang, Tianyu Wang, Huayang Zhu, Robert Kee, Tyrone Vincent, Bo Liu, Xintong Yuan, Thomas Hymel, Kaiyan Liang, Keyue Liang, Haoyang Wu, Dingyi Zhao, Jung Tae Kim, Achuta Kadambi✉, Yuzhang Li✉

Response to Reviewer #1

Summary: *The topic of this study is very interesting. However, unfortunately, the following three points still make me feel that this manuscript does not meet the strict requirements of Nature Communications:*

Authors' Response: We appreciate Reviewer #1 for recognizing the novelty of our work and thank the reviewer for the positive feedback on our study. We take this opportunity to respond to each comment in detail below.

Specific Comments R1-1. *The current estimation results are based on a large number of experiments conducted on coin cells. The authors have not yet clearly explained how this work can be applied to commercial high-capacity batteries, as it is difficult to obtain such a large amount of anode-voltage data in commercial batteries.*

Authors' Response: We appreciate Reviewer #1 for pointing it out. The applicability to commercial high-capacity batteries is indeed very important, but we would like to emphasize that our research paper aims to provide **a proof-of-concept for machine-learning-driven virtual reference electrodes** based on real-world experimental data instead of simulated data. Our model can accurately predict anode voltage without the need for a physical reference electrode—a capability that is especially valuable given that traditional physics-based models often fall short in capturing the complex, nonlinear and time-evolving behaviors of real batteries.

Although our initial validation was conducted in coin cell configuration—a widely used and controlled benchmark platform in lab-scale battery research, we fully recognize the importance of demonstrating scalability of our method. We thank the reviewer for raising the commercial batteries demonstration. We would like to clarify again that our study specifically aims to "a proof-of-concept for machine-learning-driven virtual reference electrodes based on real-world experimental data instead of simulated data". The topic of demonstration on commercial batteries is certainly important, but it pertains to a different domain that is outside the intended scope of this work. With that being said, recently we explored the protocol of assembling 3-electrode pouch cells **(Figure R1)**, and we are glad to show some preliminary results training our ML model on anode voltage measured in our **Gr|NCM523 3-electrode pouch cells dataset** (Including 1.5 M cells from 4 different charging rates, **Table R1**). The performance on NCM 523 pouch cells (RMSE = 0.036 V) is almost comparable with LFP coin cells within the same order of magnitude. Although the limitation on the datapoint we have collected on pouch cells exists, it can be reasonably improved by a larger dataset in the future. And we believe that these preliminary results help address concerns on applicability of our model/approach in commercial batteries with higher capacity and different architecture (e.g. pouch cell, cylindrical cell, prismatic cell), and relative follow-up results will be in our next separate publication. In future work, we aim to integrate transfer learning with a large-language-model–inspired positional encoding architecture to develop a universal anode-voltage diagnosis model (i.e., 'Battery-GPT') applicable to mainstream cell architectures and electrode chemistries (e.g. LFP, NCM811, NCM523, etc). We believe that the present proof-of-concept study provides a solid foundation for this long-term vision.

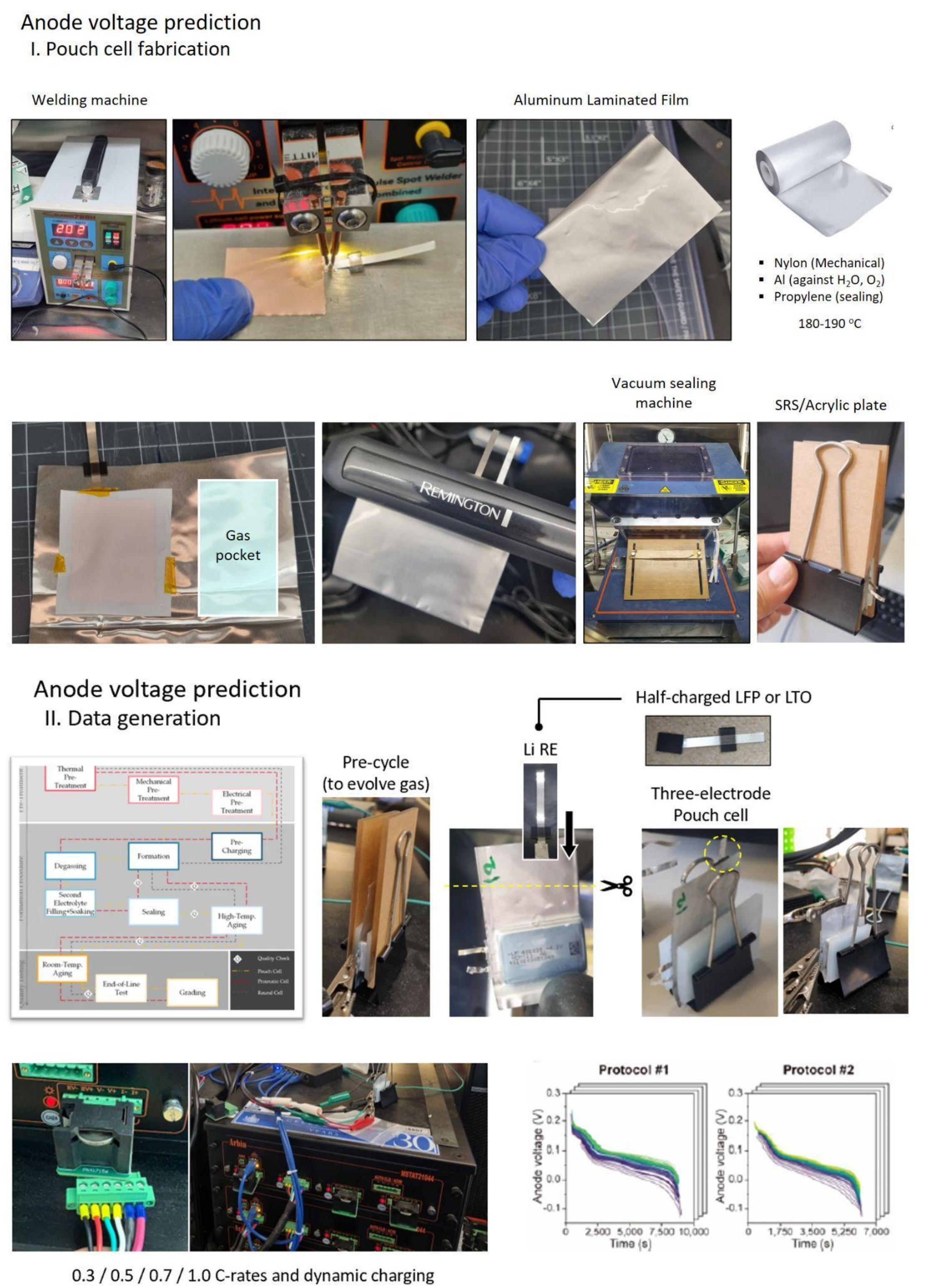

Figure. R1: Assembly and cycling of 3-electrode pouch cell for measuring AV in industrial system

Cell type	RMSE (V)
LFP coin cell	0.023 (with 10 M data points)
NCM523 pouch cell	0.036 (with 1.5 M data points)

Table R1. Model performance in scalability verification tested on commercial pouch cells equipped with Li reference electrode

Specific Comments R1-2. *The anode potential is highly sensitive to charging rates and testing environments. The current estimation error is, in my opinion, too large to meet application requirements. As shown in Figure S12, the measured anode potential is already below 0 V near 80% SOC, which will directly trigger lithium plating, while the predicted anode potential remains above 0 V, failing to provide warning.*

Authors' Response: We thank Reviewer #1 for this comment. For **Figure S12**, the minimum anode voltage measured for two subfigures are respectively -0.015 V and -0.012 V, which are below 0 V. The reason is the safety threshold (20 mV) set here is based on **mean** absolute error (MAE) tested in 8 cells in test set, but in real-time prediction, at some timepoints, the |measured AV - predicted AV| value will be larger than 20 mV, which could result in negative anode voltage. However, the negative anode voltage period is relatively short (~5.6 mins) during a complete dynamic charging process. Furthermore, it is demonstrated in **Fig. 2b**(shown below) that under a large sample (2 M datapoints) broad view, the majority situation (the dark red area) is that the predicted anode voltage will be lower than measured anode voltage. The "overpredicted" phenomenon in **Fig. S12** is likely to be a special case with low probability, but it is still possible since the 2D density of "overprediction" area is not strictly equal to zero In spite of that, what we would like to demonstrate here is that through applying a relatively low safety threshold, we realized 8.25 X lifecycle improvement without sacrificing, but increasing the charging speed, while the predictability of our model won't decay over cycle. We agree that applying a higher safety buffer will help avoid the occurrence of this rare and short negative anode voltage period, but it will also decrease the charging speed. The main focus of our demonstration here is proving the long-cycle prediction capability of our model without any physical reference electrode and parameterization-like steps in physics-based model, and much better cyclability due to the application of our virtual reference electrode under an unfavorable N/P ratio (~1) without sacrificing any charging rate. They are all constructive towards the safe-fast-charging for next-

generation batteries in the future. Future study will be performed on design of charging protocol, including the effect of safety threshold for optimizing the balance between charging rate and anode voltage.

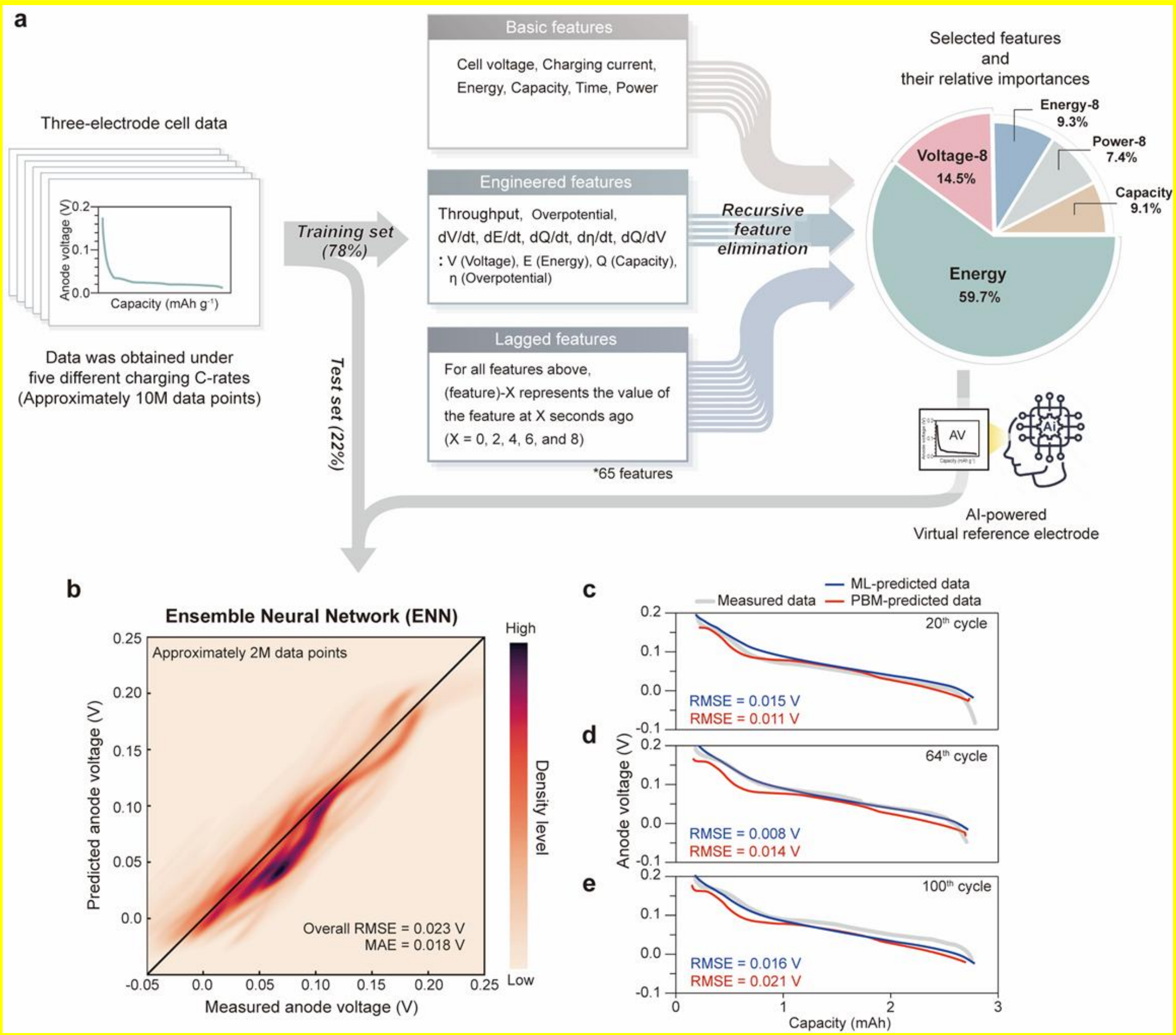

Fig. 2 | Feature selection workflow and overall performance of virtual reference. a, Workflow of feature engineering and selection. 3E cell data was first split into training and test sets (78% / 22%), then 65 features were created according to battery-domain knowledge. Finally, 5 features were selected via RF-RFE. **b,** 2D density plot of predicted AV versus measured AV across all cells in test set; **c-e**, Predicted and measured AV comparison at **(c)**, cycle 20 **(d)**, cycle 64 (best

individual-cycle performance) and **(e)**, cycle 100 of cell #2 in test set. Measured AV, ML-predicted AV, and PBM-predicted AV are shown in grey, blue, and red respectively.

Specific Comments R1-3. *Ultra-fast charging is an inevitable trend. The 1 C rate mentioned in the manuscript is insufficient to provide further guidance for researchers in this field. Research on lithium plating should instead focus on high-rate or low-temperature conditions.*

Authors' Response: We thank the reviewer for this insightful comment. We fully agree that ultra-fast charging and low-temperature conditions represent important and practical regimes for studying lithium plating. However, the current work is intended as a first proof-of-concept demonstration of our neural-network-driven virtual reference electrode framework, aiming to establish and validate its fundamental accuracy under controlled baseline lab-experimental conditions (1 C, room temperature). This rate was deliberately chosen to match the rate capability of electrode materials we have, allowing us to isolate and verify the method's core predictive capability with limited resources.

Building on this foundation, we are currently extending the same methodology to ultra-fast charging(~6C) and low-temperature regimes(e.g. 0℃, 10℃, etc.), which will be presented in a separate, dedicated publication. We believe this stepwise approach—first demonstrating conceptual validity, then applying to more extreme use cases—ensures scientific rigor and clarity of contribution.

Response to Reviewer #2

Summary: *I appreciate the authors' thorough efforts to address my comments. The effort is impressive. Meanwhile, I wish to reiterate that the proposed idea is novel and impactful, which is why I am motivated to assist in the review process. Also I acknowledge that some comments may not seem directly relevant to the conclusions. However, they are relevant to readers with my expertise. Thank you for considering them. All that said, I am still unsatisfied with one response.*

<u>Authors' Response</u>: We appreciate Reviewer #2 for professional feedback and patience. We take this opportunity to fairly compare our approach directly with a physics-based model in this revised manuscript.

Specific Comments R2-1. *R2-5 was my MOST important comment, because it is crucial to the manuscript's claimed contributions. However, the authors are avoiding a direct comparison to a physics-based model. I ask again, please perform a comparative analysis, instead of shrugging off the suggestions as irrelevant. The authors claim that 23mV RMSE is better than state-of-art physics-based models. If this is their claim, then they must PROVE it, no? Specifically, they can and should do the following:*

Indeed, most existing physics-based models do not adapt their parameters to reflect aging, and this would introduce errors in predicting AV. Great! This gives the authors' approach an inherent advantage! The key question is this - which has greater error, physics-based models (with parameters that do not adapt) or the proposed ML approach? If the authors reject my suggestions for a physics-based model comparison, then they can select the one that is most appropriate in their view.

Measured AV via a 3rd reference electrode is obviously the best physics-based prediction. But installing a 3rd reference electrode is not practical in commercial applications. Meanwhile, the state-of-art competing technology would be a physics-based state observer using control theory. This is the gold standard. A simple physics-based state observer is an open-loop observer, which is simply a prediction model. This prediction model will be imperfect, in some sense. For example, the parameters may not adapt to aging. But this approach is still the gold standard. This is why I am insistent on comparing the key contribution to the gold standard / state-of-art approach.

technology would be a physics-based state observer using control theory. This is the gold standard. A simple physics-based state observer is an open-loop observer, which is simply a prediction model. This prediction model will be imperfect, in some sense. For example, the parameters may

not adapt to aging. But this approach is still the gold standard. This is why I am insistent on comparing the key contribution to the gold standard / state-of-art approach.

Authors' Response: We thank the reviewer for the constructive comments. In this revised manuscript, to ensure a fair comparison between our ML approach and the physics-based approach, we collaborated with a research group in Colorado Schools of Mine, which is experienced in physics-based models for Li-ion batteries, and applied AV prediction via a P2D model specifically tailored for our 3E coin cell system.

To be specific, The physics-based model used in the present study is a pseudo-two-dimensional (P2D) model that has been developed and documented by Zhu, et al [Zhu, H. et al. *Journal of The Electrochemical Society* **171**, 050512 (2024)]. However, the model is tailored to the physical characteristics and materials that represent the present coin cells. Where available, materials properties are taken from published literature. The open-circuit potential for the Li_xFePO_4 cathode is taken from Safari and Delacourt [Safari, M. & Delacourt, C. *Journal of The Electrochemical Society* **158**, A562 (2011)] and the open-circuit potential for the graphite anode is based on Verbrugge et al [Verbrugge, M., Baker, D., Koch, B., Xiao, X. & Gu, W. *Journal of The Electrochemical Society* **164**, E3243 (2017)]. The ion conductivity, diffusion coefficient, lithium-ion transference number, and the thermodynamic enhancement factor for the electrolyte $LiPF_6$ in EC:DEC (1:1 w:w) are based on measurements by Lundgren et al [Lundgren, H., Behm, M. & Lindbergh, G. *Journal of The Electrochemical Society* **162**, A413 (2015).]. Once these properties and functions are fixed, other parameters within the model are adjusted to fit measured transient voltage-current behaviors. Measured voltage and current are available at intervals of $\Delta t = 1$ s, over the entire charge-discharge experiments for many cycles. The model is tuned to represent the measured cell voltage-current behavior. The major fitting parameters include the exchange current densities and symmetric factors in Butler–Volmer charge-transfer rate, as well as the nominal electrode particle size and lithium diffusion coefficient within the particles.

As shown in revised **Fig.2c-e**(shown below), for our ML approach, the single cycle RMSE at **cycle 20** (**0.015 V**, the blue curve in **Fig. 2c**) is nearly identical to that of **cycle 100** (0.016 V, the blue curve in **Fig. 2e**), underscoring the consistency of our model over long-term cycling.

For PBMs, the prediction error at **cycle 20** is comparable to that of the ML model and even slightly lower (**0.011 V**; red curve in **Fig. 2c**). However, as the battery ages, the error progressively increases and eventually exceeds that of our ML model (**0.014 V** at **cycle 64** and **0.021 V** at **cycle 100**; red curves in **Fig. 2d,e**). This trend highlights the advantage of the ML approach in forecasting long-term degradation phenomena using real-world battery datasets that inherently capture diverse aging behaviors.

With all that being said, we do not want to argue that ML models are superior to physics-based models (PBMs). Instead, we agree that they are complementary, and we already revised the manuscript accordingly to avoid overclaiming and mislead. On the one hand, with a large and diverse real-world dataset, our ML approach demonstrated edge on predicting anode voltage throughout battery life with complex degradation behavior, which used to be a major defect on PBMs since it's computation- and labor-intensive to fit numerous parameters account for dynamic degradation phenomena—such as evolving impedance, lithium inventory loss, and interfacial damage—that span the chemical, thermal, and mechanical heterogeneities over the full lifetime of a battery due to its static or assumed parameter sets. Considering the accuracy of such physics-based approaches in predicting AV throughout long-term cycling is rarely validated with experimental evidence or visualized graphically beyond the 5th cycle before [Wassiliadis, N., Kriegler, J., Gamra, K.A. & Lienkamp, M. *Journal of Power Sources* **561**, 232586 (2023); Lu, Y. et al. *Journal of Power Sources* **513**, 230529 (2021); Ren, Y., Widanage, D. & Marco, J. *Batteries* **9**, 294 (2023); Konz, Z.M., Weddle, P.J., Gasper, P., McCloskey, B.D. & Colclasure, A.M. *ACS Energy Letters* **8**, 4069-4077 (2023)], our dataset with various cycling conditions and whole battery lifecycle, will be highly valuable for quantitative validation and calibration of next-generation PBMs. On the other hand, the knowledge acquired from PBMs can also be beneficial to assisting and extending the physical interpretation of ML algorithms, and physics-informed neural network (PINN), which hybrids both approaches, has been proved effective improvement on battery parameter prediction [Wang, F., Zhai, Z., Zhao, Z., Di, Y. & Chen, X. *Nature Communications* **15**, 4332 (2024).]. While it's not the main aspect of the present study, we are currently working on advanced PINN development on predicting AV on different battery architectures and electrode chemistries(e.g. LFP, NCM811, NCM523, etc), and relative follow-up results will be in our next separate publication

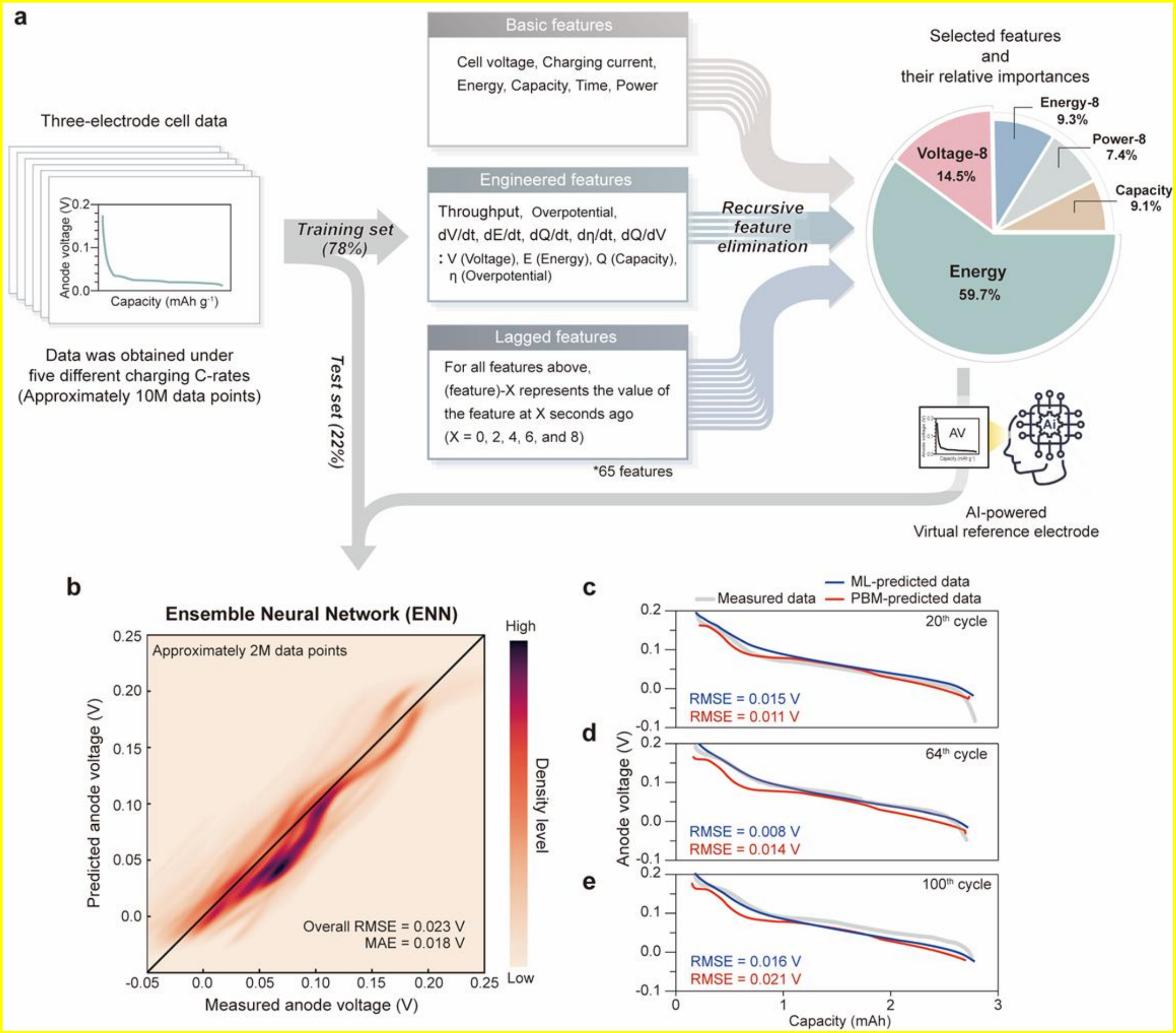

Fig. 2 | Feature selection workflow and overall performance of virtual reference. a, Workflow of feature engineering and selection. 3E cell data was first split into training and test sets (78% / 22%), then 65 features were created according to battery-domain knowledge. Finally, 5 features were selected via RF-RFE. **b,** 2D density plot of predicted AV versus measured AV across all cells in test set; **c-e**, Predicted and measured AV comparison at **(c)**, cycle 20 **(d)**, cycle 64 (best individual-cycle performance) and **(e)**, cycle 100 of cell #2 in test set. Measured AV, ML-predicted AV, and PBM-predicted AV are shown in grey, blue, and red respectively.

Manuscript edits:

Page 4, Line 28: Physics-based models (PBMs) provide an alternative means to predict and interpret AV behavior. For example, pseudo-two-dimensional (P2D) models[47] are formulated to solve systems of conservation equations that represent relevant electrochemical transport and kinetics processes within a battery. Numerous parameters and functional relationships must be established to represent particular cell materials and microstructures. Some properties, such as electrolyte-solvent conductivity, can be measured and characterized via independent experiments. Other characteristics (e.g., charge-transfer rates, effective composite-electrode conductivity) must be derived from measured battery performance (e.g., charge-discharge polarization, electrochemical impedance spectroscopy EIS, galvanostatic intermittent titration technique, i.e. GITT). Typical practice in current literature establishes parameters based on fresh cells, thus not including cumulative degradation processes over hundreds of charge-discharge cycles. Nevertheless, degradation behaviors (e.g., SEI growth, interfacial resistances, electrode-particle fracture, loss of lithium inventory) can be included into physics-based models. However, doing so quantitatively requires experimental evidence to establish needed functions and parameters. A real physical reference electrode would be expected to be accurate throughout the lifetime of a cell and respond to dynamically changing parameters (e.g., current). Thus, a virtual reference electrode should therefore offer comparable fidelity, maintaining prediction accuracy while enabling real-time, on-the-fly estimation of anode voltage throughout operation.

Page 7, Line 112: Beyond the scope of this work, the dataset also provides a valuable foundation for long-term quantitative calibration and validation of underlying PBMs.

Page 8, Line 139: To enable a fair comparison between the ML and physics-based approaches, we implemented AV prediction using a P2D model calibrated specifically to our 3E coin-cell system (**Fig. 2c-e** & Methods in supplementary information)

Page 8, Line 145: For PBMs, the prediction error at cycle 20 is comparable to, and even slightly lower than, that of the ML model (0.011 V; red curve in **Fig. 2c**). However, as the battery ages, the error progressively increases and eventually exceeds that of our ML model (0.014 V at cycle 64 and 0.021 V at cycle 100; red curves in **Fig. 2d,e**). This trend highlights the advantage of the

ML approach in forecasting long-term degradation phenomena using real-world battery datasets that inherently capture diverse aging behaviors.

Page 9, Line 158: While this study primarily introduces the conceptual framework of a virtual reference electrode rather than optimizing its accuracy, we emphasize that data-driven ML approaches will continue to benefit from expanded datasets, computational resources, and in-depth physical interpretation from PBMs as well, as they provide complementary strengths for battery modeling.

Revised Figure 2:

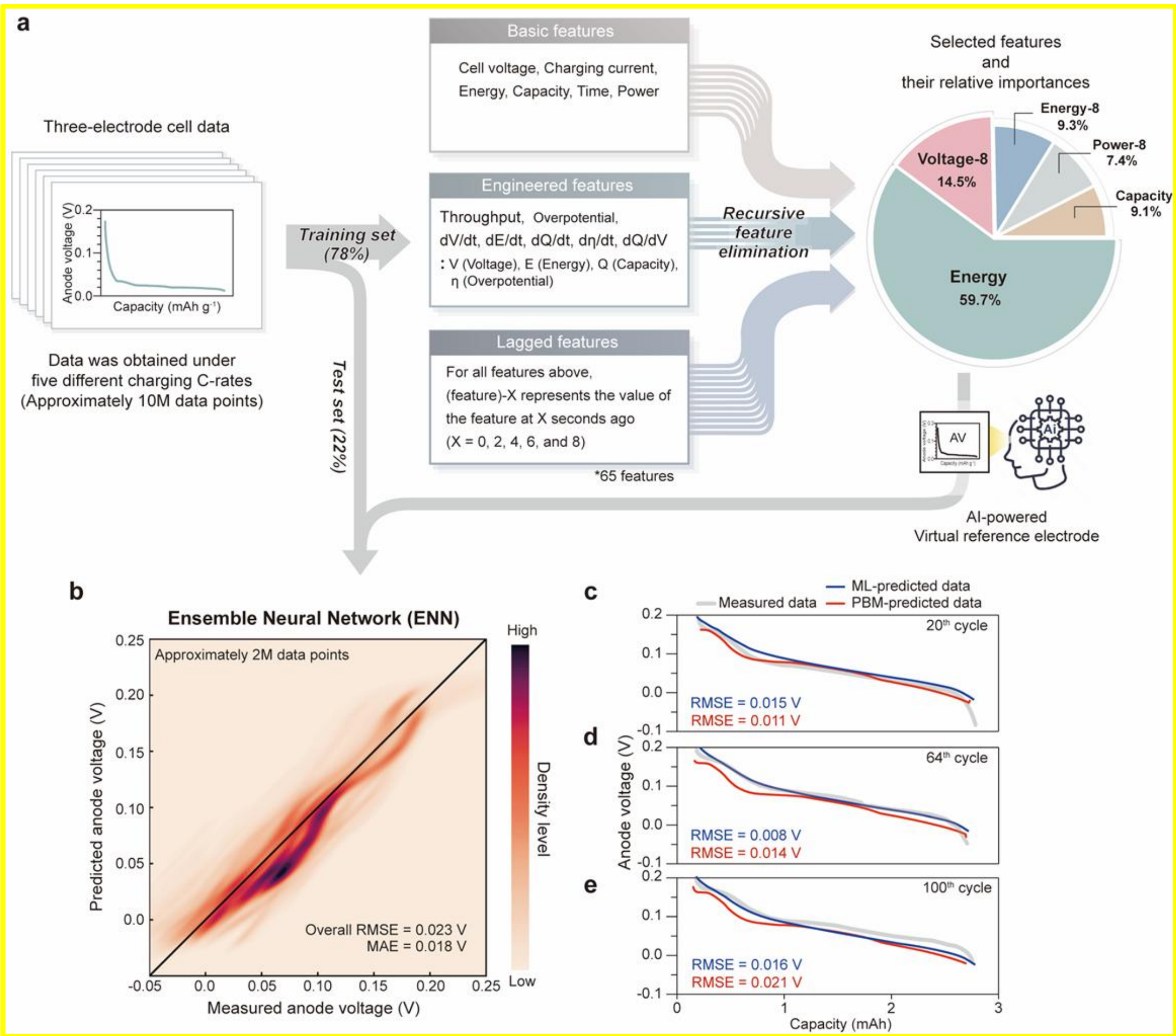

Fig. 2 | Feature selection workflow and overall performance of virtual reference. a, Workflow of feature engineering and selection. 3E cell data was first split into training and test sets (78% / 22%), then 65 features were created according to battery-domain knowledge. Finally, 5 features were selected via RF-RFE. **b,** 2D density plot of predicted AV versus measured AV across all cells in test set; **c-e**, Predicted and measured AV comparison at **(c)**, cycle 20 **(d)**, cycle 64 (best individual-cycle performance) and **(e)**, cycle 100 of cell #2 in test set. Measured AV, ML-predicted AV, and PBM-predicted AV are shown in grey, blue, and red respectively.

Support information-Page 7:

Physics-based simulation on anode voltage

The physics-based model used in the present study is a pseudo-two-dimensional (P2D) model that has been developed and documented by Zhu, et al[13]. However, the model is tailored to the physical characteristics and materials that represent the present coin cells. Where available, materials properties are taken from published literature. The open-circuit potential for the Li_xFePO_4 cathode is taken from Safari and Delacourt[14] and the open-circuit potential for the graphite anode is based on Verbrugge et al[15]. The ion conductivity, diffusion coefficient, lithium-ion transference number, and the thermodynamic enhancement factor for the electrolyte $LiPF_6$ in EC:DEC (1:1 w:w) are based on measurements by Lundgren et al[16]. Once these properties and functions are fixed, other parameters within the model are adjusted to fit measured transient voltage-current behaviors. Measured voltage and current are available at intervals of $\Delta t = 1$ s, over the entire charge-discharge experiments for many cycles. The model is tuned to represent the measured cell voltage-current behavior. The major fitting parameters include the exchange current densities and symmetric factors in Butler–Volmer charge-transfer rate, as well as the nominal electrode particle size and lithium diffusion coefficient within the particles.

Response Letter to Reviewers' Comments

Manuscript ID: NCOMMS-25-27724B-Z

Title: Battery smart sensing via a virtual reference electrode

Authors: Jiayi Yu, Aki Takahashi, Min-Ho Kim, Rishi Upadhyay, Howard Zhang, Tian-Yu Wang, Huayang Zhu, Robert Kee, Tyrone Vincent, Bo Liu, Xintong Yuan, Thomas Hymel, Kaiyan Liang, Keyue Liang, Haoyang Wu, Dingyi Zhao, Jung Tae Kim, Achuta Kadambi✉, Yuzhang Li✉

Response to Reviewer #1

Specific Comments R1-1. *On page 8, the test dataset was directly used in the comparison of results for different models (see Supplementary Fig. 13), and the authors ultimately chose an ensemble of five feedforward neural networks. However, I think that the test dataset should not be used during the model selection process, as this can contaminate the model and lead to overfitting. I believe that the number of models in ensemble also falls under the category of hyperparameters.*

Authors' Response: We thank the reviewer for this careful observation and agree that the test dataset should not be used for hyperparameter tuning or model selection, including the choice of ensemble size.

In our study, the test dataset was **not** used during model training or hyperparameter optimization (e.g., network architecture, learning rate, regularization). During hyperparameter tuning, 20% of the training set was randomly selected as a temporary validation set, and all tuning was performed exclusively using these training and validation datasets. The ensemble size was selected a priori based on robustness and variance-reduction considerations, rather than optimized to maximize test-set performance.

Supplementary Fig. S13 was included to illustrate the general trend of performance stabilization with increasing ensemble size on a held-out test set, as a post hoc analysis of generalization

behavior. We acknowledge that, as originally presented, this figure could be interpreted as using the test set for ensemble-size selection.

To address this concern and avoid ambiguity, we have revised the Supplementary Information to explicitly clarify that the test dataset was used only for final performance evaluation

We have updated the figure caption and corresponding text to clearly state this separation, ensuring that the evaluation protocol adheres to best practices and avoids test-set leakage.

Manuscript edit:

Supplementary information, **Page 6-7:**

We considered hidden units of size 64, 128, 256, and 512, learning rates 0.1, 0.01, 0.001, dropout rates of 0.2, 0.3, and 0.5, batch size 32, 64, 128, and 256, and 1-5 models for ensemble size. For hyperparameter tuning, the training set was further split (80/20) into a smaller training set and validation set used to pick between hyper-parameters. Hyperparameter tuning was done using the Keras Tuner framework.

Supplementary information, **Page 17:**

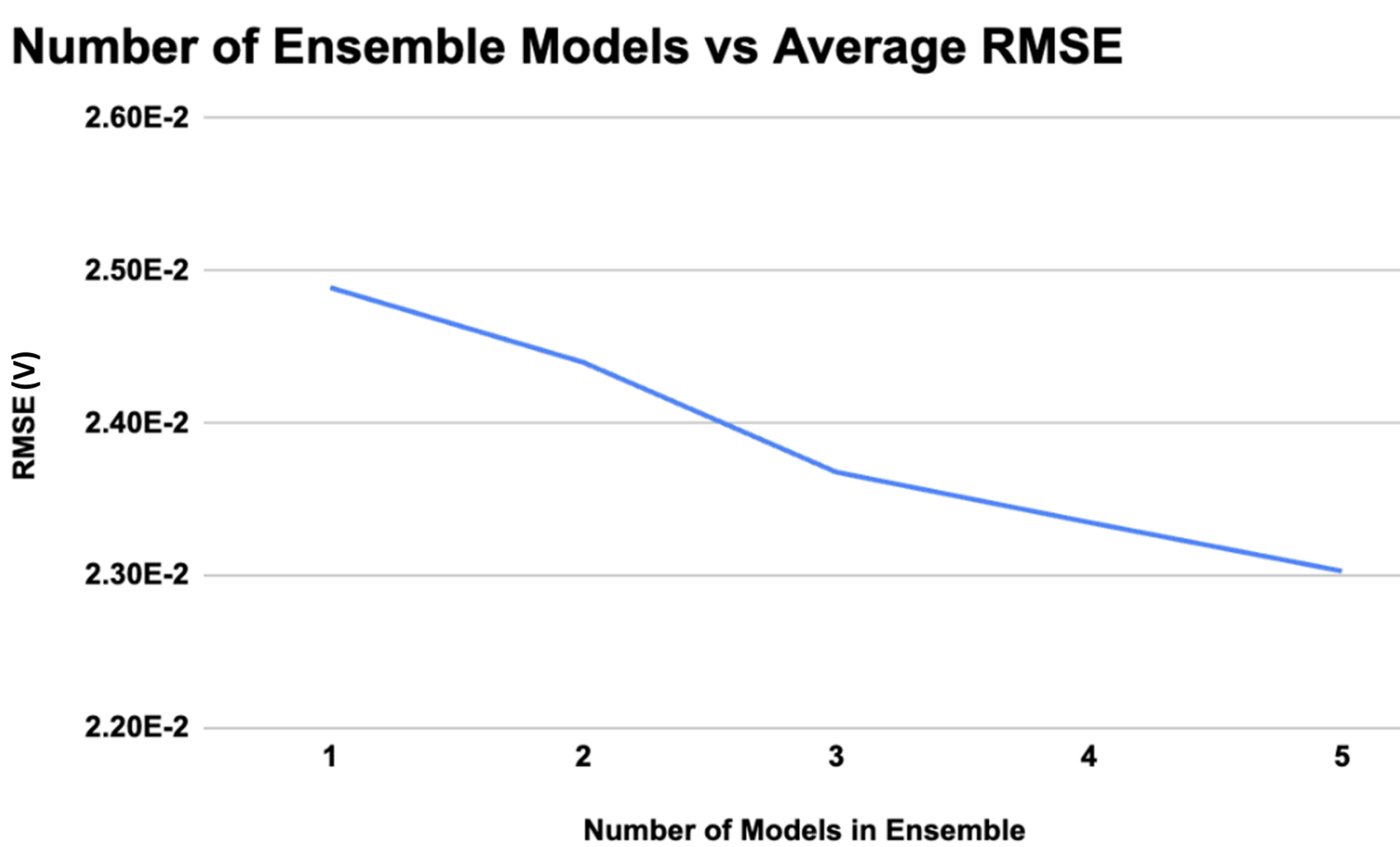

Figure S13. Number of Models in Ensemble vs. RMSE on test set. Results were averaged across multiple runs to reduce noise, as a post hoc analysis of generalization behavior

Specific Comments R1-2. *When the proposed ensemble model is trained, are the five neural networks trained individually, or are they trained as a whole based on the average of their predictions?*

Authors' Response: We thank the reviewer for this clarification request.

The ensemble consists of five neural networks trained individually, each with identical architectures and hyperparameters but different random initializations. Each network produces an individual prediction of the anode voltage, and the final reported anode voltage is obtained by averaging the five independent outputs during inference. The networks are not trained jointly, and no loss function is defined on the ensemble-averaged prediction.

This clarification has been added to the Methods section to explicitly describe the ensemble training and inference procedure.

Manuscript edit:

Supplementary information, **Page 6:**

Five models are trained independently, and the predictions of the five models are then averaged to improve overall accuracy and reduce uncertainty.

Specific Comments R1-3. *Table S4 illustrates the robustness of the ensemble NN method in predicting C-rates not existing in the training set. Considering that this model will mainly be used to regulate charging strategies in the future, is it feasible to use 0.33 C, 0.5 C, 0.8 C, and 1.0 C as the training set and stepped protocol as the test set? Or, what is the prediction accuracy of the neural network in the entire process of smart charging protocol (which I believe is also a new stepped condition not present in the training set) shown in Fig. 4? The relevant results may be very convincing for the promotion and application of this network.*

Authors' Response:We thank the reviewer for this insightful comment regarding the generalization of the ensemble neural network to stepped charging protocols and its applicability to practical smart-charging regulation.

To directly address this question, we performed an additional evaluation in which all constant C-rate data (0.33 C, 0.5 C, 0.8 C, and 1.0 C) were used for training, while cells operated under stepped charging protocols were reserved exclusively for testing. Under this setting, the ensemble neural network achieved a test RMSE of approximately 38 mV in predicting anode voltage (**Figure R1**).

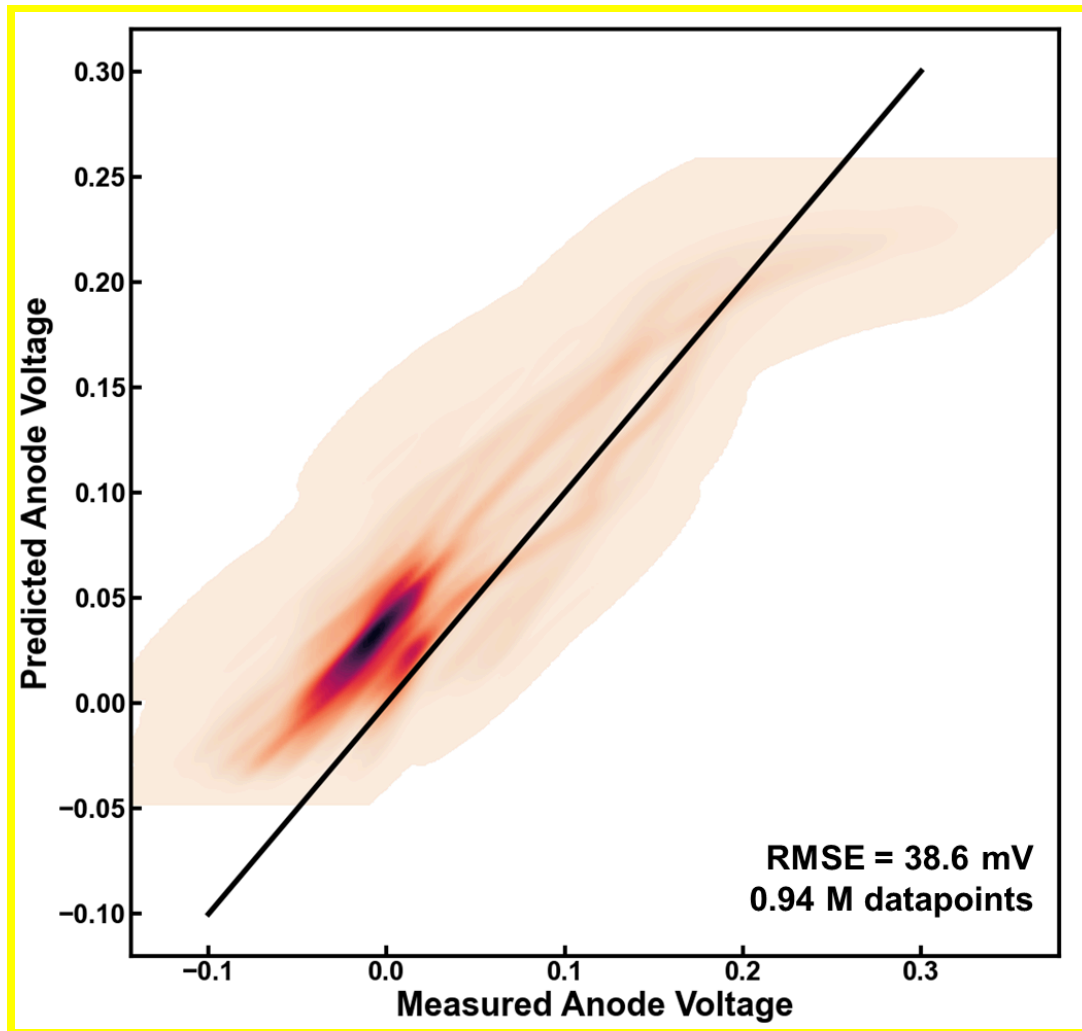

Figure R1. Model performance while training on CC group but test on stepped group

The modest performance degradation(from 23 mV to 38 mV) relative to constant-rate testing reflects the increased difficulty of extrapolating from stationary constant-current conditions to dynamically varying charging protocols, rather than data leakage. Several factors may contribute to this effect, including: 1) distribution shift, as stepped charging introduces current transients and operating regimes not present in the constant-rate training data; (2) dynamic and history-dependent effects, since stepped protocols induce non-monotonic overpotential trajectories; and (3) error accumulation over long sequences, as prediction errors may propagate across multi-stage charging profiles. Such effects are expected when generalizing from constant-rate conditions to time-varying charging strategies.

Instead, to evaluate performance under practical smart-charging operation, **we further validated the model using a three-electrode (3E) cell operated under the smart-charging protocol throughout its cycle life**. Across the entire charging process and over long-term cycling, the predicted anode voltage remains in high and consistent agreement with the measured reference-electrode signal, despite the use of unseen and dynamically varying charging currents. These results, shown in **Supplementary Fig. 12**, demonstrate the

robustness of the proposed method when applied to long-cycled batteries and charging protocols not present in the training set.

Moreover, these results indicate that including a limited amount of stepped-charging data during training—despite differences from the smart-charging profiles—can further improve prediction accuracy under practical smart-charging conditions, reinforcing the suitability of the proposed ensemble model for real-world charging control applications.

Supplementary information, Page 17:

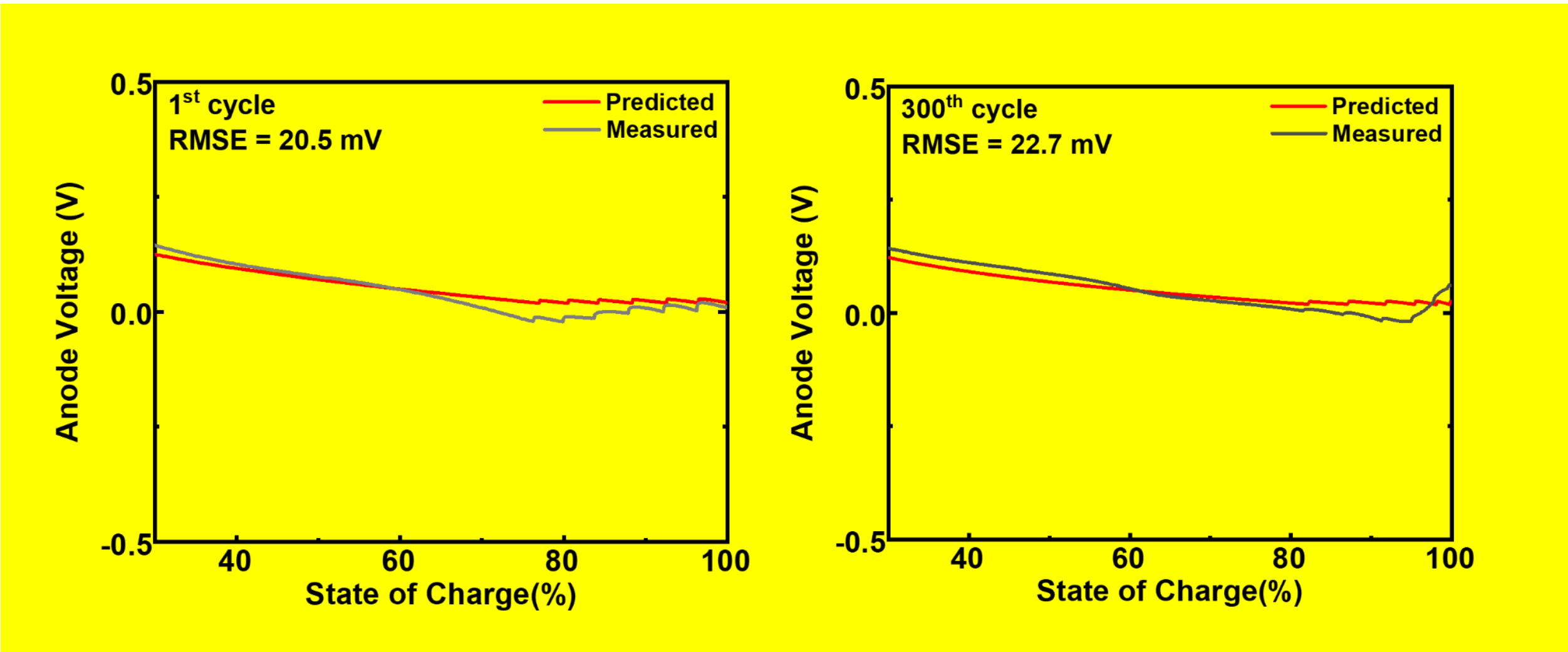

Figure S12. Predicted anode voltage vs Measured anode voltage throughout the life cycle of smart charging protocol, collected from a 3E cell

Response to Reviewer #4

Specific Comments R4-1. *You split 78/22 by the number of per-second measurements. Please clarify whether different cycles from the same cell can appear in both train and test, which would cause time-series leakage and inflate generalization performance.*

Authors' Response: We thank the reviewer for raising this important point regarding potential time-series leakage. Although the 78/22 split is reported in terms of datapoint number, the data partitioning is performed strictly at the cell level. All cycles from a given cell are assigned exclusively to either the training set or the test set **(Table S2)**. Consequently, no cycle-level or time-series data from the same cell appear in both training and test datasets, and no temporal information is shared across the split. This cell-wise partitioning strategy ensures that the reported performance reflects true generalization to unseen cells, rather than interpolation across cycles from the same cell. We have clarified this point in the Methods section to avoid ambiguity.

Manuscript edit:

Supplementary information, Page 23:

*Data partitioning is performed strictly at the cell level. All cycles from a given cell are assigned exclusively to either the training set or the test set. Therefore, no cycle-level or time-series data from the same cell appear in both training and test datasets, and no temporal information is shared across the split.

Specific Comments R4-2. *The SI states you removed CC data below 3.3 V and all CV data. Real BMS charging often includes CV or multi-stage strategies. How will the model behave when CV/low-voltage segments are present, and how do you avoid deployment distribution shift caused by this exclusion?*

Authors' Response: We thank the reviewer for this important question regarding the exclusion of low-voltage and constant-voltage (CV) segments and the implications for practical deployment.

To directly address this concern, we performed an additional training and evaluation in which the model was retrained using the full voltage domain (2.0-3.6 V) and including

CV-stage data. Under this setting, the ensemble neural network achieved an RMSE of 30.3 mV with an R^2 of 0.898, demonstrating that the proposed approach remains robust even when low-voltage and CV segments are included. These results are now provided in the Supplementary Information using a two-dimensional density plot between predicted and measured anode voltage **(Figure S14).**

The exclusion of CC data below 3.3 V and CV-stage data in the main analysis was therefore not a limitation of the model, but a deliberate design choice motivated by the experimental conditions studied. In the present coin-cell system operated at relatively low C-rates, low cell voltages and CV stages correspond to reduced lithium-plating risk due to the lower current and diminished overpotential. Excluding these segments allows the model to focus on learning patterns associated with high plating-risk operating conditions, which are most relevant for smart-charging control and early plating detection.

Regarding deployment and distribution shift, the additional full-domain training results confirm that the model can generalize when CV and low-voltage segments are present. Moreover, in our follow-up work involving more aggressive cycling conditions (e.g., low temperature and high C-rates up to 6-8 C), lithium-plating risk during low-voltage and CV stages is expected to increase substantially. In such regimes, we will explicitly track model performance across the entire voltage domain, including CV and low-voltage segments.

Together, these results demonstrate that the proposed framework is robust to the inclusion of CV and low-voltage data, while the selective exclusion used in this study reflects a risk-focused modeling strategy rather than a constraint on applicability.

Manuscript edit:

Supplementary information, **Page 6:**

Although low-voltage data (<3.3 V) and constant-voltage (CV) stage data were not included in the primary training dataset used in the main text, to demonstrate the robustness of our approach, an ensemble neural network was additionally trained using the full voltage domain (2.0–3.6 V), including CV-stage data, and performance is shown in **Supplementary Fig.14**.

Supplementary information, Page 18:

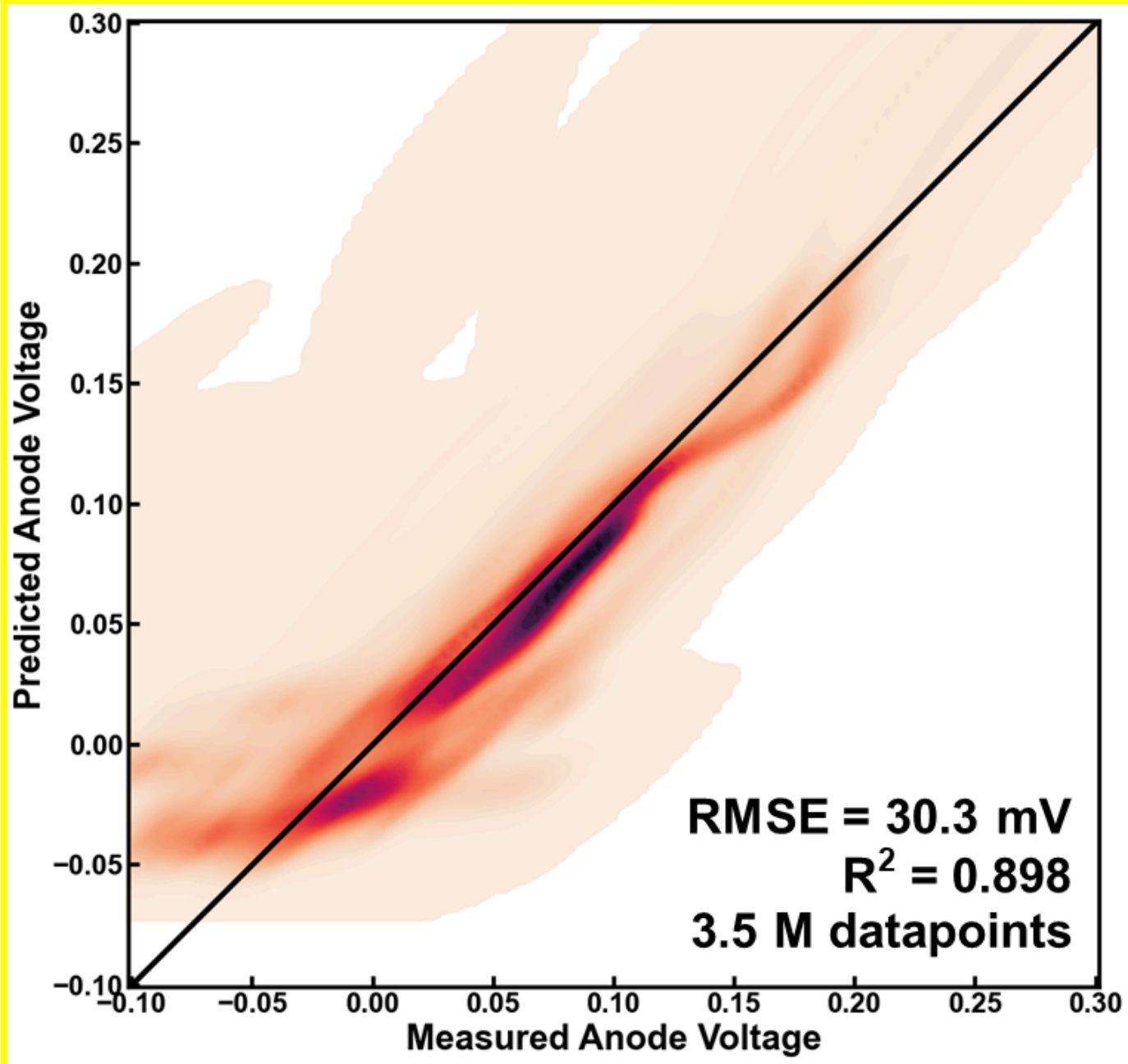

Figure S14. 2D density plot of predicted AV versus measured AV in all cells in test with full-voltage domain(2-3.6 V) and CV-stage included

Specific Comments R4-3. *You use AV<0 as the thermodynamic plating criterion, but real plating depends on kinetics, temperature, overpotential, and local current density. How does the method handle scenarios where AV is slightly above 0 yet localized plating occurs?*

Authors' Response:We fully agree that lithium plating is governed not only by thermodynamics, but also by kinetics, temperature, local overpotential, and current-density heterogeneity(*J. Power Sources.*, 2023, **573**, 233118). Localized plating may occur even when the average anode potential is slightly above 0 V versus Li/Li^+. In this work, we therefore use AV < 0 V as a conservative and physically interpretable indicator of conditions under which lithium plating becomes thermodynamically favorable, rather than as a strict binary criterion for microscale plating onset.

Under the experimental conditions studied here, i.e., coin cells operated at moderate C-rates and near-ambient temperature, the anode potential serves as a reliable global proxy for plating risk. Importantly, our SEM validation demonstrates a clear correlation between predicted anode voltage levels and the extent of lithium deposition, supporting the practical relevance of this

indicator for the present system. Even when AV approaches but does not substantially cross 0 V, the model captures relative changes in plating severity through continuous AV prediction rather than reliance on a hard threshold.

We emphasize that the proposed framework does not treat AV < 0 V as a strict classifier, but instead provides a continuous, real-time estimate of AV, which can be integrated with additional kinetic or spatial indicators in future implementations. Under more aggressive operating conditions, such as low temperature, high C-rate, or pronounced spatial current nonuniformity, localized kinetic plating above 0 V is expected to become more prominent. Incorporating such effects through extended validation, including spatially resolved measurements or temperature-dependent modeling, represents a natural direction for future work.

In this context, we note that our group is developing a high-precision local AV platform (**Figure R2**), in which lithium metal is electrodeposited on a reference electrode to probe localized electrochemical behavior. This capability provides a pathway to infer local AV and spatially heterogeneous plating conditions beyond the cell-averaged signal. Integrating such local diagnostics with the present machine-learning framework will offer a promising route toward a more comprehensive diagnosis of lithium plating that accounts for both global thermodynamic indicators and localized kinetic effects.

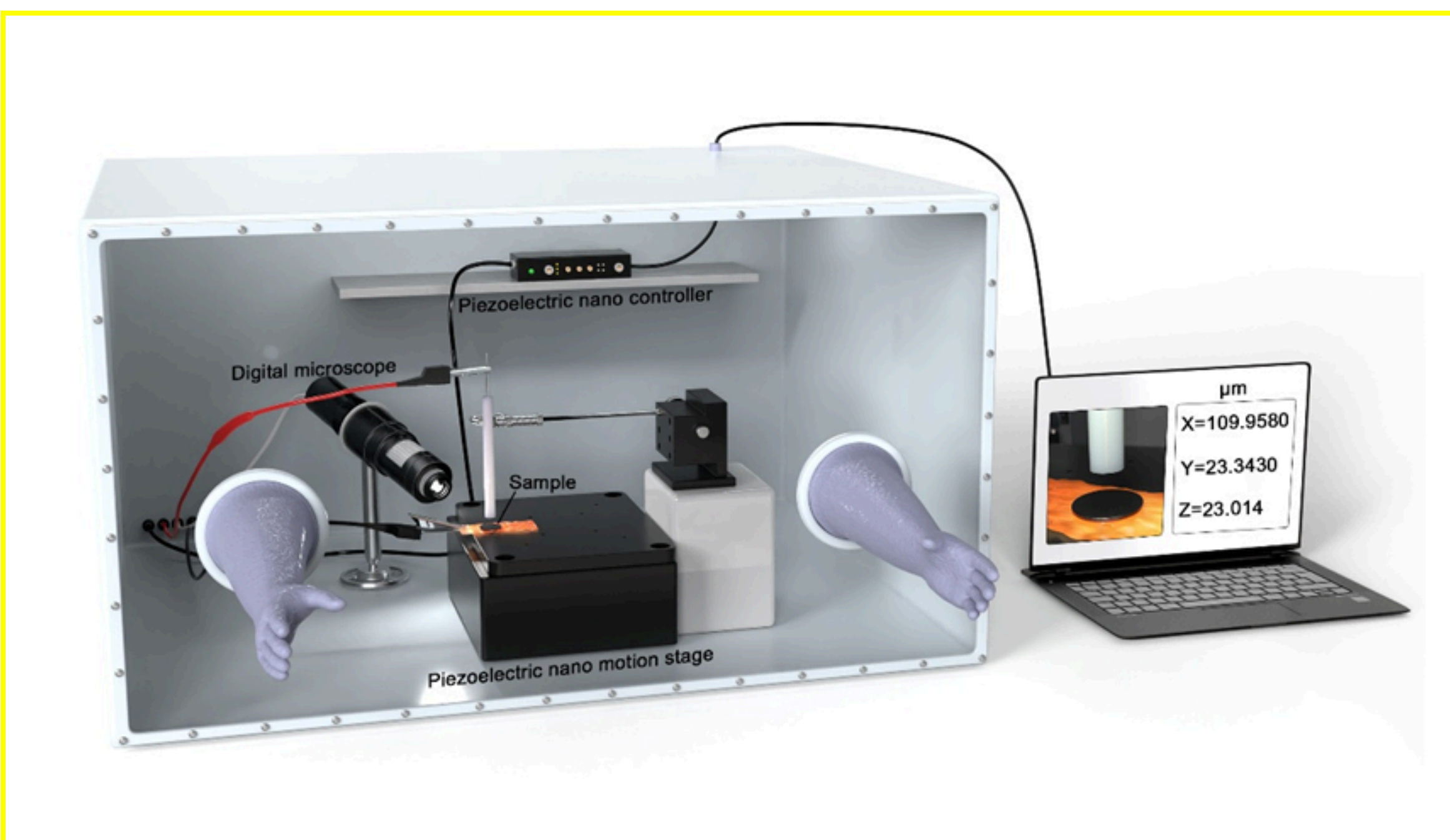

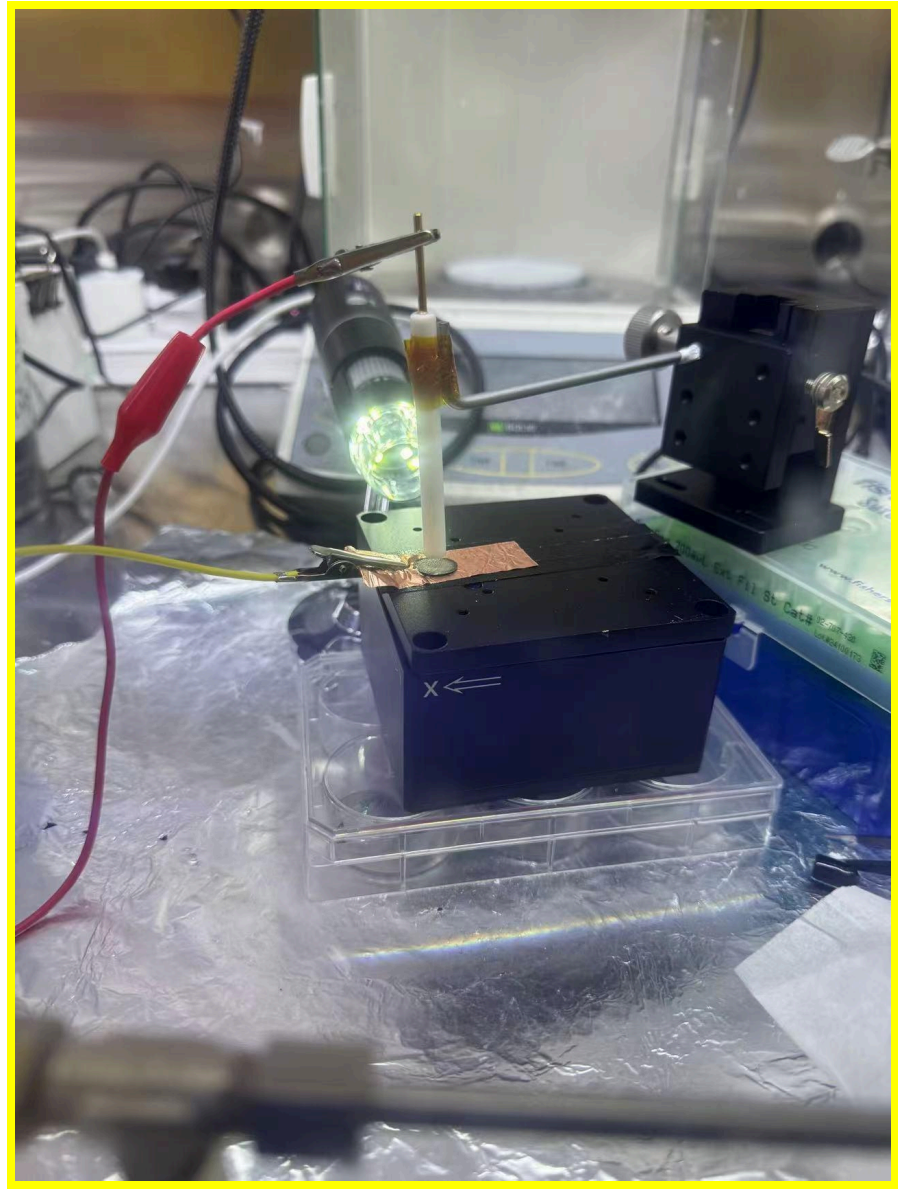

Figure R2. schematic and photos of the local AV measurement platform

Specific Comments R4-4. *SEM validation uses two points (predicted 0.01 V and -0.02 V). Is this anecdotal? Please provide more SOC points and replicated cells (e.g., n≥3), and include quantitative metrics (coverage, deposit thickness distribution), not only qualitative images.*

Authors' Response: We thank the reviewer for this constructive comment regarding the scope and quantitative rigor of the SEM validation.

In response, we have expanded the SEM validation to include four predicted anode voltage groups (0.01 V, 0 V, -0.01 V, and -0.02 V). For each AV condition, three independently tested cells were examined (n = 3), and SEM images and optical images are now provided in the revised **Figure 3** & **Supplementary Figure 15, and Supplementary Fig. 10, respectively**.

In addition to qualitative imaging, we now include quantitative analysis of lithium deposition, where the coverage of lithium plating (including nanoparticle nuclei and larger deposits, highlighted in red in **Supplementary Figure 15**) is quantified in two pathways. Firstly, it is calculated as the ratio of plating pixel area to the total image pixel area. Image processing and area quantification were performed using ImageJ, enabling a consistent and objective comparison across anode voltage conditions and replicate cells. Secondly, the grayscale analysis was performed. Each image was converted into 8-bit grayscale values (0–255), and the pixel density distribution for each gray level was obtained. Based on the specific brightness of each image, a threshold was selected to represent lithium (this step is necessary because the

brightness and contrast vary among SEM images). The lithium coverage was then quantified as the area fraction of thresholded pixels in each image. Relative results are included in **Supplementary Figure 16**.

These additions provide both replicated experimental validation and quantitative metrics, addressing the reviewer's concern regarding anecdotal evidence.

Manuscript edit:

Main text, **Page 12-13:**

In contrast, conventional 2E systems can only provide the overall cell voltage, which is unable to directly detect metallic Li plating processes **(Fig. 3a)**. Using our virtual reference electrode in our Gr||LFP 2E cell, we can continuously monitor the AV during charging (**Fig. 3b-e**) *without additional sensing equipment*, and perform post-mortem analysis of the graphite surface at various charging states (3 duplicate cells for each charging state). At a predicted AV just **above** (i.e., 0.01 V, shown in **Fig. 3b**) and **at** the Li plating threshold (i.e., 0 V, shown in **Fig. 3c**), our scanning electron microscopy (SEM) experiments & optical images show that the graphite surfaces are smooth and similar to pristine graphite, indicating that Li metal plating has not occurred (**Fig. 3f&g** and Supplementary Fig. 15 a,e,i & b,f,j). However, when predicted AV is just **below** the Li plating threshold (i.e., -0.01 V, shown in **Fig. 3d**), shiny Li particles are captured on graphite surfaces via SEM (**Fig. 3h** and Supplementary Fig. 15 c,g,k), indicating the ongoing Li nucleation stage. At later stages of charging where our virtual reference predicted a more negative AV value (-0.02 V, shown in **Fig. 3e**), we clearly observed filamentary and mossy structures on the graphite surfaces via SEM, which are direct markers of Li metal plating (**Fig. 3i** and Supplementary Fig. 15 d,h,l), even if the plating is not visible in the optical image (Supplementary Fig. 10, which demonstrates the acuity of our smart sensing approach). Furthermore, we quantitatively analyze the Li plating coverage across four predicted AV states

(See methods and Supplementary Fig. 16). Li coverage is nearly zero when the predicted AV is 0.01 or 0 V, whereas it increases to >10% at a predicted AV of -0.01 V and further to >50% at -0.02 V, further validating the robustness of our ML-based diagnosis approach.

Supplementary information, **Page 15:**

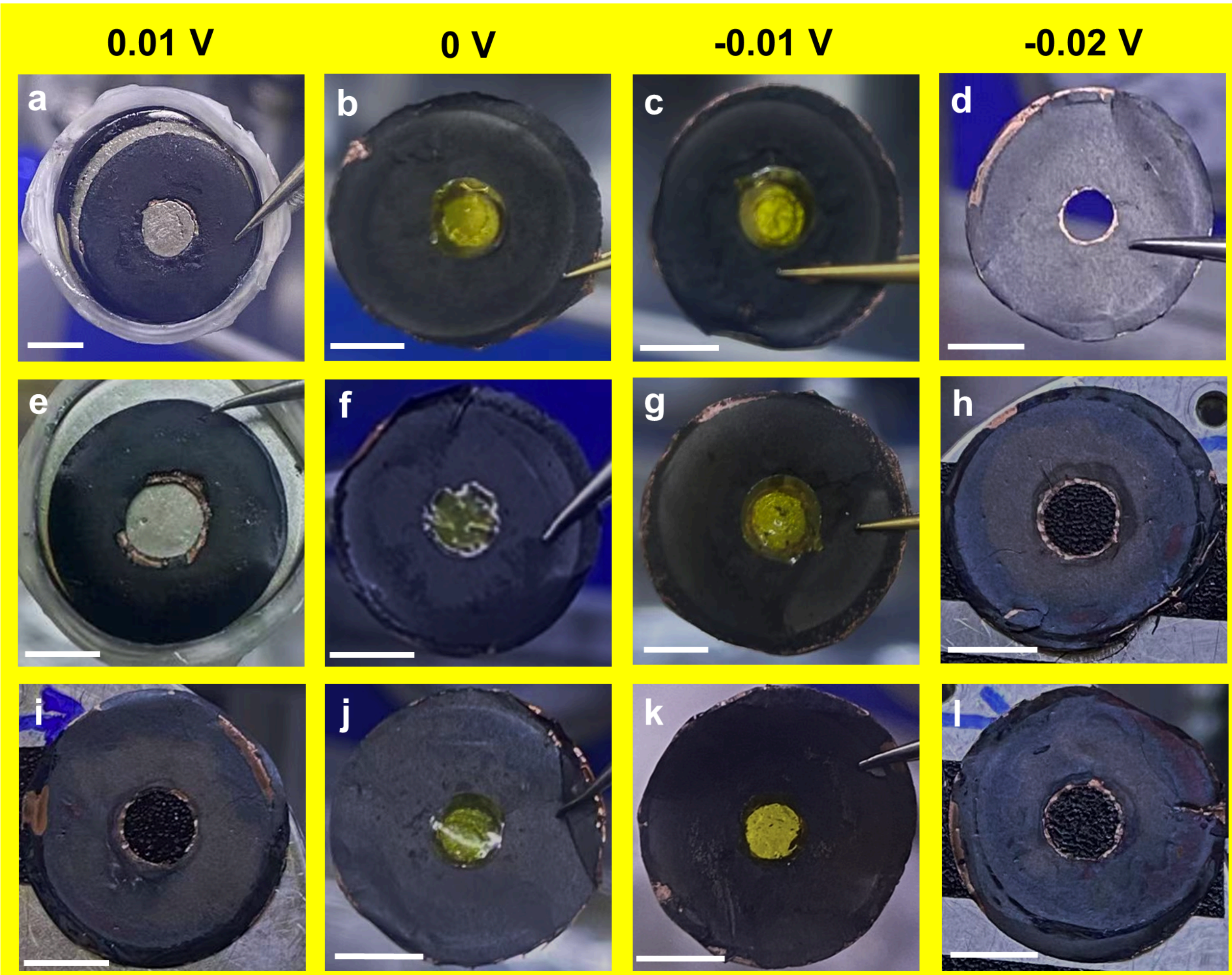

Figure S10. Optical image of graphite electrode for predicted AV of (a,e,i) 0.01 V; **(b,f,j)** 0 V; **(c,g,k)** -0.01 V; **(d,h,l)** -0.02 V. Scale bar: 5 mm

Supplementary information, Page 19:

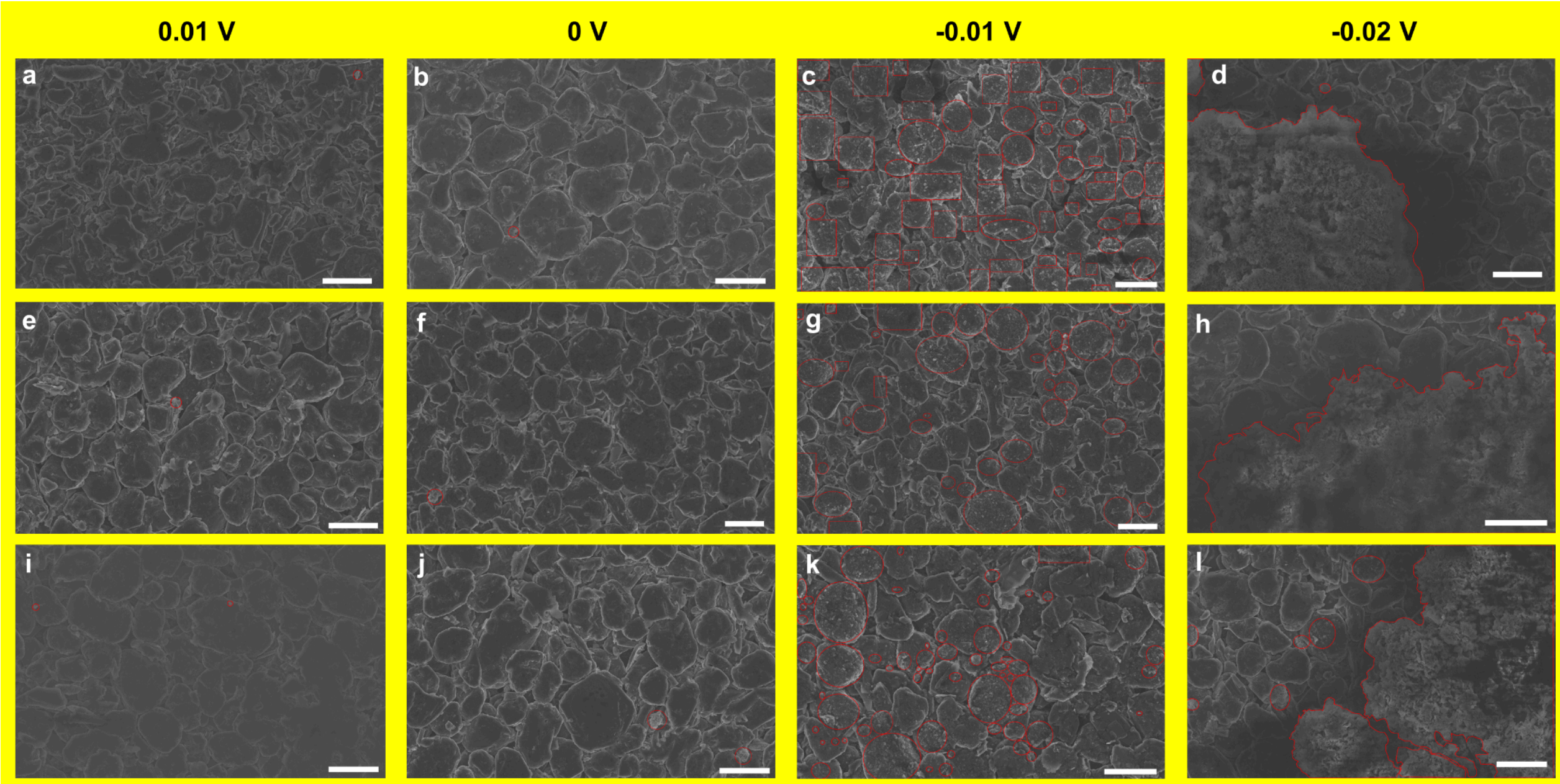

Figure S15. SEM images of graphite electrodes for predicted anode voltage (AV) of (a,e,i) 0.01 V; **(b,f,j)** 0 V; **(c,g,k)** -0.01 V; **(d,h,l)** -0.02 V. Figure 3f-i correspond one-to-one with Figure S15 a-d, respectively, and present cropped regions from the same SEM images for improved layout. All regions outlined in red indicate areas identified as lithium plating, including both Li particle nuclei and larger deposited lithium features. Scale bar: 20 µm

Supplementary information, Page 20:

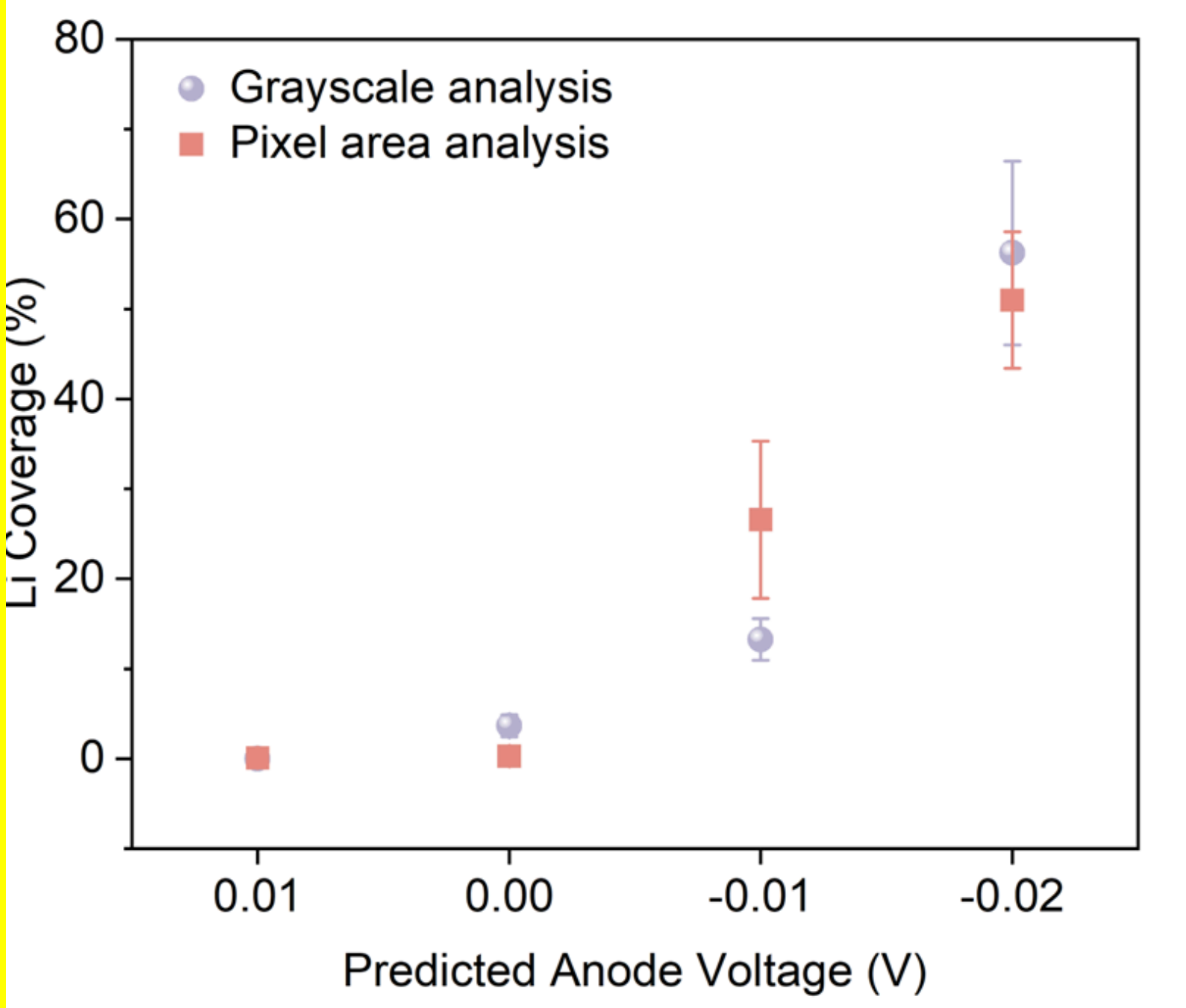

Figure S16. Li coverage with error bars for different predicted anode voltages. Li coverage was quantified from the SEM images in Fig. S15 via grayscale and pixel area analysis, by summing the pixel areas of all Li plating regions (including nuclei and large plating features, highlighted in red in Fig. S15) and normalizing by the total image pixel area. Data are presented as mean ± standard deviation across three cells per condition.

Supplementary information, Page 9:

Li coverage(%) estimation from SEM images

Lithium coverage was quantified using two independent quantitative analysis approaches on ImageJ: pixel area analysis and grayscale analysis. For the pixel area analysis, lithium coverage was calculated by summing the pixel areas of all lithium-plating-related regions, including both nucleation sites and larger plating features, as identified in SEM images (highlighted in red in Fig. S15). Then the total lithium-plated pixel area was then normalized by the total pixel area of

the corresponding SEM image to obtain the lithium coverage fraction. For grayscale analysis, each SEM image was first converted to an 8-bit grayscale format (intensity range: 0-255), and the pixel density distribution across grayscale levels was obtained. Owing to variations in brightness and contrast among SEM images, an image-specific grayscale threshold was selected to represent lithium-plated regions. Lithium coverage was then quantified as the area fraction of pixels exceeding this threshold relative to the total image area. For both methods, data are reported as the mean ± standard deviation across three independent cells.

Specific Comments R4-5. *You emphasize N/P≈1.0 as a stress test, but many commercial cells have higher N/P. Please discuss whether AV distributions and prediction error change for typical N/P (1.1–1.2), and whether smart-charging benefits remain significant*

Authors' Response: We thank the reviewer for this insightful comment on N/P ratio.

While an N/P≈1.0 configuration represents a stringent stress test for lithium plating susceptibility, we note that increasing the N/P ratio primarily results in a vertical shift of the anode voltage (AV) profile toward more positive values under identical operating conditions (**Fig. R3**). Importantly, for typical commercial N/P ratios, the anode voltage can still transiently enter negative regimes under fast-charging or low-temperature conditions, where lithium plating becomes thermodynamically favorable(*Nat. Energy.*, 2019, **4**, 540-550; *J. Power Sources.*, 2025, **660**, 238565). As a result, the overall AV distribution shape and its temporal evolution remain similar, and the predictive task addressed by our model is preserved. Consequently, the smart-charging benefits enabled by accurate AV prediction, i.e. early detection and mitigation of negative AV excursions, are expected to remain significant at higher N/P ratios, although the absolute AV baseline is shifted. **In future work, we will systematically collect AV data across different N/P ratios and leverage a transfer learning algorithm to quantify the sensitivity of AV prediction to electrode balancing, with the goal of training a more universal model capable of predicting AV across a broad range of N/P ratios, including those relevant to commercial lithium-ion batteries.**

More broadly, the ability to enable safe fast charging at lower N/P ratios in this work, where energy density is maximized but plating risk is most severe, highlights the potential of our

data-driven approach to inform the design and operation of next-generation lithium ion batteries with higher energy density. We anticipate that extending real-time AV-based diagnostics and control to such aggressive electrode-balancing regimes will provide valuable guidance for future research on safe lithium ion batteries at reduced N/P ratios.

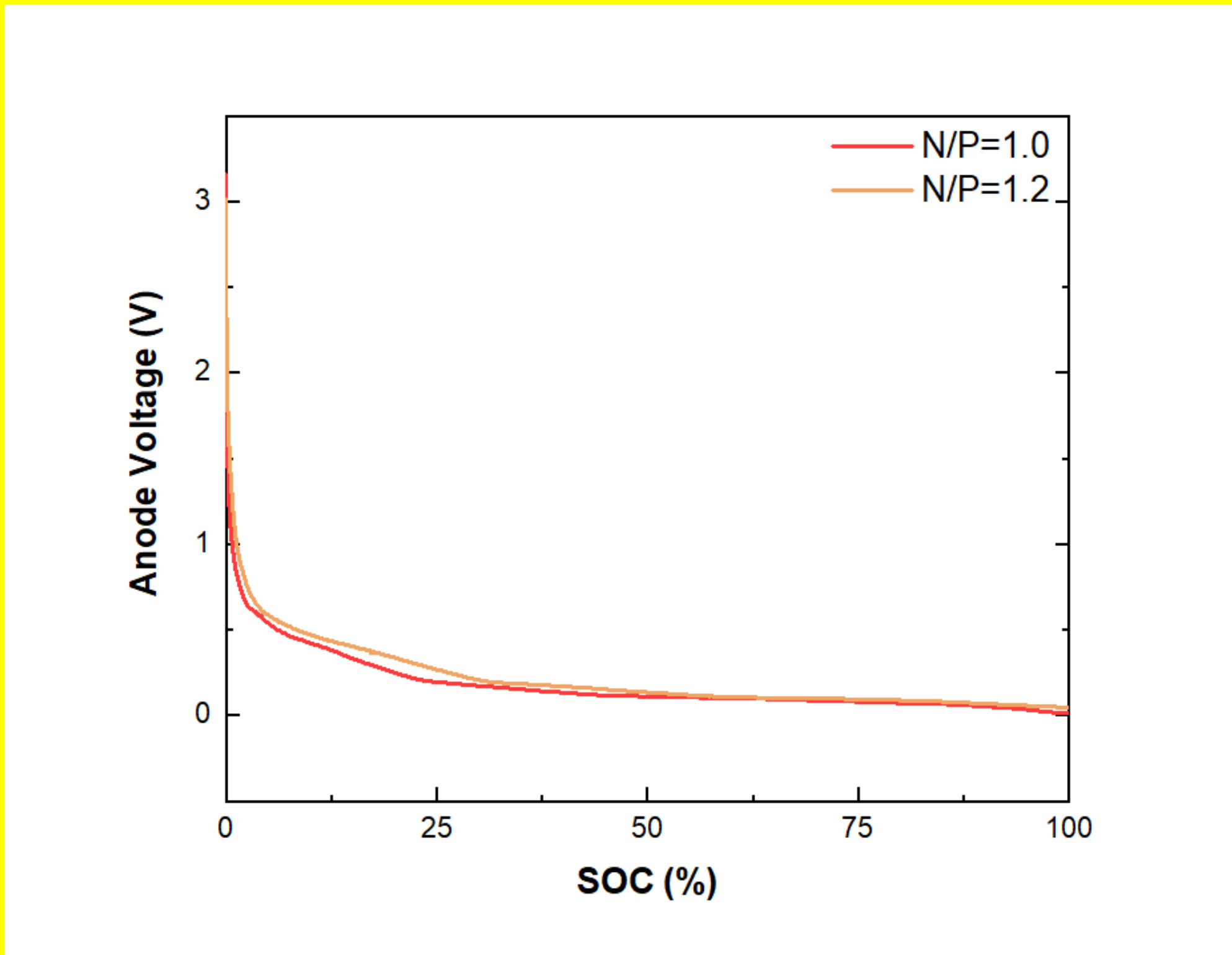

Figure R3. Anode Voltage vs state of charge(SOC) during C/10 charging process for N/P=1.0 and N/P=1.2 cells

Specific Comments R4-6. *Each ensemble network uses two 256-unit layers, 70 epochs, and 20% dropout. Please provide learning curves, early stopping/regularization details, variance across random seeds, and justify why such a large network is necessary with only 5 input features.*

Authors' Response: We thank the reviewer for this insightful comment on learning curves. We provide learning curves for a single ensemble of 5 models in **Supplementary Figure 17**, and learning curves for 12 models each with a different random seed in **Supplementary Figure 18**. Overall, the learning process is quite stable across seeds and models reliably converge to a performance plateau. We did not employ any early stopping, but used L1 regularization on the model parameters with a weight of 1e-4.

For network size, despite there being only 5 input features, we found 256 hidden units were necessary due to the complexity of the function we wish to learn. Prior work has shown that the learning capability of a network is closely related to its width, and so despite only 5 input features, we find the functions we wish to learn require 256 width layers (*Neural Networks*, 1989, **2**, 359-366; Goodfellow et al., *Deep Learning*, MIT Press, 2016).

Manuscript edit:

Supplementary information, **Page 6:**

Early stopping is not employed, but L1 regularization on the model parameters with a weight of 1e-4 is used. Relative learning curves and variance across random seeds are provided in **Supplementary Fig.17 & 18**, respectively.

Supplementary information, **Page 21:**

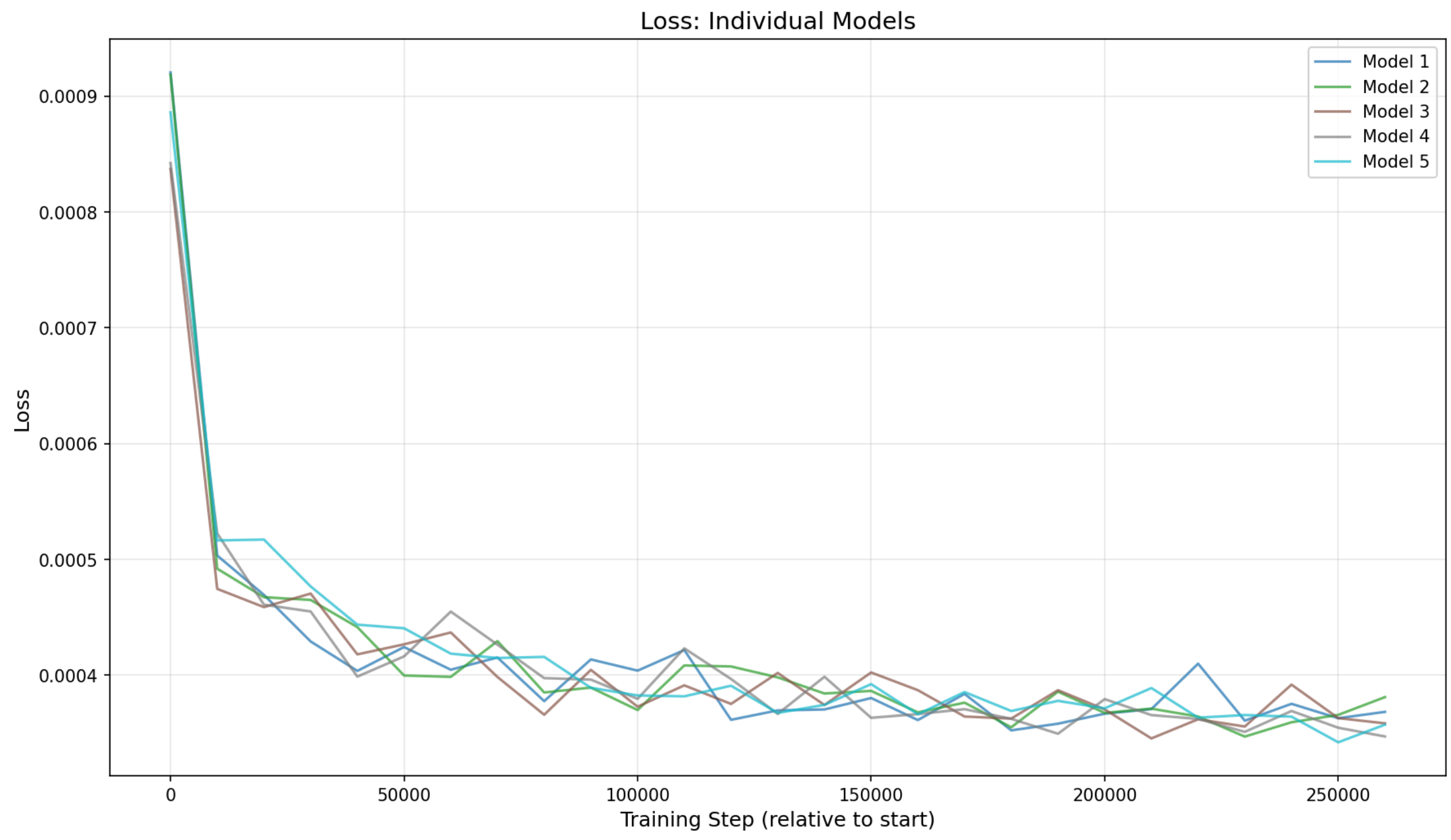

Figure S17. Test loss over the course of training

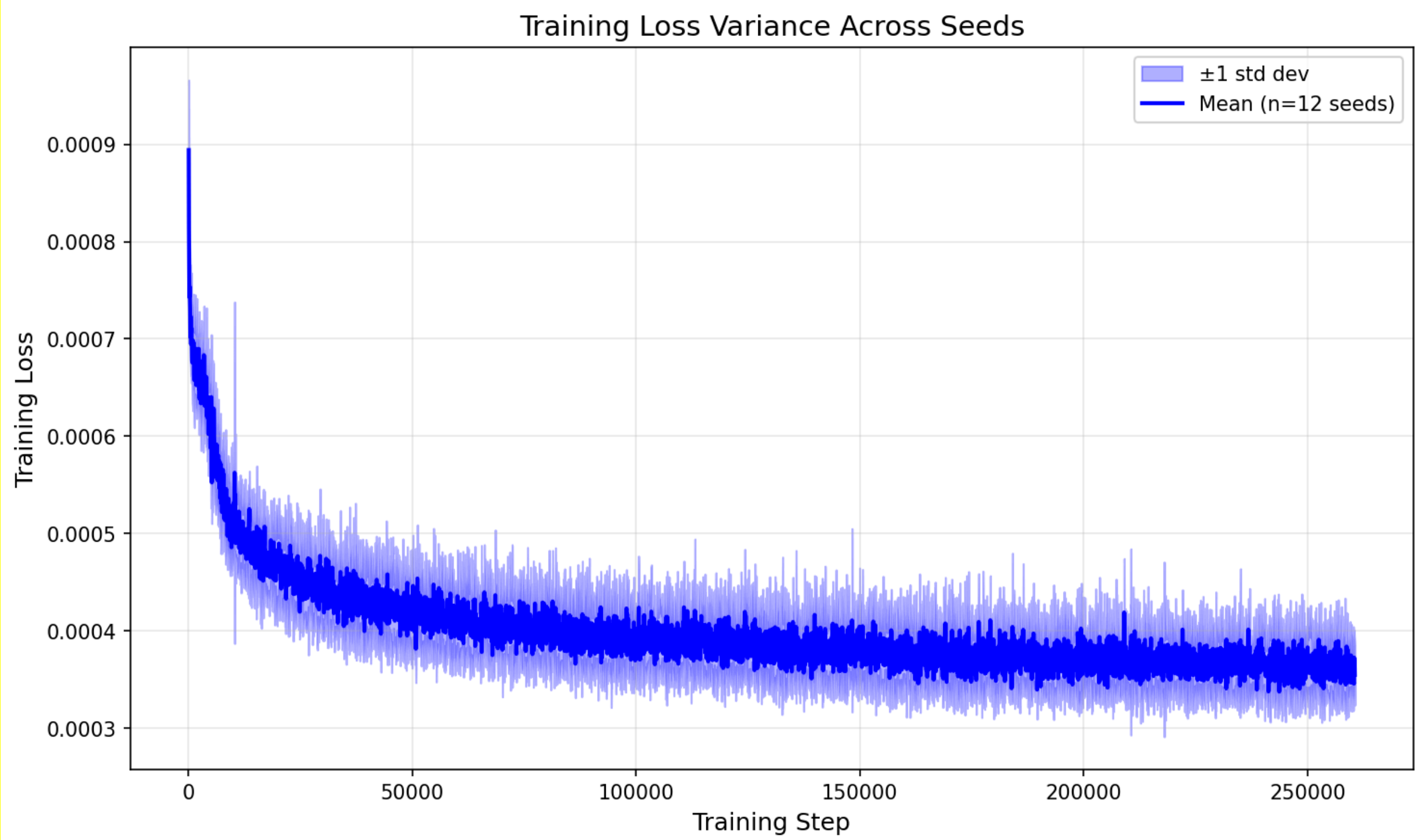

Figure S18. Mean and Std Dev of training loss across 12 different seeds. The dark line shows the mean while light blue shows ± 1 std dev

Specific Comments R4-7. *The response and SI says parameters are tuned using measured voltage-current over many cycles. Please clarify whether the PBM is re-tuned per cycle or tuned early and extrapolated.*

Authors' Response: We thank the reviewer for requesting clarification regarding the parameter-tuning procedure of the physics-based model (PBM).

The PBM parameters are calibrated using current-voltage measurements from an early reference cycle for each battery cell. After this initial calibration, the parameters are held fixed for subsequent cycle predictions, and no cycle-by-cycle tuning is performed. Later-cycle behavior is therefore predicted in a forward manner without additional parameter adjustment.

Because intrinsic properties and initial conditions vary slightly from one battery cell to another, so a one-time calibration is performed individually for each cell to account for cell-to-cell variability.

However, within a given cell, the calibrated parameters remain unchanged across cycles.

We have clarified this point in the Supplementary Information to explicitly state that the PBM is not re-tuned per cycle, but instead calibrated once per cell and then used for extrapolative prediction across aging.

Manuscript edit:

Supplementary information, **Page 8:**

For each cell, the PBM parameters were calibrated once using current-voltage data from an early reference cycle and were subsequently held fixed for all later-cycle predictions, without any cycle-by-cycle re-tuning.

Specific Comments R4-8. *Smart charging uses a C# interface to the cycler and calls Python models. Please report the end-to-end closed-loop latency (sampling→prediction→current update) and describe fail-safe strategies under jitter, packet loss, or outliers.*

Authors' Response: We thank the reviewer for requesting clarification on the closed-loop latency of the smart-charging implementation.

The Arbin CTI control framework is designed to support parallel control of a large number of channels across multiple microcontroller units (MCUs). According to system-level measurements, the average latency for sending a control command through CTI is within 20 ms. Consequently, the round-trip communication latency, including command dispatch and acknowledgment, is approximately 40 ms.

For model inference, each neural network in the ensemble requires approximately 15 ms, resulting in a total inference latency of ~75 ms for the five ensembles. Taken together, the overall closed-loop latency (sampling → communication → prediction → current update) is approximately 115 ms, with minor variations depending on the host PC configuration.

During dynamic charging, data are sampled at a temporal resolution of 2 s per datapoint. Therefore, the closed-loop latency is more than an order of magnitude smaller than the control update interval and has a negligible impact on control performance or stability under the operating conditions studied.

Manuscript edit:

Supplementary information, **Page 7-8:**

The end-to-end closed-loop latency of the smart-charging system, including data sampling, communication via the Arbin CTI interface, ensemble model inference, and current update, is approximately 115 ms under the experimental configuration used.

Response Letter to Reviewers' Comments

Manuscript ID: NCOMMS-25-27724D

Title: Battery smart sensing via a virtual reference electrode

Authors: Jiayi Yu, Aki Takahashi, Min-Ho Kim, Rishi Upadhyay, Howard Zhang, Tian-Yu Wang, Huayang Zhu, Robert Kee, Tyrone Vincent, Bo Liu, Xintong Yuan, Thomas Hymel, Kaiyan Liang, Keyue Liang, Haoyang Wu, Dingyi Zhao, Jung Tae Kim, Achuta Kadambi✉, Yuzhang Li✉

Response to Reviewer #4

Specific Comments R4-1. *The rebuttal usefully shows that when the model is trained only on constant-current charging data and tested on stepped-charging protocols, the test error increases from approximately 23 mV to approximately 38 mV. While this supports some degree of extrapolative capability, I would still encourage the authors to more systematically define the generalization boundary of the model with respect to unseen charging protocols. Beyond stepped charging, has the model been evaluated under more complex multi-stage protocols, different cutoff voltages, different rest durations, or different temperature conditions? Providing error statistics stratified by protocol type, rather than only an aggregate RMSE, would help clarify the model's practical applicability under realistic BMS operating scenarios.*

Authors' Response:

We thank the reviewer for this thoughtful suggestion. We agree that clearly defining the model's generalization boundary with respect to unseen charging protocols is important for assessing its practical applicability.

In this work, we performed an explicit extrapolation test in which the model was trained only on constant-current charging data and evaluated on unseen stepped-charging protocols. Under this setting, the RMSE increased from ~23 mV to ~38 mV (**Figure R1**), indicating that the model retains meaningful predictive capability under protocol shift, while also showing that accuracy degrades when the charging profile departs from the training distribution. We have revised the text to make this generalization boundary clearer.

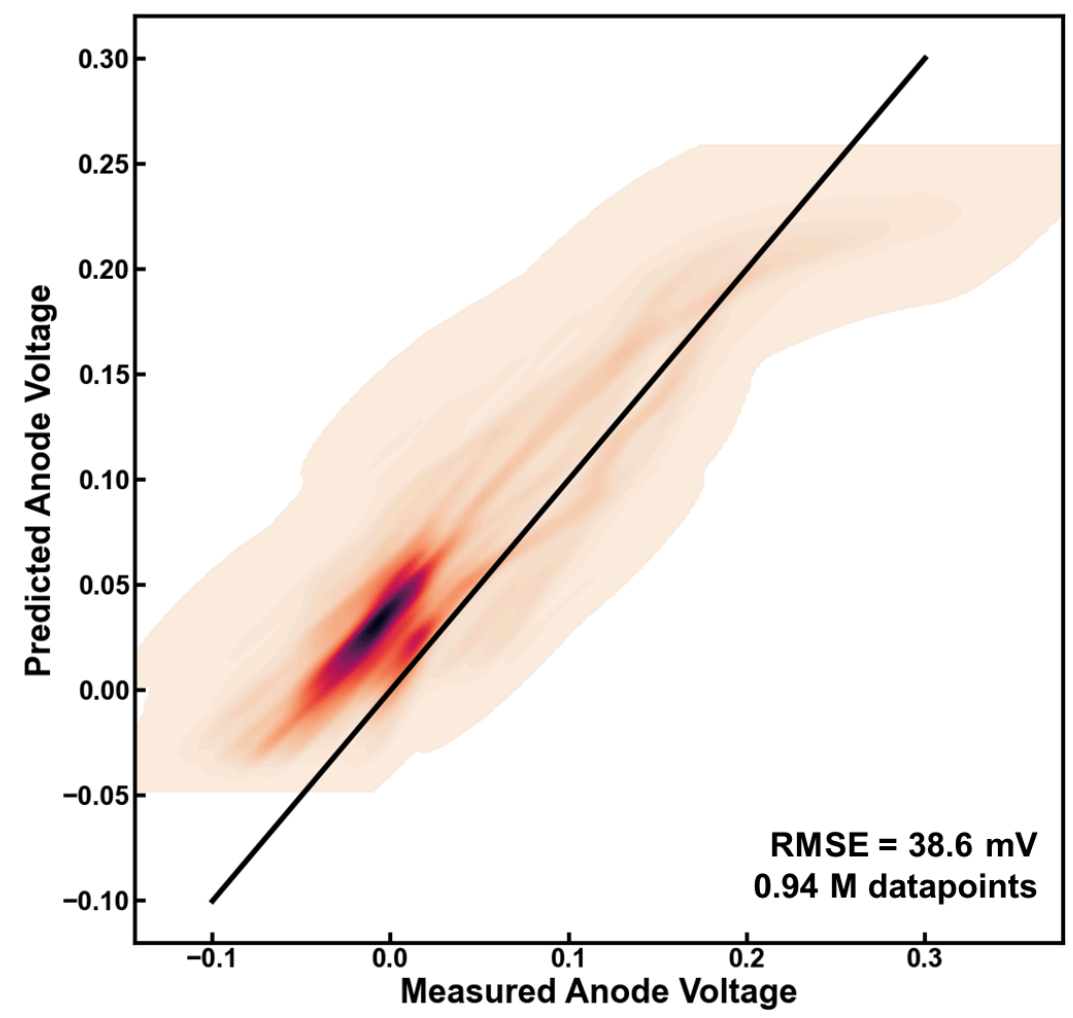

Figure R1. Model performance while training on CC group but test on stepped group

In addition, we evaluated the model in closed-loop smart-charging experiments over the full cycling process, including both early and late-life cycles, where the charging current dynamically changed in response to the predicted anode voltage, and these dynamic protocols remain unseen by the ML models during the training process. These results further support that the model remains practically useful under non-constant and time-varying current profiles beyond the fixed constant-current protocols used for model training.

We also note that, in response to Reviewer 4's earlier comment regarding domain coverage, we trained and evaluated an additional model over a wider voltage range that includes the lower-voltage region and the CV stage, which yielded an RMSE of ~30.3 mV with R^2=0.898. This result further suggests that the framework is reasonably robust when the operating domain is expanded beyond the primary high-risk charging region emphasized in the main text.

Here we summarized the performance of the ML model under 1) Train on CC cells, test on stepped cells; 2) unseen charging protocols and 3) various voltage domains in **Table S8**.

Validation condition	RMSE(V)
Unseen 7-step smart charging*-1st cycle	0.0205
Unseen 7-step smart charging*-300 th cycle	0.0227
Train on CC cells only, test on stepped cells only	0.0382
3-3.6 V cutoff voltage domain, w/ CV stage	0.0303
**3.3-3.6 V cutoff voltage domain, w/o CV stage**	**0.0226**
3.3-3.4 V cutoff voltage domain, w/o CV stage	0.0209
3.4-3.5 V cutoff voltage domain, w/o CV stage	0.0194
3.5-3.6 V cutoff voltage domain, w/o CV stage	0.0288

Table S8. Generalization performance of the ML model under unseen charging protocols and different voltage domains.

***In this 7-step smart-charging protocol, charging starts at 1C and is reduced in 0.1C steps when the predicted AV falls below 0.02 V until 0.4C. This protocol differs from the stepped protocol used in the training set, where a three-step 1C-0.7C-0.5C protocol was applied. The AV thresholds used in the training-set stepped protocol were: -0.15 V for cells #48–49, -0.05 V for cell #50, -0.04 V for cell #51, and -0.01 V for cells #52–55.**

We agree with the reviewer that a more systematic stratification of model performance across a broader range of protocol variations, such as different rest durations and temperature conditions, would be highly valuable. We are currently planning follow-up studies along these directions, including experiments enabled by the acquisition of a larger temperature-control system, which will allow more systematic evaluation under broader and more practically relevant operating conditions. We view this as an important next step for further extending the applicability of the framework beyond the scope of the present study.

To clarify this point for readers, we have added text in the revised manuscript noting that the present study demonstrates generalization to unseen stepped and dynamically controlled charging profiles, but that a full mapping of model performance across broader BMS-relevant operating conditions remains a subject for future investigation.

Manuscript edit:

Main text, Page 17:

With our preliminary smart BMS charging protocol, we have shown a substantial enhancement in the cycle retention of low-N/P-ratio cells without any changes in cell chemistry or architecture. Although the present study demonstrates generalization to unseen stepped, dynamically controlled charging profiles, and different cutoff voltage domains (Table S8), a comprehensive mapping of model performance across broader BMS-relevant operating conditions, including temperature, and rest duration, remains an important direction for future investigation. Future work may also integrate advanced process-control optimization with our virtual reference electrode to further improve battery performance.

Supplementary information, Page 31:

Table S8. Generalization performance of the ML model under unseen charging protocols and different voltage domains.

**Validation condition**	**RMSE(V)**
Unseen 7-step smart charging*-1st cycle	0.0205
Unseen 7-step smart charging*-300 th cycle	0.0227
Train on CC cells only, test on stepped cells only	0.0382
3-3.6 V cutoff voltage domain, w/ CV stage	0.0303
**3.3-3.6 V cutoff voltage domain, w/o CV stage**	**0.0226**
3.3-3.4 V cutoff voltage domain, w/o CV stage	0.0209
3.4-3.5 V cutoff voltage domain, w/o CV stage	0.0194
3.5-3.6 V cutoff voltage domain, w/o CV stage	0.0288

***In this 7-step smart-charging protocol, charging starts at 1C and is reduced in 0.1C steps when the predicted AV falls below 0.02 V until 0.4C. This protocol differs from the stepped protocol used in the training set, where a three-step 1C-0.7C-0.5C protocol was applied. The AV thresholds used in the training-set stepped protocol were: -0.15 V for cells #48–49, -0.05 V for cell #50, -0.04 V for cell #51, and -0.01 V for cells #52–55.**

Specific Comments R4-2. *The authors have clarified that the train/test partition is performed strictly at the cell level, which avoids leakage across cycles from the same cell; meanwhile, the PBM comparison relies on a one-time early-cycle calibration for each cell, with fixed parameters thereafter. In light of this, I encourage the authors to state more explicitly whether the ML model requires absolutely no per-cell calibration when deployed on a new cell. In addition, the current validation still appears to remain within one chemistry and one experimental distribution. If the model is transferred to cells from different manufacturing batches, electrolyte formulations, electrode loadings, or broader cell-to-cell variability, can it still be used directly? An external validation across batches or manufacturing variability would substantially strengthen the claim of general applicability.*

Authors' Response:

We thank the Reviewer for this important comment. We have revised the manuscript to clarify explicitly that, once the ML model is trained, it is deployed on new cells without any per-cell calibration, retraining, or fine-tuning. In the real-time validation and smart-charging demonstrations in this work, the model was directly applied to unseen test cells using the same shared learned weights to enable a fair comparison with the PBM workflow. To further examine whether cell-to-cell and batch-to-batch differences affected model performance, we quantified the prediction error across 8 cells in test set deliberately selected from different experimental batches, using the same trained ML model without any per-cell calibration, retraining, or fine-tuning (**Table S9**). This analysis therefore evaluates whether the shared ML model can maintain consistent prediction accuracy across independently prepared cells, rather than only across cells from a single batch.

	**Mean**	**Std Dev**
**RMSE(V)**	0.0263	0.0088
**MAE(V)**	0.0217	0.0085
**R^2**	0.821	0.071

Table S9. Cell-to-cell variability analysis of ML prediction performance across cells in test set

As summarized in Table S9, the model achieved a mean RMSE of 0.0263 V and a mean MAE of 0.0217 V, with standard deviations of 0.0088 V and 0.0085 V, respectively. The corresponding R2 value was 0.821±0.071. These results indicate that, although moderate cell-to-cell variability

is present, the prediction accuracy remains within a similar error range across the cells in the test set.

We also agree with the reviewer that the present core validation is conducted within the chemistry and experimental distribution represented by the current dataset. At the same time, because the train/test split is performed strictly at the cell level across a dataset containing 55 cells, the reported test performance already reflects robustness to the intrinsic cell-to-cell and manufacturing-related variability present within these cells.

To further address the reviewer’s concern regarding broader applicability, we note that we have obtained preliminary transfer learning results across additional chemistries and configurations, including **different chemistry** (i.e., another LFP material from different manufacturer(Argonne CAMP facility), and NCM811), **different electrolyte** (1 M $LiPF_6$ in EC/DEC (1:1) with 10% VC), and **different cell architectures**, i.e. LFP pouch cells. These preliminary results, summarized in **Figure. R2 and Table R1**, suggest that the framework is adaptable beyond the baseline training distribution. However, we agree that these results are still preliminary. Therefore, we present them here as supporting evidence of extensibility, while reserving a more comprehensive analysis for future follow-up work.

[Figure Redacted]

[Table Redacted]

Manuscript edit:

Main text, Page 9:

To further assess whether cell-to-cell batch variability affects model robustness, we evaluated prediction performance consistency across 8 cells in the test set deliberately selected from different experimental batches using the same trained ML model without per-cell calibration, retraining, or fine-tuning (Table S9). The results indicate that the model maintains comparable prediction accuracy across unseen cells, supporting its applicability to new cells across different batches.

Supplementary information, Page 6:

Early stopping is not employed, but L1 regularization on the model parameters with a weight of 1e-4 is used. Relative learning curves and variance across random seeds are provided in **Supplementary Fig.17 & 18**, respectively. To fairly compare ML results with physics-based model(PBM), once the ML model is trained, it is deployed on new cells without any per-cell calibration, retraining, or fine-tuning.

Supplementary information, Page 31:

Table S9. Cell-to-cell variability analysis of ML prediction performance across cells in test set

	**Mean**	**Std Dev**
**RMSE(V)**	0.0263	0.0088
**MAE(V)**	0.0217	0.0085
**R^2**	0.821	0.071

Specific Comments R4-3. *The rebuttal appropriately acknowledges that localized lithium plating may already occur when the average AV remains slightly above 0 V, and therefore AV < 0 V should be interpreted as a physically meaningful thermodynamic risk indicator rather than a strict binary classifier. Since the proposed smart-charging strategy is built around this indicator, I would encourage the authors to further clarify how the framework avoids false negatives in the near-threshold regime where AV is still slightly positive but localized plating may already emerge. At present, the 0.02 V safety buffer appears to be chosen primarily based on the reported MAE of about 0.018 V. A more direct risk-oriented analysis, such as a threshold sensitivity study or a false-positive/false-negative tradeoff near the plating threshold, would make the control rationale more convincing.*

Authors' Response: We thank the Reviewer for this insightful comment. We agree that although 0 V represents the thermodynamic onset for Li plating, localized plating may already emerge when the spatially averaged anode voltage (AV) remains slightly positive because of electrode heterogeneity and kinetic effects. Accordingly, in our framework, AV < 0 V is not treated as a strict binary classifier, but rather as a physically meaningful thermodynamic risk indicator. Consistent with this interpretation, the smart-charging controller is intentionally designed to operate conservatively in the near-threshold regime.

Specifically, the choice of the 0.02 V control threshold was not based on model error alone, but on multiple complementary considerations. First, the model achieves an MAE of approximately 0.018 V, so a positive safety margin of similar magnitude provides a conservative buffer against prediction uncertainty. Second, we performed a direct false-negative analysis on the full 2.2 million-point test set by quantifying the fraction of cases in which the true AV was already negative while the predicted AV remained above candidate control thresholds (**Table S5**). The false-negative rate decreased from 2.140% at a 0 V threshold, to 1.445% at 0.01 V, and to 0.947% at 0.02 V. Thus, increasing the control threshold above 0 V systematically reduces the probability of missing plating-risk points in the near-threshold regime. In our control design, we therefore selected 0.02 V as a conservative engineering threshold because it was the first threshold that reduced the false-negative rate to below approximately 1%.

False-negative condition	False-negative rate(%)
Real AV < 0 V && Predicted AV ≥ 0.02 V	0.947
Real AV < 0 V && Predicted AV ≥ 0.01 V	1.445
Real AV < 0 V && Predicted AV ≥ 0 V	2.140

Table S5. False negative analysis for smart charging threshold settings

In addition, it was validated in previous research that early plating nuclei or residual plated Li may facilitate subsequent Li deposition during later cycling by lowering the energy barrier (*Adv. Energy Mater*. 2024, **14**, 2400894), making a conservative threshold particularly desirable even when only a very small amount of plating is initially present. As shown by the SEM-based Li quantification in Fig. S16, the Li coverage at a predicted AV of 0.01 V is already very close to zero but remains slightly positive, whereas substantially larger coverage is observed once AV becomes negative. This near-threshold behavior suggests that setting the control threshold above 0 V is appropriate when the goal is not merely to delay severe plating, but to more conservatively target Li-plating-free charging.

To further strengthen this rationale, we performed a post hoc SEM analysis of the graphite electrode cycled under the 0.02 V smart-charging protocol shown in **Fig. 4e&f** and **Fig. S11a&b**. Both grayscale and pixel-area quantification showed 0% Li coverage under this protocol after end-of-life, supporting that the selected threshold successfully avoids detectable plating in practice. We have revised the manuscript to clarify that the 0.02 V threshold is an engineering safety margin chosen to reduce false negatives in the near-threshold regime and to target plating-free operation, rather than a claim that plating onset occurs exactly when the average AV crosses 0 V.

Manuscript edit:

Main text, Page 16:

We set the safety threshold at 0.02 V as a conservative engineering margin, informed by model uncertainty and near-threshold false-negative analysis, to reduce missed plating-risk events and more conservatively target plating-free charging (details are provided in Supplementary Note #3).

Supplementary information, Page 29:

Table S5. False-negative analysis for smart-charging threshold settings

False-negative condition	False-negative rate(%)
Real AV < 0 V && Predicted AV ≥ 0.02 V	0.947
Real AV < 0 V && Predicted AV ≥ 0.01 V	1.445
Real AV < 0 V && Predicted AV ≥ 0 V	2.140

Supplementary information, Page 35:

Note #3. Rationale of 0.02 V threshold in smart charging

In this study, the smart-charging controller uses a predicted anode voltage (AV) threshold of 0.02 V as the control limit for current regulation. This value was selected as a conservative engineering safety margin, rather than to imply that Li plating begins exactly when the spatially averaged AV crosses 0 V.

Electrochemically, 0 V corresponds to the thermodynamic onset condition for Li plating. However, localized plating may already occur when the spatially averaged AV remains slightly positive because of electrode heterogeneity, local overpotentials, and kinetic effects[17,18]. Accordingly, in the present framework, AV < 0 V is interpreted as a physically meaningful plating-risk indicator rather than a strict binary classifier. Since the control objective is to more conservatively target Li-plating-free charging, the use of a threshold above 0 V is appropriate.

The selection of 0.02 V was based on two complementary considerations. First, the virtual reference electrode achieves a mean absolute error (MAE) of approximately 0.018 V on the test set, such that a positive margin of similar magnitude provides a practical buffer against prediction uncertainty near the plating threshold. Second, we performed a false-negative analysis

on the full 2.2 million-point test set near the threshold **(Table S5)**, defined as the fraction of cases where the true AV was already negative while the predicted AV remained above a candidate control threshold. The false-negative rates were 2.140% for a threshold of 0 V, 1.445% for 0.01 V, and 0.947% for 0.02 V. These results show that shifting the control threshold slightly above 0 V systematically reduces the probability of missing plating-risk points near the threshold. On this basis, 0.02 V was selected as a conservative threshold because it was the first tested value that reduced the false-negative rate to below approximately 1%.

This conservative choice is further supported by prior literature showing that early plating nuclei or residual plated Li can facilitate subsequent Li deposition during later cycling, making suppression of even very small initial plating events desirable[19]. Consistent with this interpretation, the Li quantification in this study shows that Li coverage at a predicted AV of 0.01 V is already very close to zero, whereas substantially greater coverage is observed once AV becomes negative. This near-threshold behavior supports the use of a control threshold above 0 V when the aim is not merely to delay severe plating, but to more conservatively avoid detectable plating.

To further assess the practical validity of this threshold, we performed a post hoc SEM analysis of the graphite electrode cycled under the 0.02 V smart-charging protocol shown in the **Fig. 4e&f** and **Fig. S11a&b**. Both grayscale-based and pixel-area-based quantification yielded 0% Li coverage for this protocol at end-of-life, supporting that the selected threshold successfully avoided detectable Li plating under the tested conditions.

Taken together, the 0.02 V threshold should be understood as a conservative engineering margin selected based on model uncertainty, false-negative analysis near the threshold, and experimental validation of plating-free behavior under the resulting control protocol.

Specific Comments R4-4. *The authors have substantially strengthened the SEM validation by expanding it to four AV conditions with n = 3 cells per condition and by adding quantitative lithium-coverage analysis, which clearly improves the manuscript. However, the quantitative validation still appears to rely mainly on a two-dimensional surface coverage metric. I would encourage the authors to provide more complete statistical information, such as raw per-sample coverage values, field-of-view variability, confidence intervals, and—if available—deposit size or thickness distributions. In addition, since the rebuttal emphasizes a clear correlation between predicted AV and the extent of lithium deposition, it would be valuable to support this claim with a more formal statistical analysis rather than a trend display alone.*

Authors' Response: We thank the Reviewer for this constructive suggestion. We agree that the SEM validation is strengthened by providing more complete statistical information beyond the mean surface-coverage trend alone. In response, we have expanded the quantitative analysis in the revised manuscript/Supplementary Information in several ways.

First, in addition to reporting the mean ± standard deviation across three cells per AV condition, we now provide the raw per-sample lithium-coverage values underlying each condition, together with confidence intervals. These additions make the dispersion of the SEM-based quantification more transparent. Here we provided the raw data table(included in the final raw data file), and analyzed the mean value, standard deviation, and the confidence interval (**Table R2** and **S6**).

Predicted AV Group	Coverage(%)-Pixel analysis	Coverage(%)-Greyscale analysis
**0.01 V**	0.08	0
	0.11	0
	0.06	0
**0 V**	0.11	2.45
	0.22	3.67
	0.59	4.89
**-0.01 V**	36.67	15.58
	21.22	10.94
	21.83	13.26
**-0.02 V**	43.40	46.01

	58.61	66.43
	50.95	56.22

Table R2. Raw data for Li coverage(%) calculated by pixel and greyscale analysis under different predicted AV

Predicted AV Group	Mean coverage (%)-pixel analysis	SD (%)-pixel analysis	95% CI (%)-pixel analysis	Mean coverage (%)-greyscale analysis	SD (%)-greyscale analysis	95% CI (%)-greyscale analysis
**0.01 V**	0.08	0.02	0.02 to 0.15	0	0	0.00 to 0.00
**0 V**	0.30	0.25	-0.32 to 0.93	3.67	1.22	0.64 to 6.70
**-0.01 V**	26.57	8.75	4.84 to 48.31	13.26	2.32	7.50 to 19.02
**-0.02 V**	50.99	7.60	32.09 to 69.88	56.22	10.21	30.86 to 81.58

Table S6. Mean value, standard deviation (SD), and confidence interval (CI) analysis of Li coverage (%) calculated by pixel-area and greyscale methods under different predicted AV conditions. The relatively wider CIs under negative AV conditions reflect the localized and heterogeneous nature of Li plating on the graphite interface, highlighting the importance of follow-up research on localized lithium plating.

We found that, under negative AV conditions, the confidence intervals become relatively wide, reflecting the **localized** and **heterogeneous** nature of Li plating on the graphite interface. This observation further underscores the importance of our follow-up research on local anode voltage mapping platform (**Figure.R3**), which may provide deeper insight into the spatially non-uniform behavior of lithium plating.

[Figure Redacted]

Second, to further characterize Li deposition morphology beyond total areal coverage, we have added a lateral Li deposit size distribution analysis based on the segmented plated-Li features in the top-view SEM images **(Fig. S19)**. It can be quantitatively observed that when the AV is 0.01 V and 0 V, only sparse lithium deposition is present. When the AV drops to -0.01 V, a large amount of lithium deposition appears, with particle sizes ranging from 3 to 12 μm. As the AV further decreases to -0.02 V, the lithium deposition evolves from isolated spots to widespread

surface coverage, with large lithium deposits appearing on the surface and particle sizes exceeding 70 μm. These measurements quantify the projected in-plane size distribution of Li deposits and provide an additional morphological descriptor of plating evolution with predicted AV.

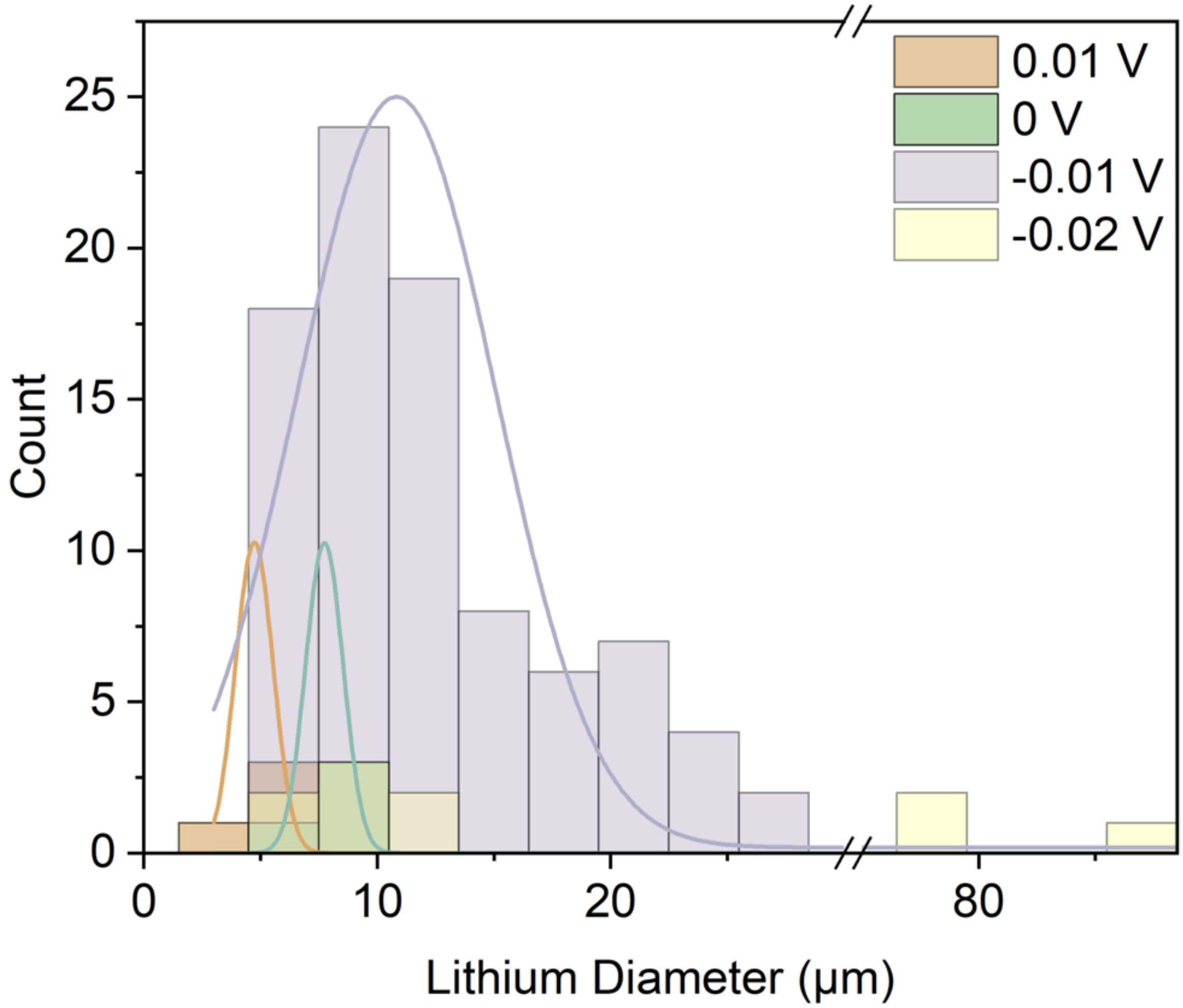

Figure S19. Li deposit size distribution analysis under different predicted AV. Results are collected from SEM images in Fig. S15. When the AV is 0.01 V and 0 V, only sparse lithium deposition is present. When the AV drops to -0.01 V, a large amount of lithium deposition appears, with particle sizes ranging from 3 to 12 μm. As the AV further decreases to -0.02 V, the lithium deposition evolves from isolated spots to widespread surface coverage, with large lithium deposits appearing on the surface and particle sizes exceeding 70 μm.

Third, to support the statement that predicted AV correlates with the extent of Li deposition, we supplemented the trend display with a formal statistical analysis. Specifically, using all individual samples across the four AV conditions, we evaluated the association between predicted AV and Li coverage using both Spearman rank correlation and Pearson correlation. Here, the correlation coefficient quantifies the direction and strength of the association, whereas the p-value indicates the statistical significance of that association under the null hypothesis of no correlation (**Table S7**). We found a strong inverse monotonic correlation by Spearman analysis (**Spearman's ρ = -0.950, p = 2.25 × 10^{-6}**) and a similarly strong inverse linear correlation by Pearson analysis (**Pearson's r = -0.924, p = 1.77 × 10^{-5}**). These results provide quantitative evidence that increasingly negative predicted AV is associated with increasingly severe Li deposition, beyond qualitative trend display alone. Using the same sample-level analysis for the greyscale-based quantification, we likewise observed a strong inverse monotonic correlation between predicted AV and Li coverage by Spearman analysis (**Spearman's ρ = -0.978**, **p = 3.50 × 10^{-8}**) and a strong inverse linear correlation by Pearson analysis (**Pearson's r = -0.873, p = 2.12 × 10^{-4}**). These results are consistent with the pixel-area analysis and further support that increasingly negative predicted AV is associated with increasingly severe Li deposition.

Li coverage quantification method	Spearman's ρ	p-value	Pearson's r	p-value
Pixel area analysis	-0.950	2.25 × 10^{-6}	-0.924	1.77 × 10^{-5}
Greyscale analysis	-0.978	3.50 × 10^{-8}	-0.873	2.12 × 10^{-4}

Table S7. Correlation analysis between predicted AV and Li coverage for different quantification methods

Overall, we believe the present SEM analysis provides strong support for our conclusion. Two independent Li-coverage quantification methods produced consistent results, including comparable group averages, standard deviations, and 95% confidence intervals across the four predicted AV conditions. Both methods showed negligible coverage at 0.01 V and 0 V, followed by a sharp increase at negative predicted AV. In addition, we further analyzed the lateral size distribution of segmented plated-Li features from the top-view SEM images **(Fig. S19)**. This analysis showed that at 0.01 V and 0 V, only sparse Li deposits were present, whereas at -0.01 V, substantial deposition emerged with characteristic lateral sizes of approximately 3-12 μm. As

the predicted AV further decreased to -0.02 V, the deposition evolved from isolated features to widespread surface coverage, with large deposits exceeding 70 μm. Thus, the SEM data not only show an increase in total Li coverage, but also reveal a clear progression in deposit morphology and characteristic feature size as predicted AV becomes more negative. Together with the strong correlation between predicted AV and Li coverage, these results provide converging statistical and morphological evidence that increasingly negative model-predicted anode voltage corresponds to increasingly severe lithium deposition.

Regarding the reviewer's request for thickness distributions, we agree that these measurements would provide valuable additional morphological information. However, the purpose of the present SEM dataset is to provide a brief but direct morphological validation that the model-predicted AV tracks the onset and progression of lithium plating. We also recognize that more extensive three-dimensional characterization and multi-field imaging would further strengthen this validation and represent important directions for future work.

Manuscript edit:

Main text, Page 13-14:

Li coverage is nearly zero when the predicted AV is 0.01 or 0 V, whereas it increases to >10% at a predicted AV of -0.01 V and further to >50% at -0.02 V (See methods Supplementary Fig. 15&16, and Table S6). Moreover, both pixel-area and greyscale analyses show a strong and statistically significant negative correlation between predicted AV and Li coverage (Table S7), confirming that lower predicted AV is associated with increased Li plating. Consistent with this trend, Li deposit size analysis further shows that the size of Li deposits increases as the predicted AV decreases (Supplementary Fig. 19). Together, these results further support that the model-predicted AV captures both the onset and the morphological progression of Li plating.

Supplementary information, Page 10-11:

The 95% confidence interval (CI) of the mean Li coverage was calculated using the Student's t-distribution:

$$CI_{95\%} = \bar{x} \pm t_{0.795,n-1} \frac{s}{\sqrt{n}}$$

where $\bar{x}$ is the mean Li coverage, s is the sample standard deviation, and n is the number of independent cell samples. In this study, n = 3 for each AV condition, so $t_{0.795,2}$ = 4.303. The resulting CI represents the uncertainty in the estimated mean Li coverage for each predicted AV condition.

To quantitatively evaluate the association between predicted anode voltage (AV) and Li coverage, all individual sample-level data points across the four AV conditions were included in the correlation analysis. For each Li-coverage quantification method, predicted AV values were paired with the corresponding Li coverage values from individual cell samples. Spearman rank correlation was used to assess the strength and direction of the monotonic relationship between predicted AV and Li coverage, without assuming a linear relationship. Pearson correlation was additionally calculated to evaluate the strength of the linear relationship between predicted AV and Li coverage. For both analyses, two-sided p-values were calculated under the null hypothesis of no correlation between predicted AV and Li coverage. The p-value represents the probability of observing a correlation at least as strong as the measured one if no true association exists; therefore, a smaller p-value indicates stronger statistical evidence against the null hypothesis. A negative correlation coefficient indicates that more negative predicted AV is associated with higher Li coverage. The correlation coefficients and corresponding p-values are summarized in Table S7.

Table S6. Mean value, standard deviation (SD), and confidence interval (CI) analysis of Li coverage (%) calculated by pixel-area and greyscale methods under different predicted AV conditions. The relatively wider CIs under negative AV conditions reflect the localized and heterogeneous nature of Li plating on the graphite interface, highlighting the importance of follow-up research on localized lithium plating.

Predicted AV Group	Mean coverage (%)-pixel analysis	SD (%)-pixel analysis	95% CI (%)-pixel analysis	Mean coverage (%)-greyscale analysis	SD (%)-greyscale analysis	95% CI (%)-greyscale analysis
**0.01 V**	0.08	0.02	0.02 to 0.15	0	0	0.00 to 0.00
**0 V**	0.30	0.25	-0.32 to 0.93	3.67	1.22	0.64 to 6.70
**-0.01 V**	26.57	8.75	4.84 to 48.31	13.26	2.32	7.50 to 19.02
**-0.02 V**	50.99	7.60	32.09 to 69.88	56.22	10.21	30.86 to 81.58

Table S7. Correlation analysis between predicted AV and Li coverage for different quantification methods*

Li coverage quantification method	Spearman's ρ	p-value	Pearson's r	p-value
Pixel area analysis	-0.950	2.25×10^{-6}	-0.924	1.77×10^{-5}
Greyscale analysis	-0.978	3.50×10^{-8}	-0.873	2.12×10^{-4}

Supplementary information, Page 25:

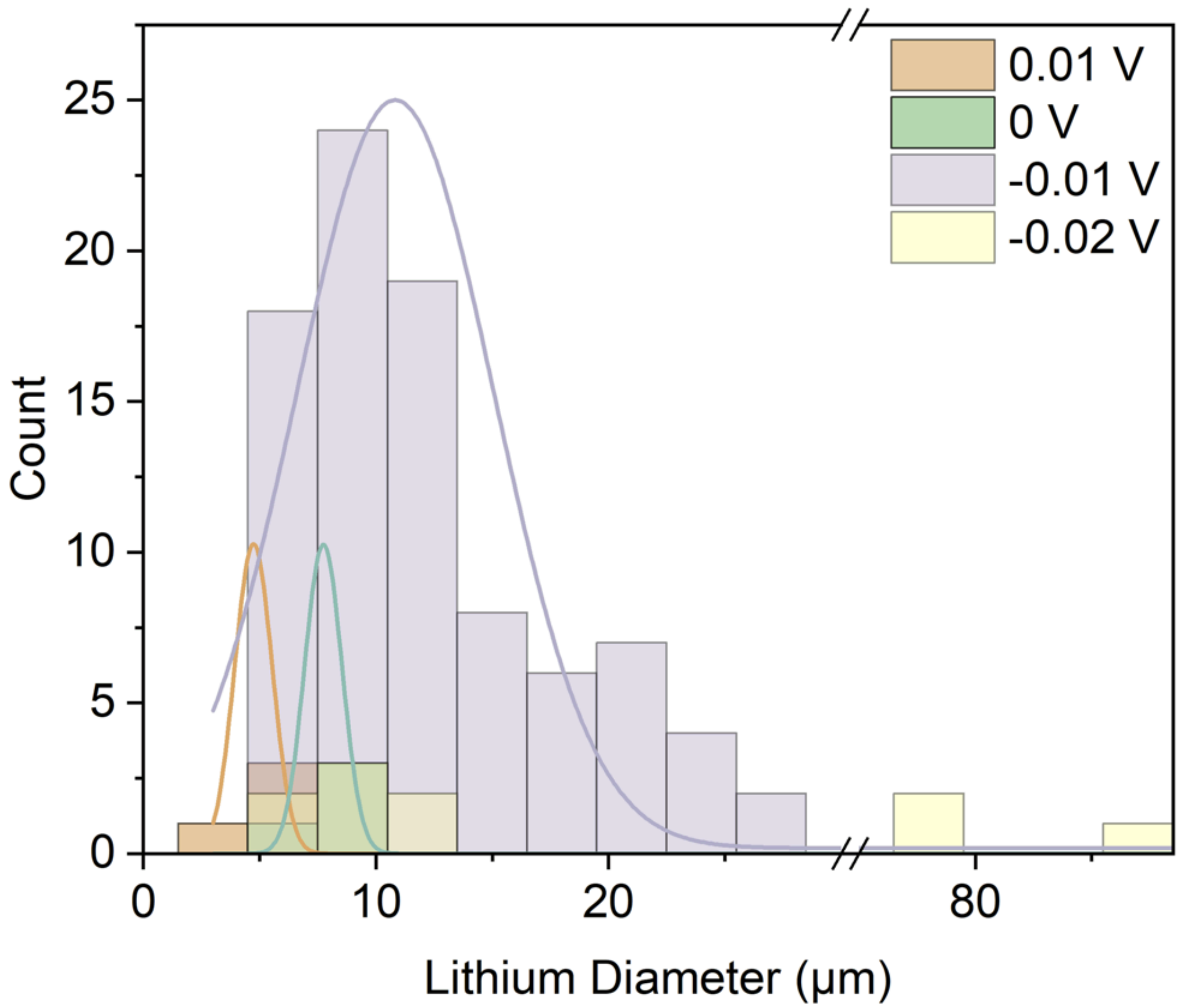

Figure S19. Li deposit size distribution analysis under different predicted AV. Results are collected from SEM images in Fig. S15. When the AV is 0.01 V and 0 V, only sparse lithium deposition is present. When the AV drops to -0.01 V, a large amount of lithium deposition appears, with particle sizes ranging from 3 to 12 μm. As the AV further decreases to -0.02 V, the lithium deposition evolves from isolated spots to widespread surface coverage, with large lithium deposits appearing on the surface and particle sizes exceeding 70 μm.

Specific Comments R4-5. *Although the final model uses only five input features, the authors employ a two-layer network with 256 hidden units per layer and an ensemble of five independently trained models. The rebuttal adds learning curves, seed variance, L1 regularization, and the qualitative explanation that the learned function requires a wide network; however, the justification remains somewhat empirical. I would encourage the authors to provide a more systematic ablation study, for example by comparing narrower architectures, non-deep-learning baselines such as linear or tree-based models, different lag-feature combinations, and different ensemble sizes. Such analyses would better demonstrate that the chosen architecture is not simply over-parameterized, but instead reflects a meaningful tradeoff among accuracy, robustness, and real-time deployability.*

Authors' Response: We thank the Reviewer for this constructive suggestion. While determining the optimal hyperparameters for our model, we ran an extensive set of experiments to test various parameters. Specifically, here we tested: Hidden Dim(**Table S10**), Number of hidden layers(**Table S11**), Number of ensemble models (**Figure.S13**), and a Tree-based model (**Figure. R3**), since linear regression has been included in previous **Fig.S4a**. Together, these ablation studies support the rationale for the selected final architecture, demonstrating that the model was not chosen arbitrarily or simply over-parameterized, but rather reflects a practical balance among prediction accuracy, robustness, model compactness, and real-time deployability.

Hidden Size	RMSE(V)	R^2
32	0.0472	0.73
96	0.0499	0.74
160	0.0443	0.76
224	0.0355	0.82
**256**	**0.0262**	**0.91**
288	0.0278	0.92
352	0.0311	0.86
416	0.0308	0.88
480	0.0373	0.80
544	0.0385	0.80

Table S10. Effect of hidden-layer dimension on validation performance during hyperparameter tuning. The highlighted row indicates the final model used in this study.

# of Layers	RMSE(V)	R^2
1	0.0512	0.71
2	0.0354	0.84
**3**	**0.0258**	**0.92**
4	0.0291	0.87

Table S11. Effect of Hidden layer number on validation performance during hyperparameter tuning. The highlighted row indicates the final model used in this study.

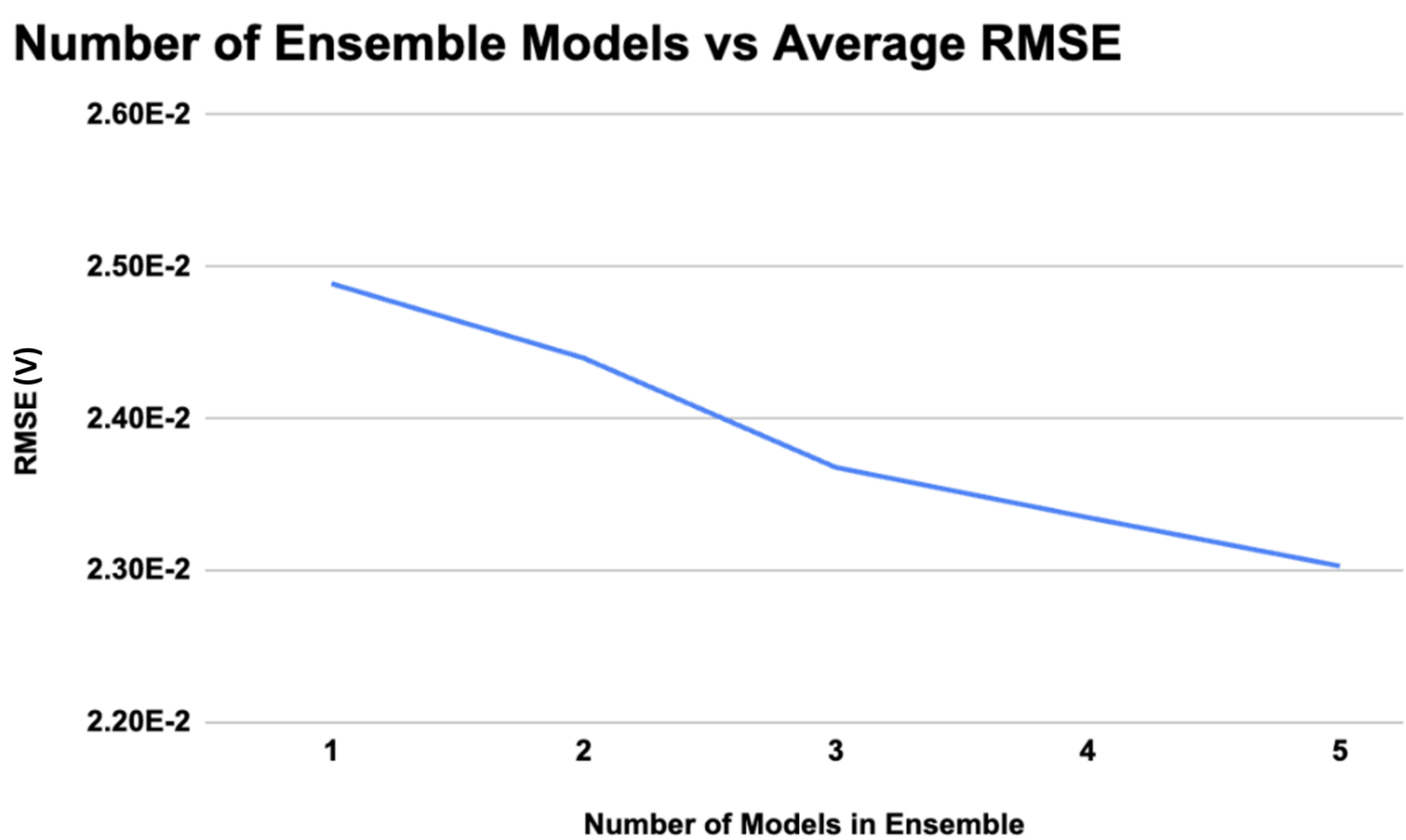

Figure S13. Number of Models in Ensemble vs. RMSE on test set. Results were averaged across multiple runs to reduce noise

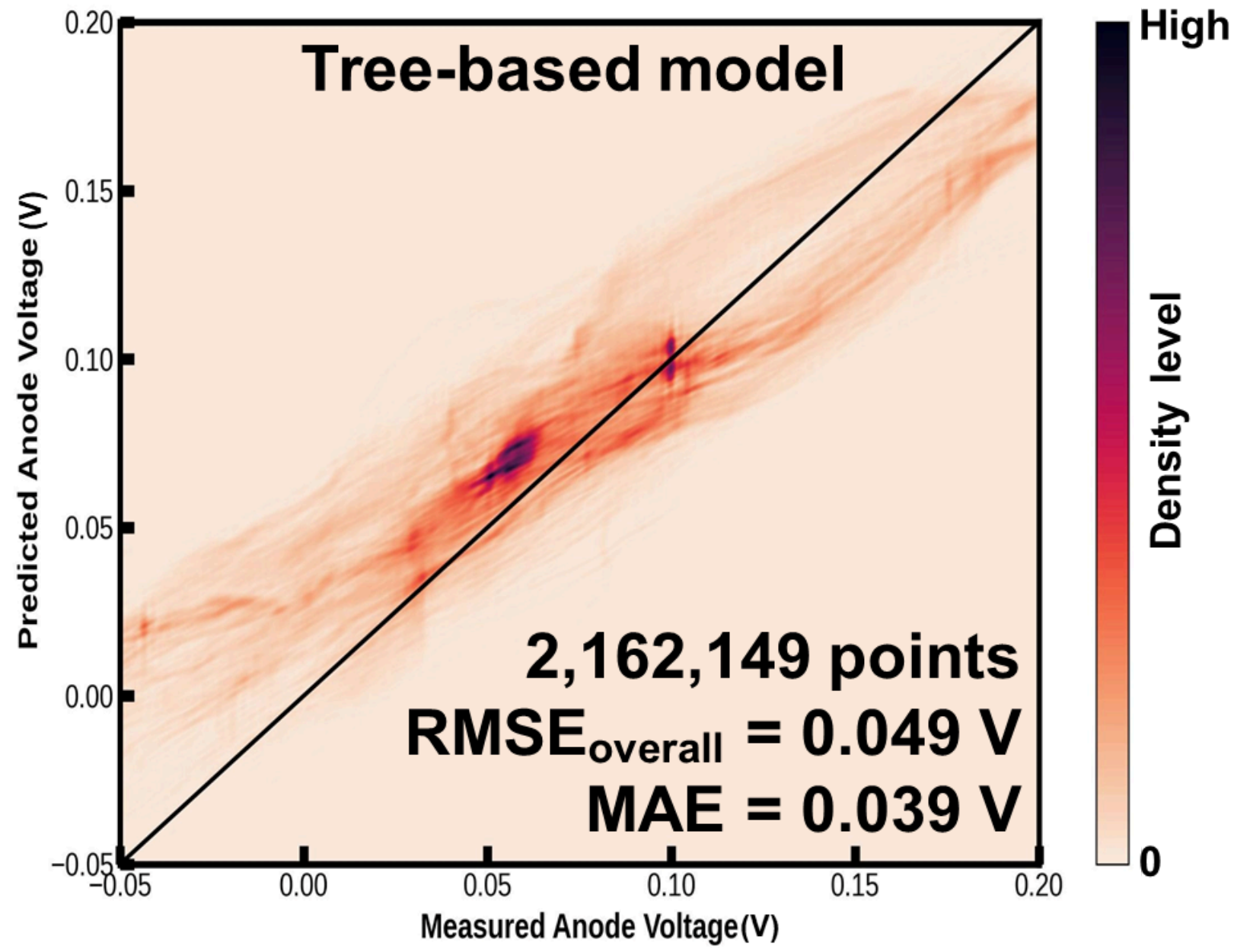

Figure R3. 2D density plot of predicted AV versus measured AV in all cells in test for tree-based model

As for the lagged-feature selection, the candidate feature pool included lagged versions of all basic and engineered features extending up to 8 s prior. The specific lagged features retained in the final model were not selected through manual cherry-picking, but instead through a systematic recursive feature elimination procedure during model development. We agree that, in principle, additional lag windows such as 10 s or 12 s could also be explored. However, the possible lag feature space is essentially open-ended, and exhaustive optimization over all possible lag durations is beyond the central scope of this work. Our goal here was not to identify a universally optimal temporal window, but to develop a compact, real-time compatible feature set that provides accurate anode voltage prediction while maintaining practical deployment efficiency. The 8 s upper bound, with lagged features considered at 2 s intervals, was therefore chosen as a reasonable short-history window consistent with the 2 s sampling interval used in the smart-charging scenario, and the final lagged feature combination was determined systematically within this predefined candidate space. This approach ensures that the selected lag features reflect an evidence-based balance among predictive relevance, model compactness, and real-time implementation feasibility, rather than an arbitrary or manually tuned choice.

Manuscript edit:

Main text, Page 8:

Model hyperparameters, including hidden-layer dimension, number of hidden layers, and ensemble size, were systematically tuned based on validation-set performance to identify a final architecture that balanced prediction accuracy and model compactness (see Hyperparameter Tuning in Supplementary information & Supplementary Fig. 13, and Table S10-11).

Main text, Page 8:

(3) time-lagged features (e.g., features that are measurable and engineered features from past timesteps up to 8 s prior, with lagged features considered at 2 s intervals consistent with the smart-charging sampling rate discussed later in the main text)

Supplementary information, Page 6-7:

Hyperparameter Tuning

Model hyper-parameters were chosen using a hyper-parameter tuning process. Specifically, hidden unit size, learning rate, dropout rate, batch size, and number of ensemble models were all varied to identify the best possible model construction. We considered hidden units between 32 and 544 evenly spaced by 64, learning rates 0.1, 0.01, 0.001, dropout rates of 0.2, 0.3, and 0.5, batch size 32, 64, 128, and 256, and 1-4 models for ensemble size. For hyperparameter tuning, the training set was further split (80/20) into a smaller training set and validation set used to pick between hyper-parameters. Hyperparameter tuning was done using the Keras Tuner framework using RMSE as the “validation metric” used to judge various models against each other.

Supplementary information, Page 7-8:

The tree-based model performs regression through a series of binary choices. We set the

maximum depth of our tree to be 3 layers deep and set the minimum number of samples in a leaf to 100 due to the large dataset size. We additionally used an ensemble of 5 trees as the training process can return widely different trees from the same dataset. The model was trained using scikit-learn in a regression manner.

Supplementary information, Page 32:

Table S10. Effect of hidden-layer dimension on validation performance during hyperparameter tuning. The highlighted row indicates the final model used in this study.

**Hidden Size**	**RMSE(V)**	**R^2**
32	0.0472	0.73
96	0.0499	0.74
160	0.0443	0.76
224	0.0355	0.82
**256**	**0.0262**	**0.91**
288	0.0278	0.92
352	0.0311	0.86
416	0.0308	0.88
480	0.0373	0.80
544	0.0385	0.80

Table S11. Effect of hidden layer number on validation performance during hyperparameter tuning. The highlighted row indicates the final model used in this study.

**# of Layers**	**RMSE(V)**	**R^2**
1	0.0512	0.71
2	0.0354	0.84
**3**	**0.0258**	**0.92**
4	0.0291	0.87

Supplementary information, Page 14:

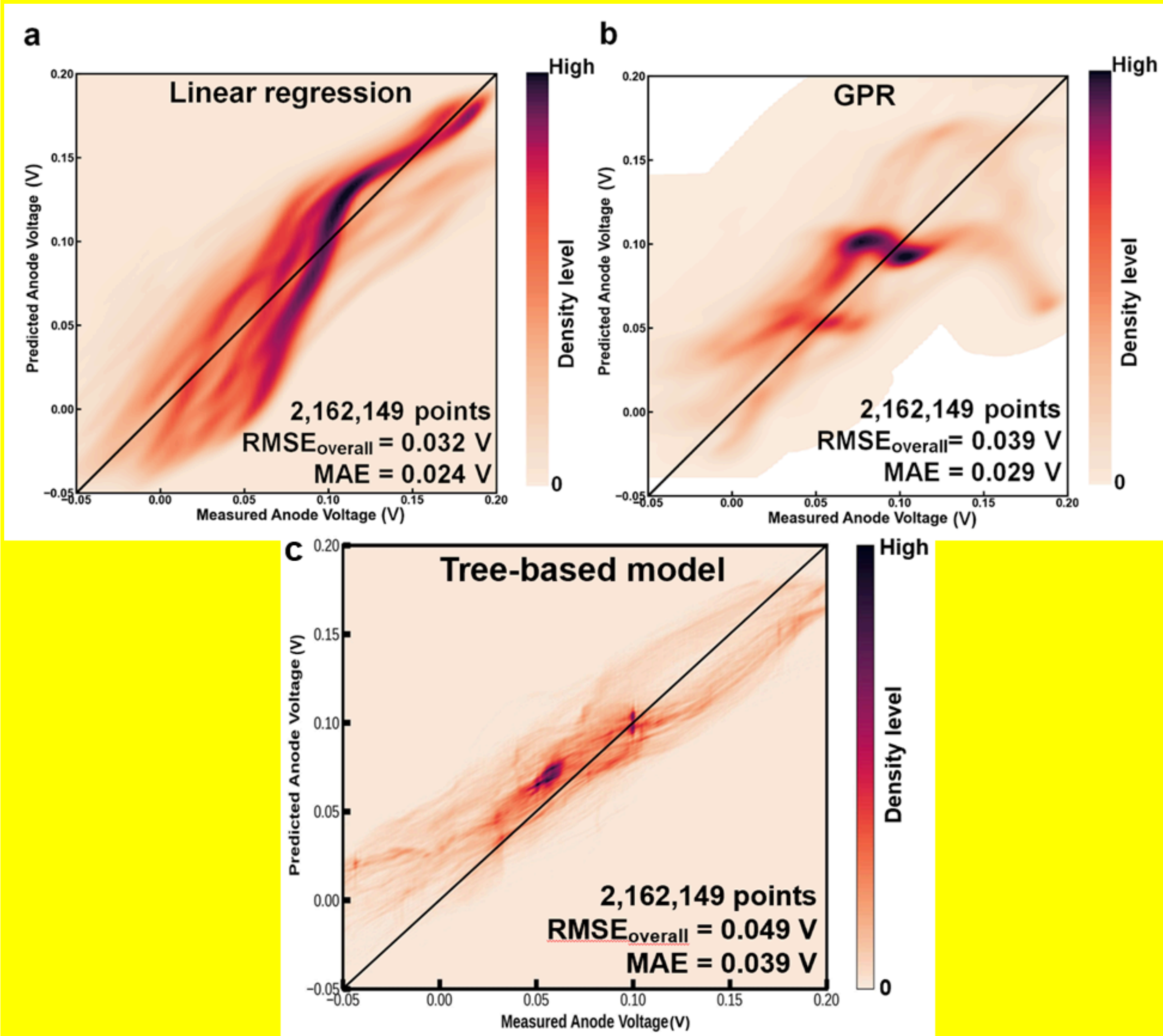

Figure S4. Performance of baseline models. 2D density plot of predicted AV versus measured AV in all cells in test for **a,** linear regression model; **b,** GPR model; **c**, Tree-based model

Specific Comments R4-6. *The additional estimate of an end-to-end closed-loop latency of about 115 ms, which is much smaller than the 2 s sampling interval, is helpful. However, for practical BMS deployment, average latency alone is not sufficient. A key concern is whether the system includes explicit fail-safe logic and fallback control policies under abnormal conditions such as communication jitter, packet loss, outlier inputs, abrupt prediction spikes, or model execution failure. The current rebuttal mainly reports average latency, but does not yet describe worst-case latency, anomaly handling, or conservative fallback strategies. I encourage the authors to provide these implementation details to better support the practical deployment claim.*

Authors' Response: We thank the Reviewer for this important comment. We agree that, for practical BMS deployment, explicit fallback controls and fail-safe logic are essential for safe battery operation, and that average latency alone is not sufficient to fully characterize deployment readiness. In our present implementation, the internal communication between the console TCP Interface and the Arbin battery management system (Arbin CTI) is provided by Arbin Instruments. The full code used to (1) continuously acquire current and voltage measurements from the cells through Arbin CTI, (2) perform feature engineering on the fly, (3) generate anode-voltage predictions using the trained machine-learning models, and (4) adjust charging current accordingly, will be made publicly available on GitHub. In addition, the closed-loop implementation was executed on a standard desktop-class Arbin control PC equipped with an integrated Intel UHD Graphics 630, without reliance on a dedicated high-performance GPU. This indicates that the reported latency and control performance are achievable on modest, non-specialized computing infrastructure.

To improve operational safety in the experimental implementation, the charging protocol remained constrained by cycler-level voltage and current limits, and the ML-based current adjustment was implemented within a predefined safe operating window. In cases of delayed communication, abnormal inputs, failed model execution, or abrupt nonphysical prediction behavior, the conservative fallback strategy is to stop ML-based current adjustment and return to a predefined safe CC-CV charging protocol rather than continuing closed-loop optimization. We have clarified this point in the revised manuscript. At the same time, we recognize that a more comprehensive treatment of worst-case latency, communication jitter, packet loss, outlier rejection, prediction-spike filtering, and automotive-grade fault handling will be important for future practical BMS integration. We therefore view these engineering safeguards as important next-step directions beyond the scope of the present study, while the current results establish the basic feasibility of real-time closed-loop implementation.

<u>Manuscript edit</u>:

Supplementary information, Page 8:

In the experimental implementation, the ML-based current regulation was constrained within predefined cycler-level voltage and current limits, and current updates were only applied within the safe operating window of the charging protocol. If delayed communication, abnormal input values, model execution failure, or abrupt nonphysical prediction behavior occurs, the control logic reverts from ML-based current adjustment to a predefined conservative CC-CV charging protocol.

Response Letter to Reviewers' Comments

Manuscript ID: NCOMMS-25-27724D

Title: Battery smart sensing via a virtual reference electrode

Authors: Jiayi Yu, Aki Takahashi, Min-Ho Kim, Rishi Upadhyay, Howard Zhang, Tian-Yu Wang, Huayang Zhu, Robert Kee, Tyrone Vincent, Bo Liu, Xintong Yuan, Thomas Hymel, Kaiyan Liang, Keyue Liang, Haoyang Wu, Dingyi Zhao, Jung Tae Kim, Achuta Kadambi✉, Yuzhang Li✉

Response to Reviewer #4

Specific Comments R4-1. *The added transfer-learning results are useful, but can the authors clarify how much target-domain data was used for fine-tuning, whether the split was strictly cell-level, and whether the original trained model has any zero-shot performance on the other LFP, NCM811, electrolyte, and pouch-cell datasets before transfer learning?*

Authors' Response: We thank the reviewer for this helpful comment. We agree that the protocol for the preliminary transfer-learning analysis in **Fig. R1** and **Table R1** should be clarified more explicitly.

As noted in our previous response, the transfer-learning results summarized in **Fig. R1** and **Table R1** were provided as additional response material to address the reviewer's concern regarding broader applicability, rather than as new main-text or Supplementary Information content. These results were intended to provide preliminary evidence that the framework can be adapted to additional chemistries, electrolyte formulations, and cell architectures, while a more comprehensive transfer-learning analysis is reserved for future follow-up work.

For clarity, the target-domain datasets used in this preliminary analysis included another LFP dataset from a different manufacturer/Argonne CAMP facility (8.7 M data points), an NCM811 dataset (8.0 M data points), an electrolyte-modified LFP dataset using 1 M $LiPF_6$ in EC/DEC (1:1) with 10% VC (3.4 M data points), and a three-electrode LFP pouch-cell dataset (1.4 M

data points). The results shown in **Fig. R1** and **Table R1** correspond to the performance after target-domain fine-tuning, with R^2 values of 0.90, 0.75, 0.93, and 0.87, respectively. **During fine-tuning, 70-80% of the data are used (the ratio is not fixed since we need to separate them strictly by individual cell)**

For each target domain, the model pretrained on the baseline LFP dataset was fine-tuned using target-domain data and then evaluated on held-out target-domain data. The data split was performed at the cell level wherever multiple cells were available, such that data from the same physical cell were not shared between the fine-tuning and testing sets. This cell-level splitting strategy was used to minimize information leakage between the adaptation and evaluation datasets.

We also evaluated the original pretrained model on the target-domain datasets before fine-tuning and observed noticeable zero-shot performance degradation, with R^2 values generally in the range of 0.3-0.5 before fine-tuning. This degradation indicates the presence of domain shift across chemistry, electrolyte formulation, and cell architecture. Therefore, we interpret **Fig. R1** and **Table R1** as preliminary evidence of adaptability after target-domain fine-tuning, rather than as a complete demonstration of zero-shot generalization. Because the target-domain datasets are still being expanded, the zero-shot and transfer-learning performance may change as additional cells, chemistries, and operating conditions become available. We agree that a systematic transfer-learning benchmark, including zero-shot degradation, target-data dependence, and broader validation across chemistries and cell formats, would be valuable; however, such an analysis is beyond the scope of the present manuscript and will be addressed in a dedicated follow-up transfer-learning study.

[Figure Redacted]

[Table Redacted]

Specific Comments R4-2. *The 0.02 V threshold reduces the false-negative rate below 1%, but what is the corresponding false-positive rate and impact on charging time/average charging current? Could the authors provide a threshold-sensitivity table including false negatives, false positives, charging speed, and cycle-life outcome if available?*

Authors' Response:

We thank the reviewer for this helpful suggestion. We agree that the threshold-sensitivity analysis should clarify both the safety-critical false-negative rate and the corresponding false-positive behavior.

In our smart-charging framework, the safety-critical failure mode is a false negative, defined as the condition where the real anode voltage is below 0 V, but the predicted anode voltage remains above the selected control threshold. Under this condition, the controller may incorrectly regard the cell as safe and fail to reduce the charging current. As shown in **Table S5**, increasing the threshold from 0 V to 0.01 V and 0.02 V reduced the false-negative rate from 2.140% to 1.445% and 0.947%, respectively.

We agree that the false-positive rate is also useful for evaluating the conservativeness of the threshold. In this context, a false positive corresponds to cases where the real anode voltage is above 0 V, but the predicted anode voltage falls below the selected threshold. This type of error does not represent a lithium-plating safety risk; rather, it corresponds to a conservative control action that may reduce the charging current even when the cell is not actually under plating conditions. We have therefore provided the corresponding false-positive analysis in **Table R3** of this response. Specifically, the false-positive conditions were defined as Real AV ≥ 0 V and Predicted AV < 0.02 V, Real AV ≥ 0 V and Predicted AV < 0.01 V, and Real AV ≥ 0 V and Predicted AV < 0 V for the 0.02 V, 0.01 V, and 0 V thresholds, respectively. The corresponding false-positive rates were 9.270%, 6.651%, and 4.716%.

Regarding charging time and average charging current, the closed-loop smart-charging experiments in the manuscript were performed using the 0.02 V cutoff. Therefore, the experimentally measured charging-speed behavior is available for this selected threshold, as shown in **Fig. S7**, where the smart-charging protocol maintained a higher average charging current density than the conventional CC-CV protocol over cycling. For the 0.01 V and 0 V thresholds, we do not have separate closed-loop cycling experiments. Because changing the threshold would alter the subsequent current trajectory, cell polarization, and anode-voltage

evolution, the corresponding charging time and average current cannot be rigorously determined from the measured-versus-predicted anode-voltage dataset alone.

We estimated the threshold-crossing behavior using the first-cycle predicted anode-voltage trajectory in **Fig. S12**.(Results are shown in **Table R2**) For the experimentally tested 0.02 V cutoff in **Fig.4**, the first-cycle average charging current density was 2.82 mA cm^{-2}. Lowering the cutoff to 0.01 V would delay the current-reduction event and is estimated to increase the average charging current density to approximately 2.9-3.0 mA cm^{-2}, while lowering the cutoff to 0 V would further increase it to approximately 3.1-3.2 mA cm^{-2}. These estimated speed gains, however, come at the cost of higher false-negative rates (**Table R2**).

The choice of the 0.02 V threshold is also consistent with the prediction uncertainty observed during closed-loop smart charging. As shown in **Fig. S12**, the model predicted the anode voltage with RMSE values of 20.5 mV in the 1st cycle and 22.7 mV in the 300th cycle. Therefore, using a 0.02 V cutoff provides an approximately one-RMSE safety buffer above the thermodynamic 0 V lithium-plating threshold. This buffer reduces cases where the real anode voltage becomes negative while the predicted anode voltage remains non-negative, thereby lowering the safety-critical false-negative rate.

Regarding cycle-life outcome, a systematic cycle-life comparison across multiple threshold values is not currently available, because such an analysis would require long-term closed-loop cycling experiments under each threshold condition. Therefore, we do not overinterpret the threshold dependence of cycle life here. Instead, the present analysis focuses on the immediate control-relevant tradeoff between false negatives, false positives, and estimated charging-speed impact. Overall, the 0.02 V threshold was selected as a safety-oriented operating point that reduces the false-negative rate below 1% while maintaining practical charging performance.

Table R2. False-negative analysis for smart-charging threshold settings

**False-negative condition**	**False-negative rate(%)**	**Average charging speed**
Real AV < 0 V && Predicted AV ≥ 0.02 V	0.947	2.82 mA cm^{-2}
Real AV < 0 V && Predicted AV ≥ 0.01 V	1.445	2.9-3.0 mA cm^{-2}
Real AV < 0 V && Predicted AV ≥ 0 V	2.140	3.1-3.2 mA cm^{-2}

Table R3. False-positive analysis for smart-charging threshold settings

False-positive condition	False-positive rate(%)
Real AV ≥ 0 V && Predicted AV < 0.02 V	9.270
Real AV ≥ 0 V && Predicted AV < 0.01 V	6.651
Real AV ≥ 0 V && Predicted AV < 0 V	4.716

Specific Comments R4-3. *Since localized plating may occur even when spatially averaged AV is slightly positive, can the authors clarify whether the false-negative analysis based on true AV < 0 V may underestimate missed localized plating events, and whether SEM data at small positive predicted AV values, e.g., 0.005–0.02 V, support the selected safety margin?*

Authors' Response: We thank the reviewer for this important comment. We agree that localized lithium plating may occur even when the spatially averaged anode voltage remains slightly positive, due to local heterogeneity in current density, electrode microstructure, transport, and interfacial kinetics. Therefore, a false-negative analysis based only on the condition of true AV < 0 V may underestimate the possibility of missed localized plating events.

This concern is one of the reasons why we selected a conservative positive threshold of 0.02 V, rather than using 0 V as the control cutoff. In our threshold-sensitivity analysis, the safety-critical false-negative condition for the 0.02 V cutoff was defined as Real AV < 0 V and Predicted AV ≥ 0.02 V. This condition represents cases where the true anode voltage has already entered the negative-AV regime, but the model prediction remains above the control threshold and therefore may not trigger current reduction. The rate of this false-negative condition was 0.947%, indicating that the 0.02 V threshold reduced missed negative-AV events to below 1%.

The 0.02 V threshold also provides a safety buffer above the thermodynamic lithium-plating condition and is comparable to the prediction uncertainty observed during smart charging. As shown in **Fig. S12**, the model predicted the anode voltage with RMSE values of 20.5 mV in the 1st cycle and 22.7 mV in the 300th cycle during closed-loop smart charging. Thus, using a 0.02 V threshold provides an approximately one-RMSE margin to reduce the likelihood that the true anode voltage becomes negative while the predicted anode voltage remains slightly positive.

Importantly, the SEM quantification supports this safety margin. As shown in **Fig. S16**, the quantified Li coverage was negligible at small positive and near-zero predicted anode voltages, including 0.01 V and 0 V, based on both grayscale and pixel-area analyses across three cells per condition. In contrast, Li coverage increased markedly once the predicted anode voltage became negative: the -0.01 V condition showed clear localized Li deposition, while the -0.02 V condition showed widespread Li coverage exceeding 50%. These results indicate that SEM-visible Li plating becomes pronounced in the negative-AV regime, while small positive and near-zero predicted AV conditions showed little observable Li coverage within the examined fields of view.

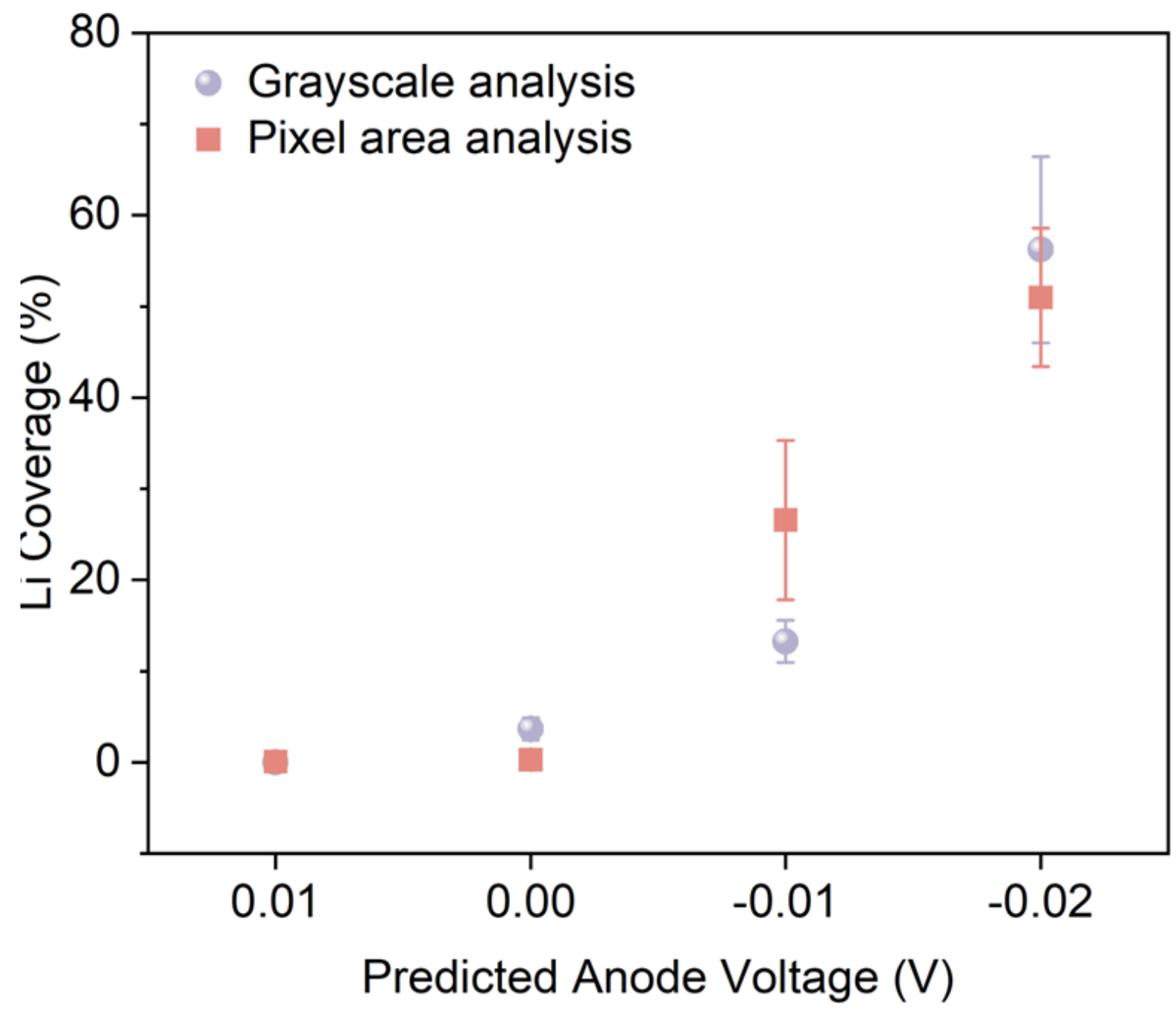

Figure S16. Li coverage with error bars for different predicted anode voltages. Li coverage was quantified from the SEM images in Fig. S15 via grayscale and pixel area analysis, by summing the pixel areas of all Li plating regions (including nuclei and large plating features, highlighted in red in Fig. S15) and normalizing by the total image pixel area. Data are presented as mean ± standard deviation across three cells per condition.

We have therefore clarified in Supplementary Note #3 that the false-negative analysis based on true AV < 0 V should be interpreted as a safety-relevant but spatially averaged metric, rather than a complete guarantee against all possible localized plating events. This clarification was added to avoid overinterpretation of the threshold analysis. The SEM data in **Fig. S16** supports the selected 0.02 V margin as a conservative operating threshold, because it provides a buffer before the negative-AV regime where Li coverage increases sharply. At the same time, we acknowledge that localized plating cannot be completely ruled out from a finite number of SEM fields of view. Overall, the 0.02 V cutoff was selected to provide a practical safety margin against both model prediction uncertainty and possible spatial heterogeneity in plating onset.

Manuscript edit:

Supplementary Information,Page 25:

Electrochemically, 0 V corresponds to the thermodynamic onset condition for Li plating. However, localized plating may already occur when the spatially averaged AV remains slightly positive because of electrode heterogeneity, local overpotentials, and kinetic effects[17,18]. Accordingly, in the present framework, AV < 0 V is interpreted as a physically meaningful plating-risk indicator rather than a strict binary classifier. Since the control objective is to more conservatively target Li-plating-free charging, the use of a threshold above 0 V is appropriate. We note that the false-negative analysis based on true AV < 0 V is therefore a safety-relevant metric based on the spatially averaged/effective AV, rather than a complete guarantee against all possible localized plating events.

Supplementary Information,Page 26:

This conservative choice is further supported by prior literature showing that early plating nuclei or residual plated Li can facilitate subsequent Li deposition during later cycling, making suppression of even very small initial plating events desirable[19]. Consistent with this interpretation, the Li quantification in this study shows that Li coverage at a predicted AV of 0.01 V is already very close to zero, whereas substantially greater coverage is observed once AV becomes negative. As shown in Fig. S16, this trend was observed using both grayscale-based and pixel-area-based analyses across three cells per condition, supporting that small positive and near-zero predicted AV conditions showed little detectable Li coverage within the examined fields of view. This near-threshold behavior supports the use of a control threshold above 0 V when the aim is not merely to delay severe plating, but to more conservatively avoid detectable plating.

Specific Comments R4-4. *The added SEM quantification is valuable, but can the authors report field-of-view-level variability or clarify how many SEM fields were analyzed per cell? Also, because the correlation analysis uses only four AV levels with three cells each, can the authors confirm that the p-values are not inflated by treating repeated AV-level observations as fully independent?*

Authors' Response: We thank the reviewer for this helpful comment. We agree that the SEM quantification and correlation analysis should clearly distinguish the SEM imaging/quantification region from independent biological replicates.

For clarity, each predicted-AV condition included three independently cycled cells. For each cell, the SEM analysis was performed at the same representative location near the edge of the inner graphite ring, where Li plating is most readily observed in this cell configuration. This consistent sampling location was selected to reduce variability caused by position-dependent morphology and to improve comparability across cells and AV conditions. The Li coverage was then quantified from the SEM images using both pixel-area and grayscale-based methods. The reported mean, standard deviation, and 95% confidence interval in **Table S6** were calculated across the three independent cells for each predicted-AV condition.

We have clarified this SEM sampling strategy in the revised Supplementary Information. Specifically, the independent replicate number is $n = 3$ cells per predicted-AV condition. The wider confidence intervals under negative-AV conditions, particularly at -0.01 V and -0.02 V, reflect the localized and heterogeneous nature of Li plating across independently cycled cells.

We also agree with the reviewer that the correlation analysis should be interpreted carefully because the dataset contains four predicted-AV levels with three independent cells per level. The correlation analysis in **Table S7** was performed using cell-level Li-coverage values, not by treating multiple SEM fields from the same cell as independent data points. Therefore, the p-values are not inflated by field-of-view pseudoreplication. However, because the analysis is based on four AV levels, we have revised the text to avoid overinterpreting the p-values and to emphasize the effect size and monotonic trend instead. Specifically, both quantification methods showed a strong negative association between predicted AV and Li coverage, with Spearman's $\rho = -0.950$ for pixel-area analysis and $\rho = -0.978$ for grayscale analysis.

Overall, these revisions clarify that the SEM quantification was based on three independently cycled cells per condition, that the same representative graphite inner-ring edge region was analyzed for consistency, and that the correlation results are intended as trend-supporting evidence rather than as an overpowered statistical claim. The main conclusion remains unchanged: Li coverage was negligible at small positive and near-zero predicted AV values, while it increased markedly once the predicted AV became negative.

Manuscript edit:

Methods section in main text, Page 22:

Owing to variations in brightness and contrast among SEM images, an image-specific grayscale threshold was selected to represent lithium-plated regions. Lithium coverage was then quantified as the area fraction of pixels exceeding this threshold relative to the total image area. For each predicted AV condition, three independently cycled cells were analyzed. SEM quantification was performed at the same representative region near the edge of the inner graphite ring for each cell, where Li plating is most readily observed, to improve comparability across cells and AV conditions.

Methods section in main text, Page 23:

The correlation coefficients and corresponding p-values are summarized in Table S7. SEM imaging regions were not treated as additional independent replicates. Because the analysis includes four predicted AV levels with three independent cells per level, the p-values should be interpreted as supporting the observed monotonic trend rather than as a highly powered statistical test

Specific Comments R4-5. *The ablation study supports the final network, but can the authors reconcile the stated architecture description with Table S11 and provide a compact table comparing final ENN, single NN, linear/GPR/tree baselines in terms of RMSE, MAE, inference latency, parameter count, and memory footprint under the same test set?*

Authors' Response:

We thank the reviewer for pointing this out. We agree that the hyperparameter-tuning description did not explicitly state that the number of hidden layers was included in the tuning/ablation process, which could make the relationship between the architecture description and **Table S11** unclear. We have revised the Hyperparameter Tuning section in SI to state that the number of hidden layers was varied together with hidden unit size, learning rate, dropout rate, batch size, and ensemble size. We have also clarified in the **Table S11** column that "# of Layers" refers to the number of hidden layers in each feedforward neural network. The highlighted 3-layer row in **Table S11** corresponds to the final architecture described in the Methods. This clarification does not affect the model architecture, reported performance, or conclusions.

Here we also provided a compact table comparing final ENN, single NN, linear/GPR/tree baselines in terms of RMSE, MAE, inference latency, parameter count, and memory footprint under the same test set. (**Table S12**)

**Model**	RMSE(V)	MAE(V)	Latency (ms)	# of Parameters	Memory (MiB)
**Ensemble NN**	0.023	0.018	0.51	133K	13.7
**Single NN**	0.025	0.019	0.11	666K	11.7
**Linear Fit**	0.032	0.024	0.01	6	<1
**GPR**	0.039	0.029	0.05	325K	1.2
**Regression Tree**	0.049	0.039	0.02	350K	15.1

Table S12: Comparison of baselines and proposed model across RMSE, MAE, Latency, Parameter count, and Memory. All latencies and memory values were measured on a RTX3090.

Manuscript edit:

Methods section in main text, Page 19:

Hyperparameter Tuning

Model hyper-parameters were chosen using a hyper-parameter tuning process. Specifically, hidden unit size, number of hidden layers, learning rate, dropout rate, batch size, and number of ensemble models were all varied to identify the best possible model construction. We considered hidden units between 32 and 544 evenly spaced by 64, 1-4 hidden layers, learning rates 0.1, 0.01, 0.001, dropout rates of 0.2, 0.3, and 0.5, batch size 32, 64, 128, and 256, and 1-5 models for ensemble size.

Supplementary Information, Page 21:

Table S11. Effect of hidden layer number on validation performance during hyperparameter tuning. The highlighted row indicates the final model used in this study.

# of hidden Layers	RMSE(V)	R^2
1	0.0512	0.71
2	0.0354	0.84
**3**	**0.0258**	**0.92**
4	0.0291	0.87

Supplementary Information, Page 22:

Model	RMSE(V)	MAE(V)	Latency (ms)	# of Parameters	Memory (MiB)
**Ensemble NN**	0.023	0.018	0.51	133K	13.7
**Single NN**	0.025	0.019	0.11	666K	11.7
**Linear Fit**	0.032	0.024	0.01	6	<1
**GPR**	0.039	0.029	0.05	325K	1.2
**Regression Tree**	0.049	0.039	0.02	350K	15.1

Table S12: Comparison of baselines and proposed model across RMSE, MAE, Latency, Parameter count, and Memory. All latencies and memory values were measured on a RTX3090.
